# Discovery of a Novel sp^3^‑Rich M_1_ Positive Allosteric Modulators (PAMs) Chemotype via Scaffold Hopping

**DOI:** 10.1021/acsmedchemlett.5c00271

**Published:** 2025-06-17

**Authors:** Joseph D. Bungard, Paul Spearing, Yu Nishio, Upendra Rathnayake, Chris C. Presley, Sichen Chang, Haley E. Kling, Analisa D. Thompson, Hyekyung P. Cho, Li Peng, Alice L. Rodriguez, Colleen M. Niswender, Olivier Boutaud, Valerie Kramlinger, Carrie K. Jones, P. Jeffrey Conn, Julie L. Engers, Darren W. Engers, Craig W. Lindsley, Changho Han

**Affiliations:** † Warren Center for Neuroscience Drug Discovery, 5718 Vanderbilt University, Nashville, Tennessee 37232, United States; ‡ Department of Pharmacology, 12327 Vanderbilt University School of Medicine, Nashville, Tennessee 37232, United States; § Vanderbilt Brain Institute, 12327 Vanderbilt University School of Medicine, Nashville, Tennessee 37232, United States; ∥ Vanderbilt Kennedy Center, 12328 Vanderbilt University Medical Center, Nashville, Tennessee 37232, United States; ⊥ Department of Chemistry, 5718 Vanderbilt University, Nashville, Tennessee 37232, United States

**Keywords:** GPCR, positive allosteric modulator, M_1_ PAM, pyrazole, Fsp^3^

## Abstract

The M_1_ receptor has long been investigated as a promising CNS drug target, yet further research is essential to fully elucidate compound’s Pharmacodynamic (PD) as well as Toxicokinetic (TK) effects. In this context, the development of structurally diverse and high-profile M_1_ PAM tool compounds remains highly valuable, as existing advanced tools exhibit notable structural similarity. One approach that can be considered during scaffold hopping exercise and can improve drug-like properties is to introduce additional sp^3^ carbon atoms and increase Fsp^3^ values; the fraction of sp^3^ hybridized carbons. Determining the correct location to incorporate sp^3^ carbon atoms can be challenging, but once the right position is identified, it often leads to novel optimization opportunities. Reported herein is the discovery of a novel sp^3^-rich M_1_ positive allosteric modulator series utilizing a *N*-cyclopentyl pyrazole core. Also, an iterative library synthesis approach provided an enhanced understanding of the minimum pharmacophore. Several compounds within the series showed favorable on-target potencies and DMPK properties. In conclusion, the reported sp^3^-rich *N*-cyclopentyl pyrazole-based M_1_ PAM scaffold offers a promising structure–activity relationship starting point to discover structurally distinct M_1_ PAM chemotypes.

## Graphical abstract

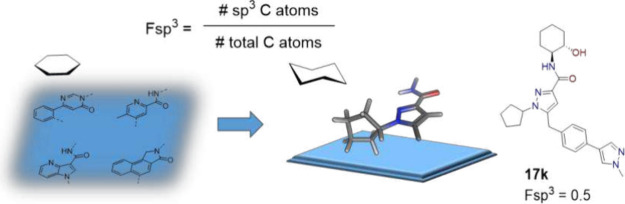

Alzheimer’s disease (AD) is a neurodegenerative disease that affects millions of individuals worldwide and accounts for roughly 60–80% of dementia cases.^1^ As Alzheimer’s disease progresses, various pathological changes provide logical evidence for cognitive decline and memory loss. These events include aggregation of amyloid β (Aβ), production of neurofibrillary tangles (NFT) from phosphorylation of tau, and reduction in the synthesis of acetylcholine (ACh).^2–4^ The cholinergic system has long been considered a target for the treatment of AD and other neurological disorders due to the ubiquitous nature of ACh within the central nervous system (CNS), and its role in pathways related to memory and cognition.^5^ Cholinergic signal transduction is mediated by muscarinic and nicotinic ACh receptors, and muscarinic ACh receptors (mAChRs) are considered important drug targets.^6^

There are five different subtypes of G protein-coupled mAChRs.^6^ Of the five mAChR subtypes M_1_–M_5_, M_1_ is most abundantly expressed in the brain, particularly in the hippocampus and prefrontal cortex, locations associated with memory and cognition.^7,8^ Additionally, selective activation of M_1_ enhances α-secretase activity which promotes the development of sAPPα, a protein that prevents the formation of Aβ plaques and makes M_1_ an attractive target for neurodegenerative diseases, such as AD.^9^

Since Xanomeline, an M_1_/M_4_ preferring agonist, was first discovered in the early 1990s,^10^ much effort has been made to discover highly selective M_1_ activators and potentiators. As a result, various subtype-selective M_1_ positive allosteric modulators (PAMs) have been reported (Figure 1).^11–22^ These chemotypes can be classified into three different categories: 1) chemotypes that utilize an intramolecular hydrogen bond to orient the amide carbonyl moiety, a key pharmacophore, in a bioactive conformation; 2) chemotypes that cyclize the amide to form a formalized ring; 3) structurally distinct chemotypes. However, selective M_1_ PAM compounds without undesired adverse events remain elusive.

**Figure 1:**
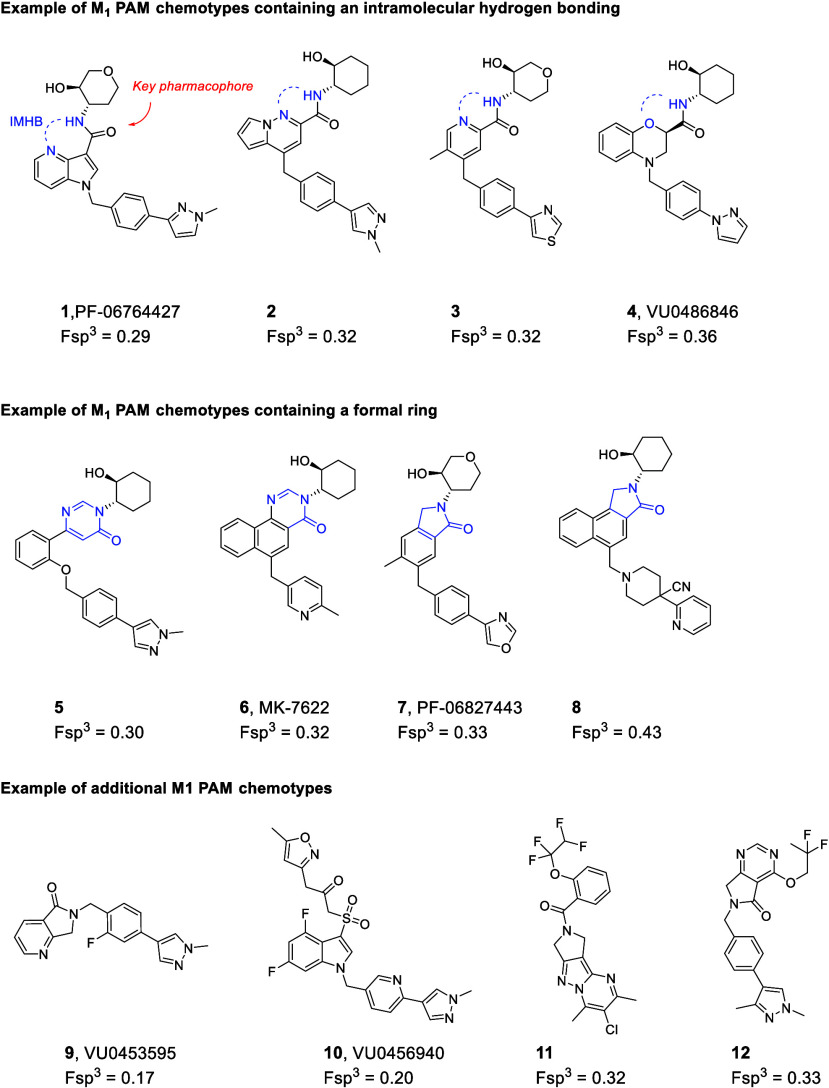
Selected M_1_ PAMs that have been reported in the public domain and their Fsp^3^ values; **1** ^11^, **2** ^12^, **3** ^13^, **4** ^14^, **5** ^15^, **6** ^16^, **7** ^17^, **8** ^18^, **9** ^19^, **10** ^20^, **11** ^21^, and **12** ^22^.

As shown in Figure 1, the majority of reported M_1_ PAM chemotypes share structural similarities except for a few such as **9**–**12**, and most of these chemotypes share low Fsp^3^, a recently highlighted drug-likeness parameter that describes the fraction of sp^3^ hybridized carbons in a drug molecule.^23,30^ Although a higher Fsp^3^ value by itself cannot serve as a predictor of overall drug-likeness and in vivo efficacy, over 80% of marketed drugs exhibit Fsp^3^ ≥ 0.42,^24^ indicating the potential importance of Fsp^3^ as a parameter to consider during the lead optimization stage. Thus far, only a small number of M_1_ PAMs have Fsp^3^ ≥ 0.42. Moreover, many advanced M_1_ PAM tool compounds are structurally related, sharing only a narrow chemical space. Given the complex nature of M_1_ pharmacology and M_1_-mediated adverse events, a better understanding of M_1_ biology through potent, selective, yet structurally diverse M_1_ PAM tools is essential for successful clinical trials.

To date, numerous scaffold-hopping efforts have been made in M_1_ PAM discovery to identify novel chemotypes. However, introducing an sp^3^-rich moiety into the molecule has proven quite difficult, as the M_1_ PAM binding site predominantly accommodates chemotypes with sp^2^-hybridized structures. Despite these challenges, our efforts to find a new starting point continued, as a fundamentally different scaffold containing a unique sp^3^-rich core could provide us with unique opportunities and ADMET profiles.

Recently, scientists at Monash Institute for Pharmaceutical Sciences (MIPS) reported an interesting 6-phenylpyrimidin-4­(*3H*)-one-based scaffold, as featured in **5** (Figure 1), which evolved from 1-(4-methoxybenzyl)-4-oxo-1,4-dihydroquinoline-3-carboxylic acid (BQCA).^15^ As shown in Figure 2, we hypothesized that a novel chemotype with a potential new SAR point could be generated from a scaffold hopping exercise (or hybridization exercise) between **3** and **5**. During the rationalized design process, we suspected that the core size might require reduction as a rather large phenyl ring had been introduced. Therefore, the pyrazole ring was designed instead of the pyridine ring. In general, pyrazoles are metabolically stable and interact with biological targets through hydrogen bonding, making them versatile structures in the realm of medicinal chemistry.^25^ We envisioned that this new chemotype could not only provide unique SAR opportunities but also provide a desirable steric repulsion against the benzylic tail. Additionally, the nitrogen atom from the pyrazole ring would allow intramolecular hydrogen bond formation with the amide N–H that stabilizes the bioactive conformer.

**Figure 2:**
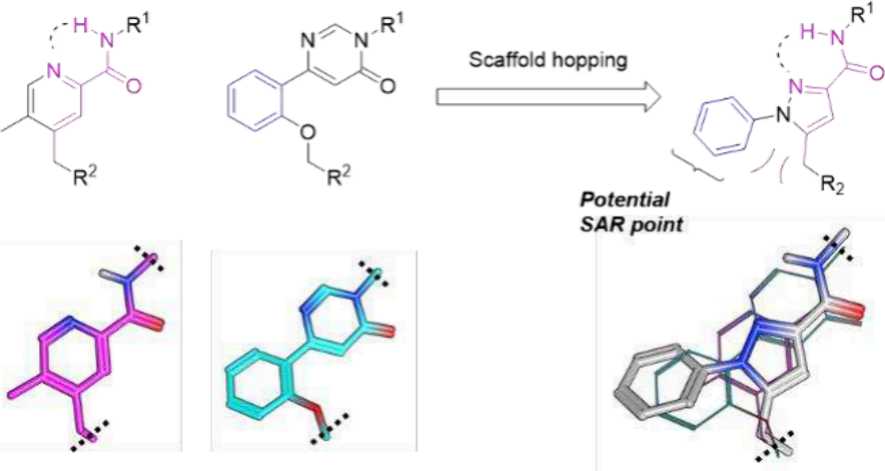
Rationalized design of the novel M_1_ mAChR PAM scaffold contains a potential new SAR opportunity.

The synthesis of the designed novel core was straightforward (Scheme 1). The synthesis commenced through the treatment of diketoester **13** with various alkyl hydrazines in EtOH at 80 °C to provide the corresponding alkylated pyrazole subunits in **14** in good yield (64–84%). The resulting olefins were then oxidatively cleaved with OsO_4_ and NMO and subsequently treated with NaIO_4_ to provide the corresponding aldehyde intermediates. Reduction of the aldehydes with NaBH_4_ in MeOH at 0 °C furnished the resulting pyrazole alcohols **15** in good yields. Conversion of the alcohols to the bromide derivative with PBr_3_ at 0 °C, followed by a Suzuki-Miyaura coupling with the appropriate boronic ester and Pd­(dppf)­Cl_2_·DCM at 80 °C provided analogs **16**. Hydrolysis of the ethyl ester and HATU-mediated amide coupling reactions afforded the M_1_ PAM analogs **17** in yields of 11–61% over the course of two steps.

**Scheme 1:**
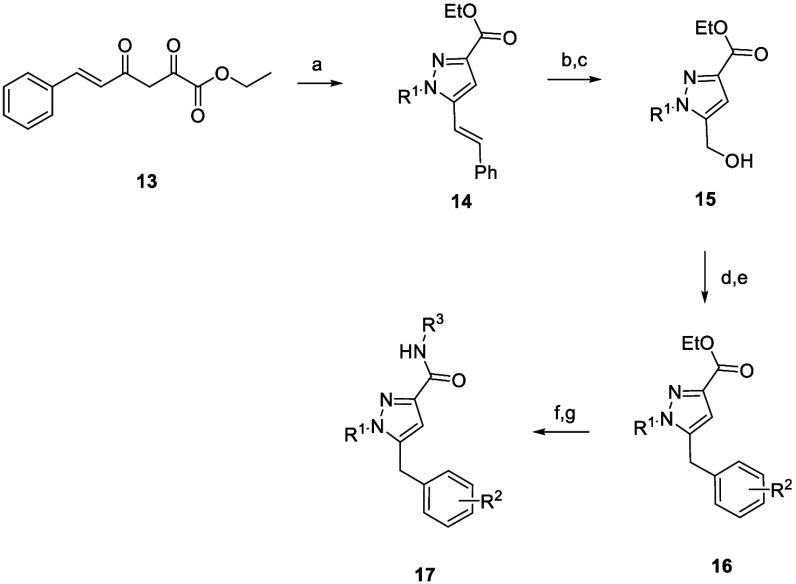
Synthesis of Pyrazole Analogs **17** ^a^

a Reagents and conditions: (a) R^1^NHNH_2_·HCl, EtOH, 80 °C, 2 h, 64-84%; (b) OsO_4_, NMO, THF/H_2_O, 0 °C–RT overnight, then NaIO_4_; (c) NaBH_4_, MeOH, 0 °C, 2 h, 80-88% over 2 steps; (d) PBr_3_, DCM, 0 °C, 1 h; (e) Aryl boronic ester, Pd­(dppf)­Cl_2_·DCM, Cs_2_CO_3_, 1,4-Dioxane/H_2_O, 80 °C, 13–28% over 2 steps; (f) LiOH, THF, RT overnight, quantitative; (g) R^3^NH_2_, HATU, DIPEA, DMF, RT, 11–61% over 2 steps.

Because of the size of the phenyl ring, we suspected the binding pose could be frame-shifted to the right (Figure 2). In this case, the historically preferred cyclohexanol and tetrahydro-2*H*-pyran-3-ol next to the amide linkage may not be the optimal substituent, and a smaller ring might be more desirable. Therefore, we synthesized a set of compounds with a variety of ring sizes (Table 1). Because rats are used in our preclinical pharmacodynamic models, the ideal chemotype should not have a huge species disconnect. Therefore, newly synthesized compounds were then tested against both human and rat M_1_ mAChrs in parallel.

**Table 1:**
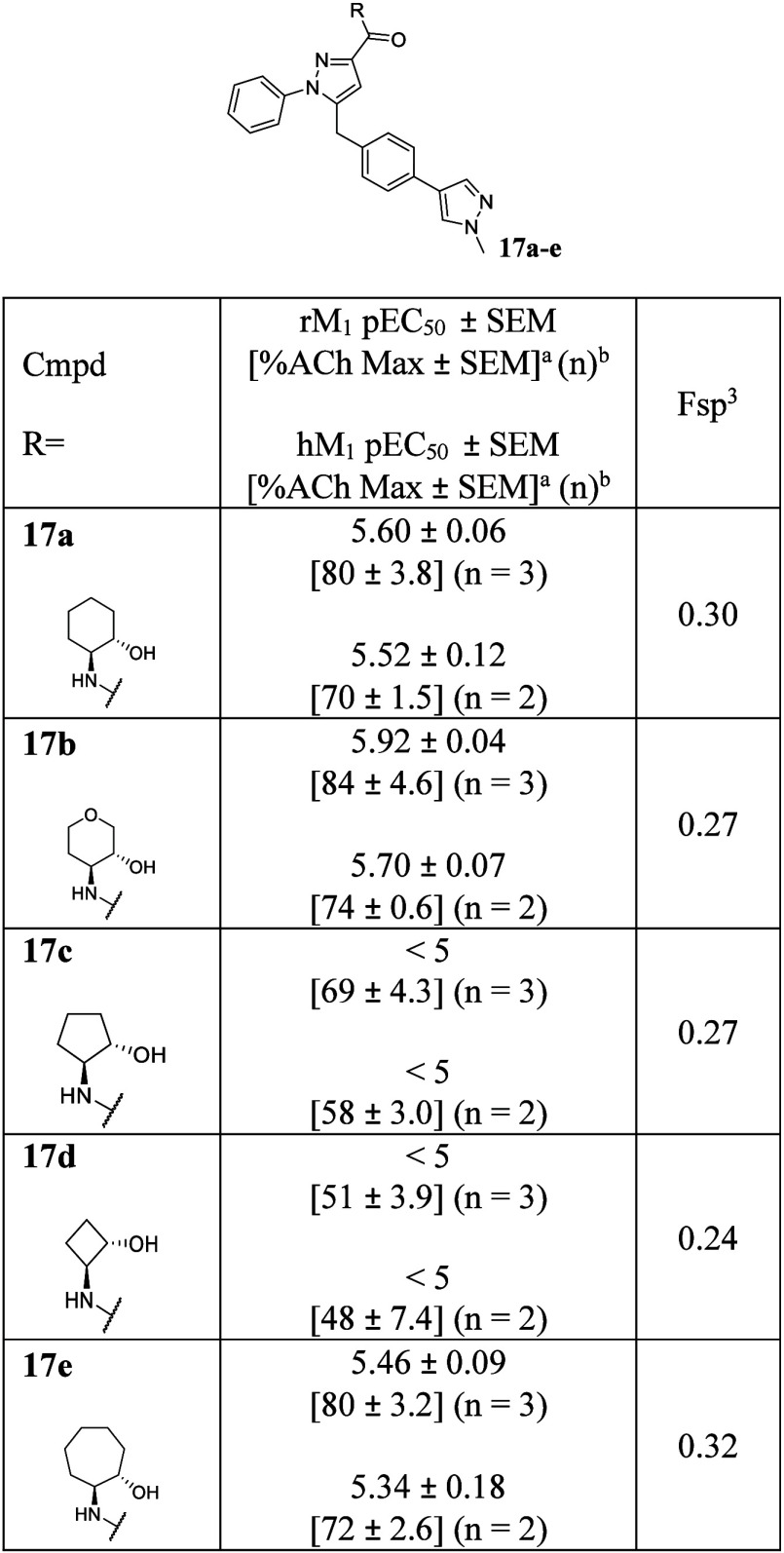
Initial Compound Library and SAR Evaluation of *N*-Phenyl Pyrazoles

a Calcium mobilization assays in rM_1_ and hM_1_-CHO cells were performed in the presence of an EC_20_ fixed concentration of acetylcholine for PAM activity.

b Each independent experiment was performed in duplicate or triplicate.

Despite the sensitive SAR nature of the PAM binding site, **17a** and **17b** retained modest potencies in the low micromolar range (hM_1_ pEC_50_ = 5.52 ± 0.12 and 5.70 ± 0.07, respectively). In contrast to our hypothesis, smaller rings containing analogs **17c** and **17d** displayed a slight loss of potency (hM_1_ pEC_50_ < 5). This potentially indicates that the binding pose of analogs containing an *N*-phenyl pyrazole core may still be similar to those of the historical compounds or may be unexpectedly shifted to the left. To confirm our hypothesis, we synthesized a larger ring containing analog **17e**. It was slightly less potent compared to **17a** and **17b,** indicating that the binding pose might remain very similar to the previously reported series (**17e**, hM_1_ pEC_50_ = 5.34 ± 0.18). From these results, we maintained­(3*R*,4*S*)-4-aminotetrahydro-2*H*-pyran-3-ol as an optimal substituent and shifted attention to surveying additional substituents from the pyrazole core.

Since π–π interactions have been revealed as major driving forces between GPCR allosteric sites and PAM ligands (Figure 3),^26^ only flat and aromatic motifs tend to be considered as alternative cores, assuming M_1_ PAMs may have similar binding poses compared to **LY2119620**. Indeed, subsequent structure–activity relationship studies as well as docking studies in the allosteric site of the M_1_ homology model suggested M_1_ PAMs may have similar binding poses compared to **LY2119620**.^13^ Therefore, we initially speculated that the *N*-phenyl pyrazole moiety was essential for activity because it is also flat and aromatic. In addition, only small aromatic heterocycles that have minimal effect on the π–π interaction between the core and tryptophan residue may be tolerated as *N*-pyrazole substituents. However, as shown in Table 1, Fsp^3^ values of *N*-phenyl pyrazole analogs were already much lower than 0.42 in general (0.24–0.32). Therefore, to improve pharmaceutical properties by enhancing the sp^3^ character, we decided to challenge the aforementioned dogma and introduce the sp^3^ character off of the pyrazole core. Besides, there is always a possibility that different M_1_ PAM chemotypes could have slightly different binding poses and interact with different residues.

**Figure 3:**
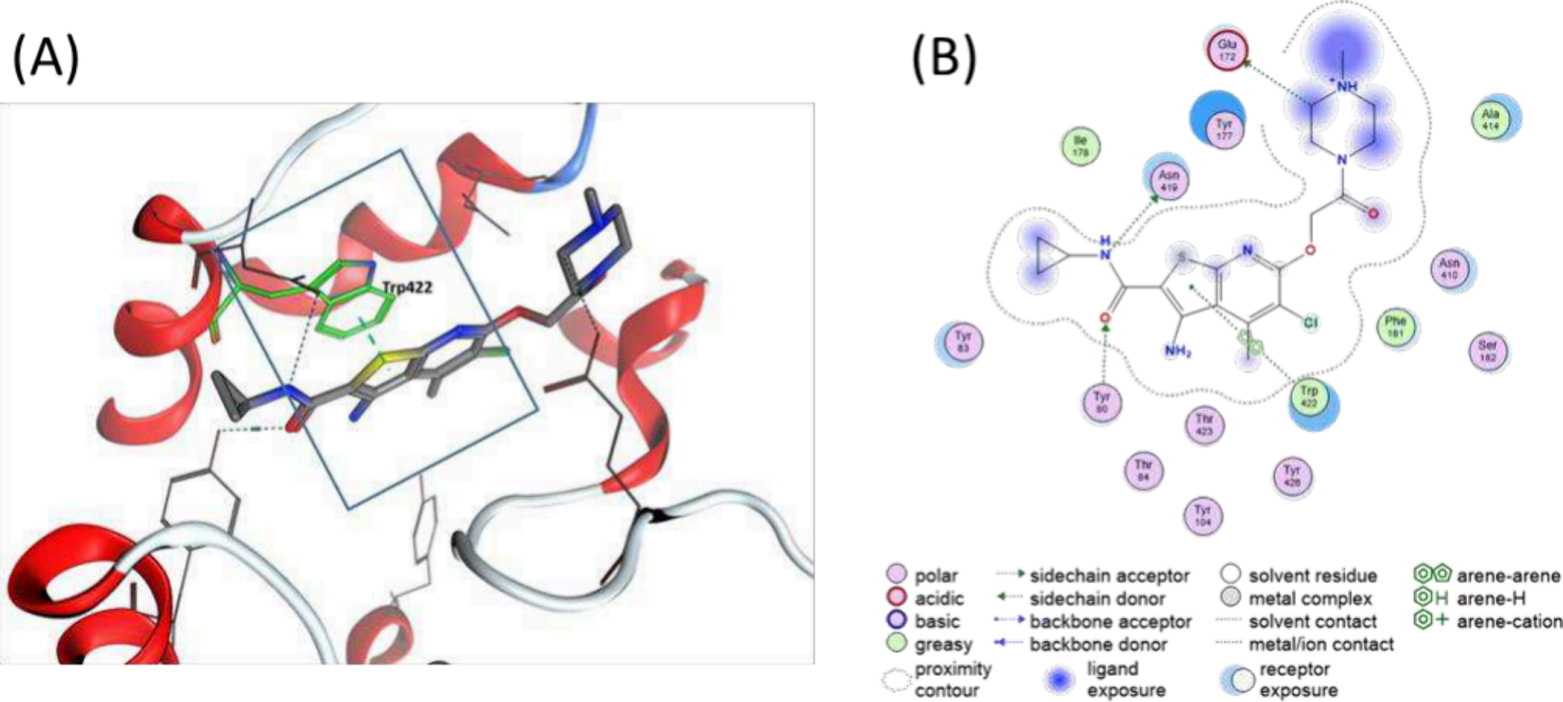
Binding site illustration of **LY2119620** (an M_2_/M_4_ PAM).^26^ (A) The binding mode of **LY2119620** in M_2_ receptor (PDB: 4MQT). **LY2119620** was crystallized in complex with M_2_ receptor along with the orthosteric agonist, iperoxo, for the first time. (B) 2D interaction diagram of **LY2116920**. The π–π interaction has been considered an important driving force for compound binding at the GPCR PAM binding site based on the π–π interaction between Trp422 and 7-aza-benzothiophene motif of **LY2119620**.

While homologation by one carbon with a methylene linker was not tolerated (**17f**, hM_1_ pEC_50_ < 5), **17g** with an sp^3^-rich cyclopentyl ring afforded enhanced activity (rM_1_ pEC_50_ = 6.46 ± 0.03, hM_1_ pEC_50_ = 6.37 ± 0.08) compared to the respective *N*-benzyl and *N*-phenyl counterparts (Table 2, **17f**–**17j**). We were encouraged by this surprising result and overlaid **17g** with **5** and **7** (Figure 4). From the overlay shown in Figure 4, we suspected that the slightly larger cyclohexyl ring might be suboptimal compared to the cyclopentyl ring because the cyclopentyl ring was already slightly larger than the phenyl ring of **17b**. Not surprisingly, **17h** with a cyclohexyl ring was notably weaker compared to **17g** (**17h**, rM_1_ pEC_50_ = 5.83 ± 0.09, hM_1_ pEC_50_ = 5.31 ± 0.12).

**Table 2:**
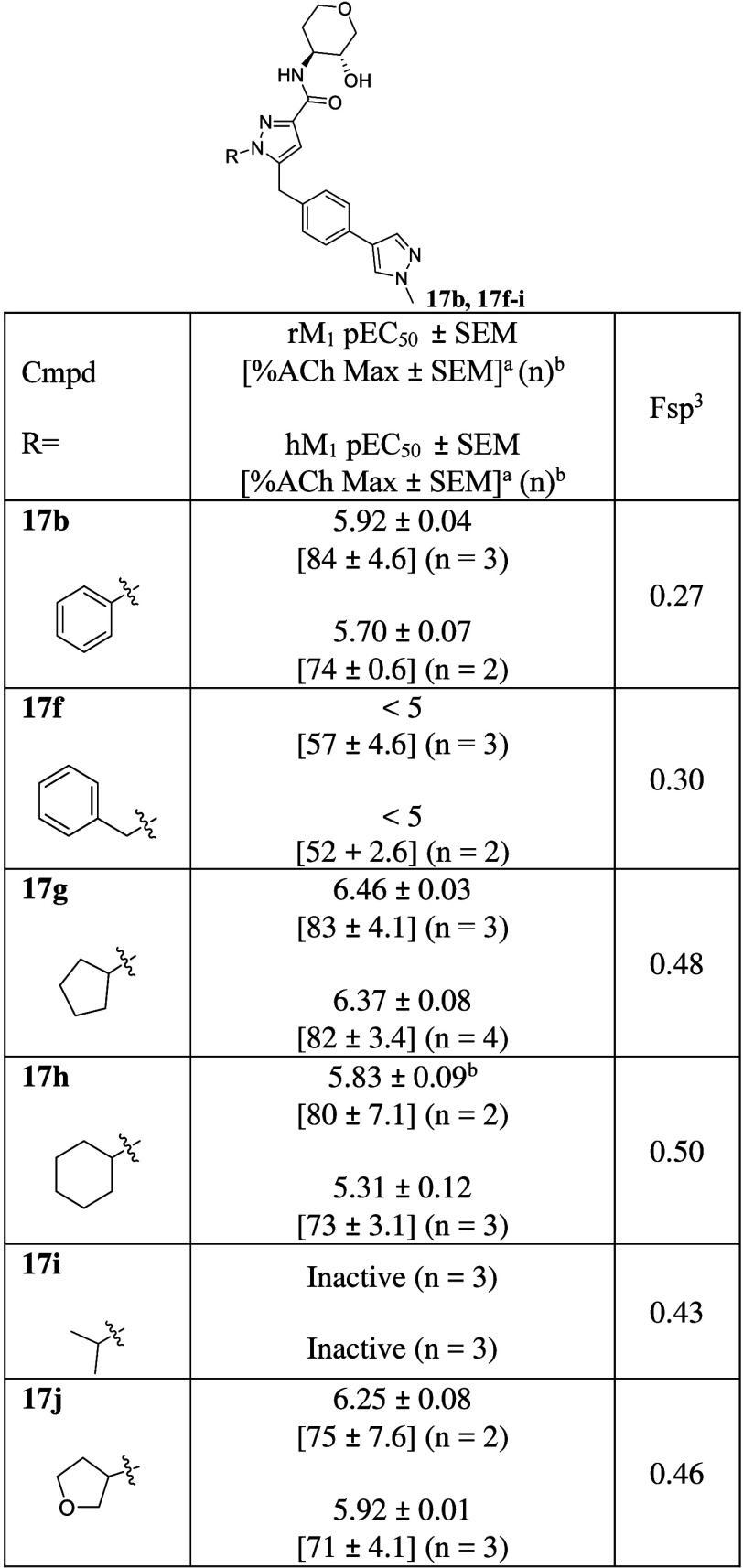
SAR Evaluation of *N*-Substitution of a Pyrazole Core

a Calcium mobilization assays with rM_1_ and hM_1_-CHO cells performed in the presence of an EC_20_ fixed concentration of acetylcholine for PAM activity.

b Each independent experiment was performed in duplicate or triplicate.

**Figure 4:**
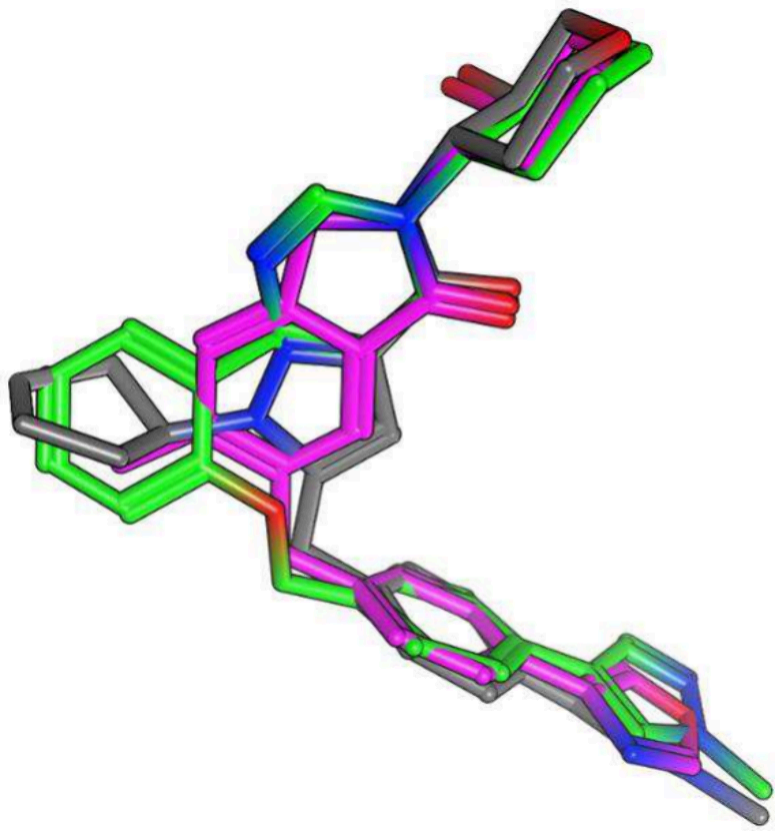
Structural overlay between **5**, **7**, and **17g**. Structures were overlaid via the flexible alignment function using Molecular Operating Environment (MOE).

Based on this SAR trend, we hypothesized that a truncation of the cyclopentyl ring to isopropyl moiety may also be tolerated because the size of the isopropyl moiety may lie between the sizes of phenyl and methyl substituents. However, **17i** with the isopropyl substituent was inactive. Interestingly, our attempt to replace the cyclopentyl ring with a five-membered heterocycle, tetrahydrofuran, was well tolerated (**17j**, rM_1_ pEC_50_ = 6.25 ± 0.08, hM_1_ pEC_50_ = 5.92 ± 0.01), and subsequent efforts such as a chiral resolution were warranted. Additional SAR information will be reported in due course.

Because the binding pose might be changed with a sp^3^-rich substituent, we reinvestigated the amide substitution before shifting our attention to the tail. For second-generation amide libraries with an *N*-cyclopentyl pyrazole core, the amines tolerated in the previous amide scanning exercise (Table 1) were initially focused on (Table 3, **17k**–**17m**). While *N*-cyclopentyl pyrazole analogs consistently showed improved potencies compared to those of *N*-phenyl pyrazole analogs, potency trends were the same ((3*R*,4*S*)-4-aminotetrahydro-2*H*-pyran-3-ol (**17g**) > (1*S*,2*S*)-2-aminocyclohexan-1-ol (**17k**) > (1*S*,2*S*)-2-aminocycloheptan-1-ol (**17l**) > (1*S*,2*S*)-2-aminocyclopentan-1-ol) (**17m**). While there is still room for improvement, **17g** and **17k** showed the best potential with enhanced Fsp^3^ values (hM_1_ pEC_50_ = 6.37 ± 0.08 and 6.23 ± 0.01; Fsp^3^ = 0.48 and 0.50, respectively). In addition, historically less favored amides were also tested to reconfirm the SAR trends (**17n**–**17p**). As expected, **17n**–**17p** showed much weaker potencies compared to the aforementioned preferred amines. Due to the sp^3^-rich character of a cyclopentyl moiety, synthesized analogs tended to have higher Fsp^3^ values (Fsp^3^ = 0.46–0.52) compared to previously reported M_1_ PAMs (Figure 1).

**Table 3:**
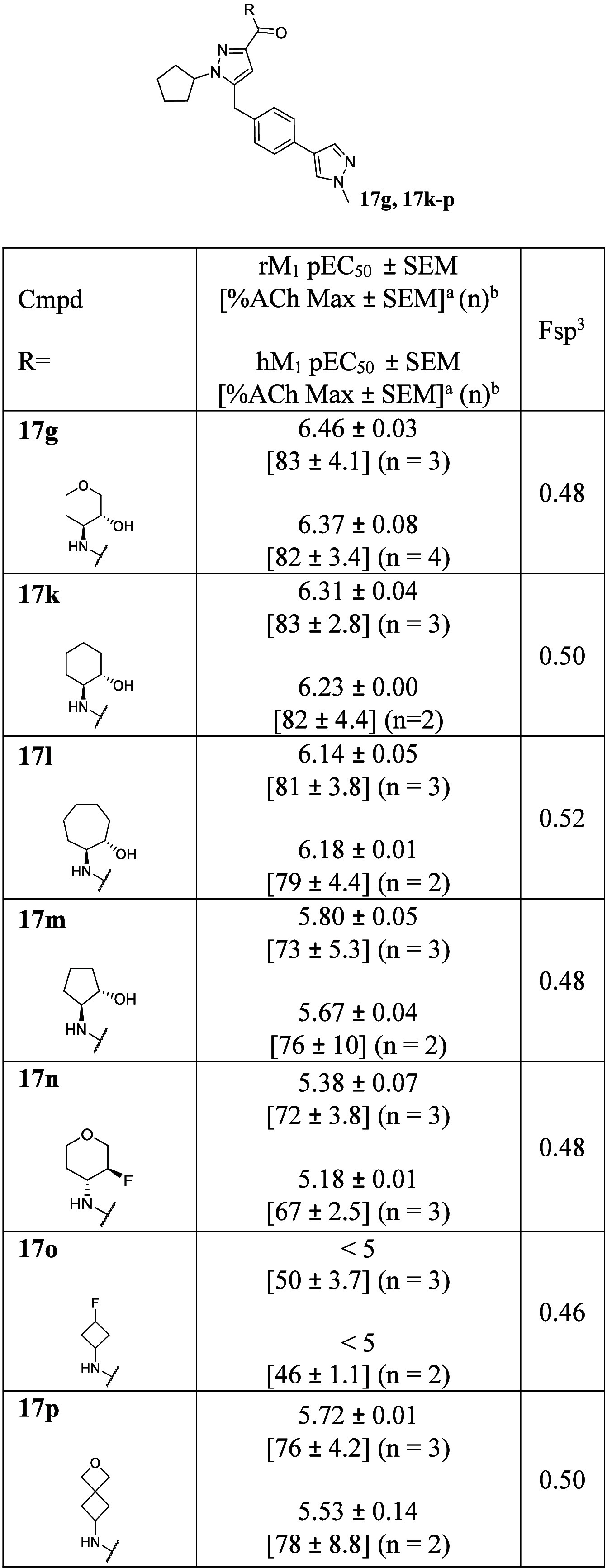
Amide Library Reinvestigation with sp^3^-Rich *N*-Cyclopentyl Pyrazole Core

a Calcium mobilization assays with rM_1_ and hM_1_-CHO cells performed in the presence of an EC_20_ fixed concentration of acetylcholine for PAM activity.

b Each independent experiment was performed in duplicate or triplicate.

Lastly, our SAR interest shifted to the investigation of heterobiaryl tails. As shown in Figure 1, most of the previously highlighted M_1_ PAMs contain heterobiaryl tails. While heterobiaryl moieties are easy to synthesize via Suzuki-Miyaura coupling, the sp^2^-rich character of heterobiaryl moieties is often detrimental to pharmaceutical properties. Therefore, we replaced sp^2^-rich heterobiaryl tails with those containing fewer sp^2^ carbons. Based on the literature review and our SAR knowledge, substituted benzamides were selected as a heterobiaryl replacement. While simple benzamides contain fewer sp^2^ carbons, they are well tolerated in other series (Figure 5A). Additionally, our previous amide libraries indicate no notable shift in the binding pose. Therefore, we anticipated simple benzamides would be tolerated in our novel chemotype as well (Figure 5B). Although detailed SAR discussion is beyond the scope of the current report and will be reported in due course, **21** from the initial library showed encouraging potency (**21**, rM_1_ pEC_50_ = 6.10 ± 0.04, hM_1_ pEC_50_ = 5.90 ± 0.13). In particular, Fsp^3^ values of *N*-cyclopentyl pyrazole analogs were much higher than 0.42 (**21**, Fsp^3^ = 0.52), suggesting the possibility of improved overall pharmaceutical properties.

**Figure 5:**
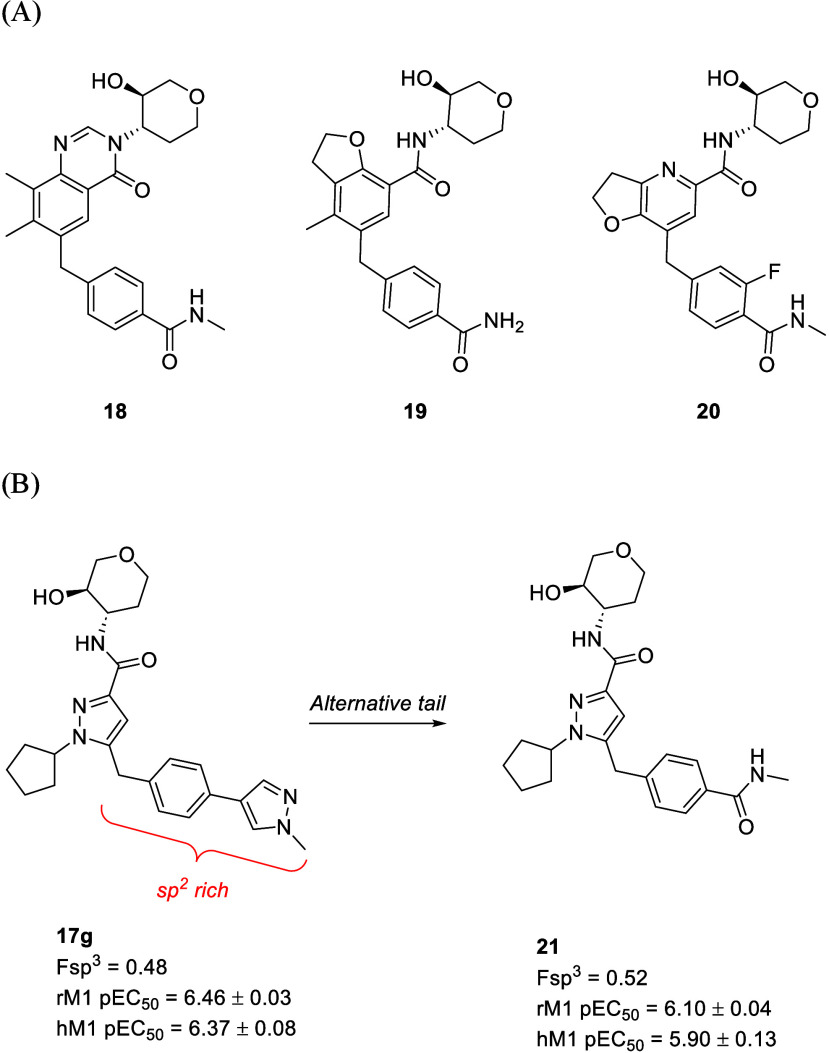
Heterobiaryl tail replacement with substituted benzamides (A) Exemplary compound with benzamide tails **18**,^27^ **19**,^28^ and **20**.^29^ (B) Novel sp^3^-rich M_1_ PAM.

With novel, sp^3^-rich M_1_ PAMs in hand, several compounds were selected for Tier 1 DMPK profiling (Table 4). Selected M_1_ PAMs displayed promising physiochemical properties: MW < 500, TPSA < 100, and cLogP < 5. Notably, aqueous solubilities were improved with higher Fsp.^3^ Rat *in vivo* Tier 1 DMPK profiles from PK/PBL cassette studies also suggested that this chemotype was promising as a new starting point. While **17k** was significantly more CNS penetrant (**17k**, K_p_ = 0.63; **17g**, K_p_ = 0.12), **17g** showed promising aqueous solubilities in both pHs (**17g**, 84.6 μM at pH 2.2, 80.7 μM at pH 6.8).

**Table 4:** Detailed *In Vitro* and *In Vivo* Profiles for Selected M_1_ PAMs^a^

Property	17k	17g
MW	447	449
cLogP	4.61	3.15
TPSA	85	94.2
Fsp^3^	0.50	0.48
Solubility		
pH = 2.2	10.8 μM	84.6 μM
pH = 6.8	<1.12 μM	80.7 μM
Elog*D*_7.4_	4.15	3.05
In Vitro PK		
Rat CL_HEP_ (mL/min/kg)	49.5	32.8
Human CL_HEP_ (mL/min/kg)	14.3	6.7
Human fu_plasma_	0.01	0.08
Rat fu_plasma_	0.03	0.08
Rat fu_brain_	0.01	0.08
In Vivo PK (PK/PBL cassette)		
*t*_1/2_ (h)	0.63	1.21
MRT (h)	0.65	1.28
CL_p_ (mL/min/kg)	56	20.8
V_ss_ (L/kg)	2.2	1.59
AUC (h*ng/mL)	74.4	160
K_p_	0.63	0.12

a In the case of **17k**, *t*_1/2_ was 0.63 h with MRT of 0.65 h. CL_p_ was 56 mL/min/kg, V_ss_ was 2.2 L/kg, and AUC was 74.4 h*ng/mL. Interestingly, slightly more polar **17g** showed lower clearance profiles compared to **17k**. *t*_1/2_ was 1.21 h with MRT of 1.28 h. CL_p_ was 20.8 mL/min/kg, Vss was 1.59 L/kg, and AUC was 160 h*ng/mL.

In conclusion, a series of novel pyrazole-based M_1_ PAMs was discovered from a rationalized scaffold-hopping approach. Pyrazole structures offered unprecedented SAR opportunities and led to the discovery of *N*-cyclopentyl pyrazole as a novel minimum pharmacophore. Sp^3^-rich character positively influenced pharmaceutical properties and offered a promising compound for further optimization. Detailed SAR discussion, along with DMPK profiles within the series, will be reported in a timely manner.

## Supplementary Material



## Notes

General chemistry, experimental information, and syntheses of all other compounds; in vitro and in vivo pharmacology and DMPK methods; supplementary figures and table (PDF)

## Abbreviations

ADMET: absorption, distribution, metabolism, excretion, and toxicity
AUC: area under the curve
CHO: Chinese hamster ovary
CL_p_: plasma clearance
CL_HEP_: hepatic clearance
DMPK: drug metabolism pharmacokinetics
fu_plasma_: fraction unbound in plasma
fu_brain_: fraction unbound in brain
GPCR: G-protein-coupled receptor
HATU: hexafluorophosphate azabenzotriazole tetramethyl uronium
Kp: brain/plasma ratio
MW: molecular weight
MRT: mean residence time
NMO: 4-methylmorpholine *N*-oxide
PBL: plasma:brain level
PAM: positive allosteric modulator
SAR: structure activity relationship
TPAS: topological polar surface area
Vss: volume of distribution at steady state
