# Supplementary material for: Discovery of a Novel sp3‑Rich M1 Positive Allosteric Modulators (PAMs) Chemotype via Scaffold Hopping
Source: ACS Med Chem Lett. 2025 Jun 17;16(7):1231–8. doi: 10.1021/acsmedchemlett.5c00271 (PMC12257413; doi:10.1021/acsmedchemlett.5c00271)
Supplement: Supplementary file 1 [file ml5c00271_si_001.pdf]

Supporting Information

Discovery of a novel sp^3-rich M_1 positive allosteric modulators (PAMs) chemotype via scaffold hopping

Joseph D. Bungard[a,b], Paul Spearing[a,b], Yu Nishio[a,b], Upendra Rathnayake[a,b], Chris C. Presley[a,b], Sichen Chang[a,b], Haley E. Kling[a,b], Analisa D. Thompson[a,b], Hyekyung P. Cho[a,b], Li Peng[a,b], Alice L. Rodriguez[a,b], Colleen M. Niswender[a,b,c,d], Olivier Boutaud[a,b], Valerie Kramlinger[a,b], Carrie K. Jones[a,b], P. Jeffrey Conn[a,b,c], Julie L. Engers[a,b], Darren W. Engers[a,b,]*, Craig W. Lindsley[a,b,d,e,]*, Changho Han[a,b,]*

[a] Warren Center for Neuroscience Drug Discovery, Vanderbilt University, Nashville, TN 37232, USA

[b] Department of Pharmacology, Vanderbilt University School of Medicine, Nashville, Tennessee 37232, USA

[c] Vanderbilt Brain Institute, Vanderbilt University School of Medicine, Nashville, Tennessee 37232, USA

[d] Vanderbilt Kennedy Center, Vanderbilt University Medical Center, Nashville, Tennessee 37232, USA

[e] Department of Chemistry, Vanderbilt University, Nashville, Tennessee 37232, USA

Corresponding authors: Darren W. Engers (darren.engers@Vanderbilt.Edu); Craig W. Lindsley (craig.lindsley@vanderbilt.edu); Changho Han (changho.han@vanderbilt.edu)

TABLE OF CONTENTS

Procedures for Biological Experiments S2

General Methods S8

Exemplary Synthetic Procedure S9

Selected Compound Characterizations S13

Final Compound Characterizations – ^{1}H-NMR, ^{13}C-NMR S25

HRMS data S43

Purity analysis by LC-MS S48

References S53

Procedures for Biological Experiments

Calcium mobilization assays:

To measure the functional activity of positive allosteric modulator (PAM) compounds in a cellular assay, human or rat muscarinic receptor subtype 1 (M_1) was stably expressed in the Chinese hamster ovary (CHO) cells to evoke an increase in intracellular calcium to an EC_{20} concentration of acetylcholine (ACh) agonist. The stable M_1-CHO cells were cultured in F12 medium containing 10% fetal bovine serum, 20 mM HEPES, 100 units/mL antibiotics/antimycotic, 0.5 mg/ml G418. All reagents used were from Life Technologies (Carlsbad, CA) unless otherwise noted.

Briefly, the day before the assay, stable M_1-CHO cells (15,000 cells/20 µL/well) were plated in in black-walled, clear-bottomed, 384 well plates (Greiner Bio-One, Monroe, NC) in the culture medium without G418, and then incubated overnight at 37 °C in the presence of 5% CO_2. The next day, calcium assay buffer (Hank's balanced salt solution (HBSS), 20 mM HEPES, 2.5 mM Probenecid, 4.16 mM sodium bicarbonate (Sigma-Aldrich, St. Louis, MO) was prepared to dilute compounds, agonists, and Fluo-4-acetomethoxyester (Fluo-4-AM, Ion Biosciences), fluorescent calcium indicator dye. Compounds were serially diluted 1:3 into 10 point concentration response curves in DMSO using the Bravo Liquid Handler (Agilent, Santa Clara, CA), transferred to a 384 well daughter plates using an Echo acoustic liquid handler (Beckman Coulter, Indianapolis, Indiana), and diluted in assay Buffer to a 2X final concentration. The agonist plates were prepared using acetylcholine (ACh, Sigma-Aldrich, St. Louis, MO) concentrations for the EC_{20} and EC_{MAX} responses by diluting in assay buffer to a 5X final concentration. The 2X dye solution (2.3 µM) was prepared by mixing a 2.3 mM Fluo-4-AM stock in DMSO with 10% (w/v) pluronic acid F-127 in a 1:1 ratio in assay buffer. Using a microplate washer (BioTek, Winooski, VT), cells were washed with assay buffer for 3 times to remove medium. After the final wash, 20 µL of assay buffer remained in the cell plates. Immediately, 20 µL of the 2X dye solution (final 1.15 µM) was added to each well of the cell plate using a Multidrop Combi dispenser (Thermo Fisher, Waltham, MA). After cells were incubated with the dye solutions for 45 min at 37 °C in the presence of 5% CO_2, the dye solutions were removed and replaced with assay buffer using a microplate washer, leaving 20 µL of assay buffer in the cell plate. The compound, agonist, and cell plates were placed inside the Functional Drug Screening System uCell (FDSS uCell, Hamamatsu, Japan) to measure the calcium flux. The triple add protocol was used to measure Ca kinetics; Compound, ACh for EC_{20}, and ACh for EC_{MAX} adds in an order. Briefly, after establishment of a fluorescence baseline

for 2 seconds (excitation, 480 nm; emission, 530 nm), first add occurred by adding 20 μL of test compound to the cells, and the response was measured for 140 seconds. This is followed by second add; 10 μL (5X) of an EC_{20} concentration of ACh agonist was added to the cells, and the response of the cells was measured for 125 seconds. Immediately, the third add occurred by adding 12 ul assay buffer or 5X of an EC_{max} concentration of ACh and the response of the cells was measured for 90 seconds. Assay buffer was added to the wells that incubated with compounds followed by EC_{20} concentration of ACh. Agonists containing EC_{max} concentration of ACh were added to the wells that incubated with DMSO and assay buffer from the 1st and 2nd adds to measure maximum Ca responses (EC_{max}). Calcium fluorescence was recorded as fold over basal fluorescence and raw data were normalized to the maximal response to ACh agonist. Potency (EC_{50}) and maximum response (% ACh Max) for compounds was determined using a four-parameter logistical equation using GraphPad Prism (La Jolla, CA) or the Dotmatics software platform (Woburn, MA) :

$$y = bottom + \frac{top - bottom}{1 + 10^{(LogEC50 - A)Hillslope}}$$

where *A* is the molar concentration of the compound; *bottom* and *top* denote the lower and upper plateaus of the concentration-response curve; HillSlope is the Hill coefficient that describes the steepness of the curve; and EC_{50} is the molar concentration of compound required to generate a response halfway between the *top* and *bottom*.

Plasma protein binding and Brain homogenate binding:

The protein binding of each compound was determined in plasma via equilibrium dialysis employing rapid equilibrium dialysis (RED) plates (ThermoFisher Scientific, Rochester, NY). Plasma was added to the 96 well plate containing test compound and mixed thoroughly for a final concentration of 5 μM. Subsequently, an aliquot of the plasma-compound mixture was transferred to the *cis* chamber (red) of the RED plate, with a phosphate buffer (25 mM, pH 7.4) in the *trans* chamber. The RED plate was sealed and incubated for 6 hours at 37 °C with shaking (120 rpm). At completion, aliquots from each chamber were transferred to a new 96 well plate and were diluted 1:1 with either plasma (*trans*) or buffer (*cis*), at which time ice-cold acetonitrile containing internal standard (50 nM carbamazepine) (3 volumes) was added to extract the matrices. The plate was centrifuged (3000 RCF, 10 min) and supernatants were transferred and diluted 1:1 (supernatant : water) into a new 96 well plate, which was then sealed in preparation for LC/MS/MS analysis. Each compound was assayed in triplicate within the same 96-well plate.

A similar approach was used to determine the degree of brain homogenate binding, which employed the same methodology and procedure with the following modifications: 1) a final compound concentration of 1 µM was used, 2) naïve rat brains were homogenized in DPBS (1:3 composition of brain: DPBS, w/w) using a Mini-Bead Beater™ machine in order to obtain brain homogenate, which was then treated in the same manner as the plasma samples in the previously described plasma protein binding assay.

A similar approach was used to determine the degree of brain homogenate binding, which employed the same methodology and procedure with the following modifications: 1) a final compound concentration of 1 µM was used, 2) naïve rat brains were homogenized in DPBS (1:3 composition of brain: DPBS, w/w) using a Mini-Bead Beater™ machine in order to obtain brain homogenate, which was then treated in the same manner as the plasma samples in the previously described plasma protein binding assay. Fraction unbound for both plasma and brain samples was determined using Equation 4.

$$f_u = \frac{Conc_{buffer}}{Conc_{plasma}}$$

Equation 4 Determination of fraction unbound in plasma.

The diluted fraction unbound (f_u2) in brain was calculated in the same manner by using brain homogenate rather than plasma. Undiluted fraction unbound for the brain was calculated using Equation 5

$$f_u = \frac{1/4}{\left\{\left(\frac{1}{fu2}\right) - 1\right\} + 1/4}$$

Equation 5 Determination of fraction unbound in brain. F_u2 represents the diluted fraction unbound.

Intrinsic clearance:

Human or rat hepatic microsomes (0.5 mg/mL) and 1 µM test compound were incubated in 100 mM potassium phosphate pH 7.4 buffer with 3 mM $MgCl_2$ at 37 °C with constant shaking. After a 5 min preincubation, the reaction was initiated by the addition of NADPH (1 mM). At selected time intervals (0, 3, 7, 15, 25, and 45 min), aliquots were taken and subsequently placed into a 96-well plate containing cold acetonitrile with internal standard (50 ng/mL carbamazepine). Plates were then centrifuged at 3000 rcf (4 °C) for 10 min, and the supernatant was transferred to a separate 96-well plate and diluted 1:1 with water for LC/MS/MS analysis. The *in vitro* half-life ($_{1/2}$, min, Eq. 1), intrinsic clearance (CL_{int}, mL/min/kg, Eq. 2), and subsequent predicted hepatic clearance (CL_{hep}, mL/min/kg, Eq. 3) was determined employing the following equations:

(1) $T_{1/2} = \frac{Ln(2)}{K}$

where k represents the slope from linear regression analysis of the natural log percent remaining of a test compound as a function of incubation time

(2) $CL_{int} = \frac{0.693}{in\ vitro T_{1/2}}\ x\ \frac{mL\ incubation}{mg\ microsomes}\ x\ \frac{45\ mg\ microsomes}{gram\ liver}\ x\ \frac{20^{a}\ gram\ liver}{kg\ body\ wt}$

[a]scale-up factors: of 20 (human) or 45 (rat)

(3) $CL_{hep} = \frac{Q_h \cdot CL_{int}}{Q_h + CL_{int}}$

where Q_h (hepatic blood flow, mL/min/kg) is 21 (human) or 70 (rat).

LC/MS/MS Bioanalysis of Samples from Plasma Protein Binding and Intrinsic Clearance Assays:

Samples were analyzed on a Thermo Electron TSQ Quantum Ultra triple quad mass spectrometer (San Jose, CA) via electrospray ionization (ESI) with two Themo Electron Accella pumps (San Jose, CA), and a Leap Technologies CTC PAL autosampler (Carrboro, NC). Analytes were separated by gradient elution on a dual column system with two Thermo Hypersil Gold (2.1 x 30 mm, 1.9 µm) columns (San Jose, CA) thermostated at 40 °C. HPLC mobile phase A was 0.1% formic acid in water and mobile phase B was 0.1% formic acid in acetonitrile. The gradient started at 10% B after a 0.2 min hold and was linearly increased to 95% B over 0.8 min; hold at 95% B for 0.2 min; returned to 10% B in 0.1 min. The total run time was 1.3 min and the HPLC flow rate was 0.8 mL/min. While pump 1 ran the gradient method, pump 2 equilibrated the alternate column isocratically at 10% B. Compound optimization, data collection, and processing was performed using Thermo Electron's QuickQuan software (v2.3) and Xcalibur (v2.0.7 SP1).

In vivo DMPK experimental:

Determination of brain to plasma ratio:

Sample Analysis: Concentrations in plasma and brain homogenates were quantified by liquid chromatography tandem mass spectrometry (LC-MS/MS). Whole brains were homogenized in 3 mL of 70:30 IPA:water in a mini bead beater for 3 min, and centrifuged at 3,500 g for 5 min. 5 uL of the supernatant was diluted in 15 uL of blank plasma for quantification of the analytes. Plasma samples were centrifuged at 3,500 g for 5 min. A standard curve was generated by diluting the analytes DMSO stocks with blank plasma to obtain a final concentration of 10,000 ng/ml followed by a serial dilution down to 0.5 ng/ml. Quality controls were generated by a serial dilution of the 5,000 ng/ml standard curve solution in blank plasma to obtain 3 concentrations of 500, 50, and 5 ng/ml. 20 uL of brain diluted in plasma, plasma, blank plasma, standard curve and QC samples were loaded in a V-bottom 96-well plate. 120 uL of acetonitrile containing 0.05 uM carbamazepine (internal standard) was added to each well and the plate was centrifuged at 3,500 g for 5 min. 60 uL of the supernatant of each well (protein free) was transferred to a new 96-well plate containing 60 uL of water. The plates were sealed for analysis by LC-MS/MS.

Plasma and brain tissue samples originating from in vivo studies were analyzed by electrospray ionization using an AB Sciex Q-TRAP 5500 (Foster City, CA) that was coupled to a Shimadzu LC-20AD pump (Columbia, MD) and a Leap Technologies CTC PAL auto-sampler (Carrboro, NC). Analytes were separated by gradient elution using a C18 column (3 x 50 mm, 3 mm; Fortis Technologies Ltd, Cheshire, UK) that was thermostated at 40 °C. HPLC mobile phase A was 0.1% formic acid in water (pH unadjusted); mobile phase B was 0.1% formic acid in acetonitrile (pH unadjusted). A 10% B gradient was held for 0.2 min and was linearly increased to 90% B over 0.8 min, with an isocratic hold for 0.5 min, before transitioning to 10% B over 0.05 min. The column was re-equilibrated (1 min) before the next sample injection. The total run time was 2.55 min, and the HPLC flow rate was 0.5 ml/min. The source temperature was set at 500 °C, and mass spectral analyses were performed using a Turbo-Ion spray source in positive ionization mode (5.0-kV spray voltage) and using multiple-reaction monitoring of transitions specific for the analytes. All data were analyzed using AB Sciex Analyst 1.5.1 software.

Brain plasma concentration ratio (K_p) was calculated by dividing brain concentration by plasma concentration for each animal. Unbound brain to unbound plasma concentration ratio ($K_{p,uu}$) is calculated using the following formula: $K_{p,uu}$ = (Brain ng/g x brain fu)/(plasma ng/ml x plasma fu).

Animal care and use:

All animal study procedures were approved by the Institutional Animal Care and Use Committee and were conducted in accordance with the National Institutes of Health regulations of animal care covered in Principles of Laboratory Animal Care (National Institutes of Health).

General Methods

All reactions were carried out employing standard chemical techniques under inert atmosphere. Solvents used for extraction, washing, and chromatography were reagent grade. All reagents were purchased from commercial sources and were used without further purification. All microwave reactions were carried out in sealed tubes in a Biotage Initiator microwave synthesis reactor. Temperature control was automated via IR sensor and all indicated temperatures correspond to the maximal temperature reached during each experiment. Analytical HPLC was performed on an Agilent 1200 LCMS with UV detection at 215 nm and 254 nm along with ELSD detection and electrospray ionization, with all final compounds showing > 95% purity and a parent mass ion consistent with the desired structure. All NMR spectra were recorded on a 400 MHz Brüker AV-400 instrument. ^{1}H chemical shifts are reported as δ values in ppm relative to the residual solvent peak ($CDCl_3$ = 7.26, CD_3OD = 3.31). Data are reported as follows: chemical shift, multiplicity (br. = broad, s = singlet, d = doublet, t = triplet, q = quartet, dd = doublet of doublets, m = multiplet), coupling constant (Hz), and integration. ^{13}C chemical shifts are reported as δ values in ppm relative to the residual solvent peak ($CDCl_3$ = 77.16). High resolution mass spectra were obtained on an Agilent 6540 UHD Q-TOF with ESI source. Automated flash column chromatography was performed on a Teledyne ISCO Combiflash Rf system. For compounds that were purified on a Gilson preparative reversed-phase HPLC, the system comprised of a 333 aqueous pump with solvent-selection valve, 334 organic pump, GX-271 or GX-281 liquid hander, two column switching valves, and a 155 UV detector. UV wavelength for fraction collection was user-defined, with absorbance at 254 nm always monitored. Method: Phenomenex Axia-packed Luna C18, 30 x 50 mm, 5 µm column. Mobile phase: CH_3CN in H_2O (0.1% TFA). Gradient conditions A: 0.75 min equilibration, followed by user defined gradient (starting organic percentage, ending organic percentage, duration), hold at 95% CH_3CN in H_2O (0.1% TFA) for 1 min, 50 mL/min, 23 ºC. Gradient conditions B: 0.75 min equilibration, followed by user defined gradient (starting organic percentage, ending organic percentage, duration), hold at 95% CH_3CN in H_2O (0.05% NH_4OH for 1 min, 50 mL/min, 23 ºC. cLogP was calculated using PerkinElmer ChemDraw professional version 20.1.0.110. Fsp^3 was calculated using SwissADME.[1] No unexpected or unusually high safety hazards were encountered.

Exemplary Synthetic Procedure (Compound 17g)

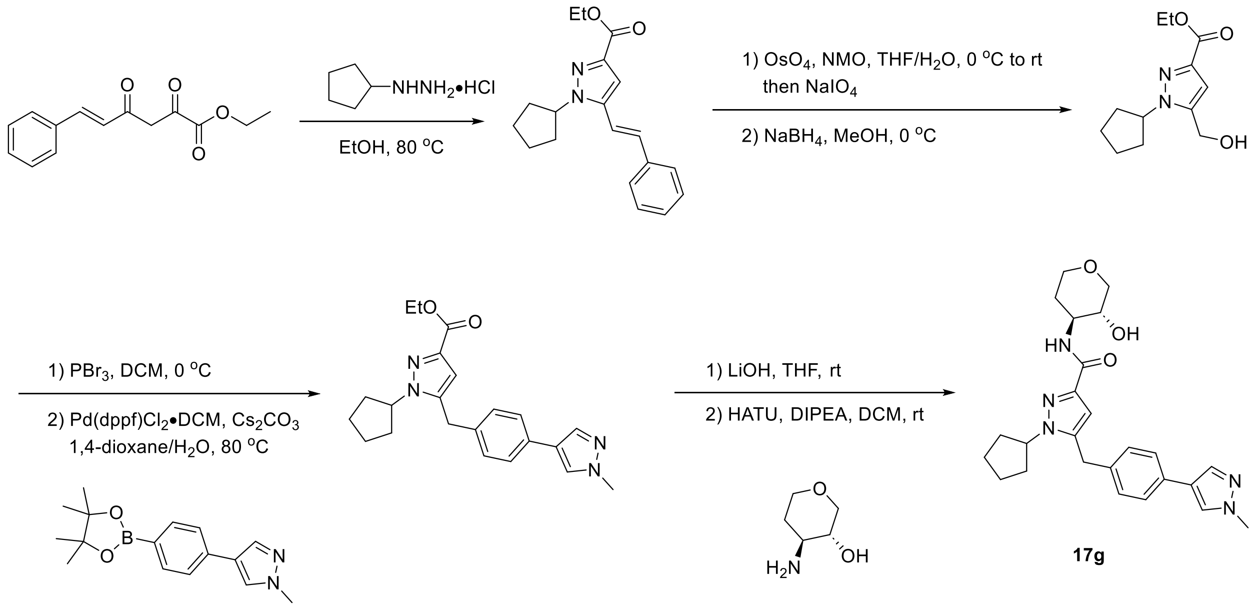

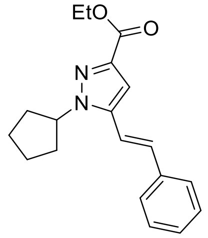

Step A. Ethyl (*E*)-1-cyclopentyl-5-styryl-1*H*-pyrazole-3-carboxylate

To a solution of ethyl *(E)*-2,4-dioxo-6-phenyl-hex-5-enoate (1.0 g, 4.06 mmol) in EtOH (16 mL) was added cyclopentylhydrazine hydrochloride (832 mg, 6.09 mmol) and stirred at 80 °C. After 2 hours, the reaction mixture was cooled to RT and diluted with EtOAc (20 mL). The resulting mixture was washed with sat. aq. $NaHCO_3$ and brine. Organics were then dried over Na_2SO_4, filtered, and concentrated under reduced pressure. The crude product was purified *via* silica gel chromatography (Gradient: 0 to 40% EtOAc in hexanes) to provide ethyl *(E)*-1-cyclopentyl-5-styryl-1*H*-pyrazole-3-carboxylate (1.08 g, 86% yield) as a yellow solid. LCMS ES-MS *m/z* 311.1 $[M+H]^+$. ^1H NMR (400 MHz, $CDCl_3$) δ 7.52 – 7.47 (m, 2H), 7.38 (t, *J* = 7.4 Hz, 2H), 7.34 – 7.29 (m, 1H), 7.08 (d, *J* = 16.1 Hz, 1H), 7.02 – 6.95 (m, 2H), 4.80 (p, *J* = 7.7 Hz, 1H), 4.40 (q, *J* = 7.1 Hz, 2H), 2.29 – 2.18 (m, 2H), 2.18 – 2.08 (m, 2H), 2.03 – 1.91 (m, 2H), 1.76 – 1.64 (m, 2H), 1.40 (t, *J* = 7.1 Hz, 3H).

Caution! *Cyclopentylhydrazine hydrochloride is classified as a GHS07, skin and eye irritant, category 2 and skin sensitization, category 1. Personal protective equipment must be always worn while handling the chemical to avoid any skin or eye contact.*

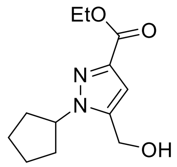

Step B and C. Ethyl 1-cyclopentyl-5-(hydroxymethyl)-1*H*-pyrazole-3-carboxylate

A solution of ethyl *(E)*-1-cyclopentyl-5-styryl-1*H*-pyrazole-3-carboxylate (3.21 g, 10.3 mmol) in THF (39 mL) and water (10 mL) was cooled to 0 °C, and osmium tetroxide (2.5 wt. % in *tert*-butanol, 6.48 mL, 0.52 mmol) and 4-methylmorpholine *N*-oxide (1.82 g, 15.5 mmol) were added sequentially. The resulting solution was stirred at 0 °C for 15 minutes and at RT overnight. After the complete conversion of the starting material to the corresponding diol, $NaIO_4$ (5.55 g, 25.9 mmol) was added and stirred at RT for 2 hours. The reaction was quenched by adding a solution of sat. aq. $Na_2S_2O_3$ and further stirred for 5 min. The organic layer was separated, and the aqueous layer was extracted with DCM (3 x 50 mL). The combined organic layer was washed with brine, dried over Na_2SO_4, and concentrated under reduced pressure to afford the corresponding aldehyde as a crude.

The aldehyde was dissolved in MeOH (48 mL) and cooled to 0 °C in an ice bath. To this solution, $NaBH_4$ (587 mg, 15.5 mmol) was added slowly and stirred at 0 °C for 1.5 hours. After the complete consumption of the starting material, the reaction was diluted with DCM and quenched with sat. NH_4Cl solution. The organic layer was separated, and the aqueous layer was extracted with DCM (3 x 20 mL). The combined organic layer was washed with brine, dried over Na_2SO_4, and concentrated under reduced pressure. The crude product was purified *via* silica gel chromatography (Gradient: 0 to 70% EtOAc in hexanes) to provide ethyl 1-cyclopentyl-5-(hydroxymethyl)-1*H*-pyrazole-3-carboxylate (2.47 g, quantitative yield over 2 steps) as a colorless oil. LCMS ES-MS *m/z* 239.2 $[M+H]^+$. ^1H NMR (400 MHz, $CDCl_3$) δ 6.60 (s, 1H), 4.76 (p, *J* = 7.9 Hz, 1H), 4.65 (s, 2H), 4.32 (q, *J* = 7.1 Hz, 2H), 2.74 (s, 1H), 2.17 – 2.00 (m, 4H), 1.98 – 1.83 (m, 2H), 1.70 – 1.55 (m, 2H), 1.34 (t, *J* = 7.1 Hz, 3H).

Caution! *Osmium tetroxide is a strong oxidizing agent and extremely toxic. It is classified as a GHS02,GHS06,GHS08 and GHS05 toxin. As given in the procedure, the reagent should be used as a solution of 2.5 wt.% in tert-butanol to avoid any acute exposure and personal protective equipment must be worn at all times, while handling the chemical.*

Caution! *4-methylmorpholine N-oxide is an oxidant and classified a flammable solid, categories 1,2. It is also a carcinogenic reproductive toxin. Care should be taken to avoid any skin and respiratory contact.*

Caution! *Sodium periodate is a strong oxidant and a toxic substance. It is also hazardous to the aquatic environment and care should be taken to properly quench it before discarding.*

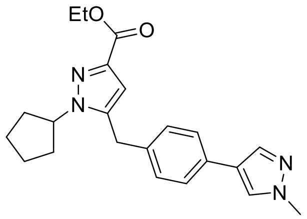

Step C and D. Ethyl 1-cyclopentyl-5-(4-(1-methyl-1*H*-pyrazol-4-yl)benzyl)-1*H*-pyrazole-3-carboxylate

To a solution of ethyl 1-cyclopentyl-5-(hydroxymethyl)-1*H*-pyrazole-3-carboxylate (950 mg, 3.99 mmol) in DCM (20 mL) at 0 °C was added PBr_3 (0.56 mL, 5.98 mmol) slowly and the resulting mixture stirred at 0 °C until the starting material was completely consumed. Upon completion, the reaction was quenched with sat. aq. $NaHCO_3$. The organic layer was separated, and the aqueous layer was extracted with DCM (3 x 50 mL). The combined organic layer was washed with brine, dried over $MgSO_4$, and concentrated under reduced pressure to afford ethyl 5-(bromomethyl)-1-cyclopentyl-1*H*-pyrazole-3-carboxylate as a crude (1.201 g), which was taken to the next step without further purification.

To a solution of ethyl 5-(bromomethyl)-1-cyclopentyl-1*H*-pyrazole-3-carboxylate (252.8 mg, 0.84 mmol) in 1,4-dioxane (1.5 mL) and water (1 mL) was added a solution of 1-methyl-4-(4-(4,4,5,5-tetramethyl-1,3,2-dioxaborolan-2-yl)phenyl)-1*H*-pyrazole (459 mg, 1.07 mmol) in 1,4-dioxane (1.5 mL), Cs_2CO_3 (826 mg, 2.52 mmol), and Pd(dppf)Cl_2•DCM (137.4 mg, 0.17 mmol). The resulting mixture was stirred at 80 ^oC until the starting material was completely consumed. Upon completion, the mixture was cooled to RT, diluted with EtOAc, filtered through Celite, and concentrated under reduced pressure. The crude residue was purified *via* reversed-phase HPLC

(25-90% MeCN/Water contains 0.05% NH_4OH) to provide ethyl 1-cyclopentyl-5-(4-(1-methyl-1*H*-pyrazol-4-yl)benzyl)-1*H*-pyrazole-3-carboxylate (150.7 mg, 47% yield). LCMS ES-MS *m/z* 379.2 $[M+H]^+$. ^1H NMR (400 MHz, $CDCl_3$) δ 7.74 (d, *J* = 0.8 Hz, 1H), 7.60 (s, 1H), 7.41 (d, *J* = 8.2 Hz, 2H), 7.12 (d, *J* = 8.3 Hz, 2H), 6.53 (s, 1H), 4.50 (p, *J* = 7.7 Hz, 1H), 4.37 (q, *J* = 7.1 Hz, 2H), 4.01 (s, 2H), 3.94 (s, 3H), 2.15 – 2.02 (m, 2H), 1.98 – 1.83 (m, 4H), 1.64 – 1.51 (m, 2H), 1.36 (t, *J* = 7.1 Hz, 3H).

Caution! *Phosphorus tribromide is a corrosive chemical and a toxic substance. It is a skin irritant, and can cause serious eye damage. Personal protective equipment must be always worn to prevent any skin and eye contact.*

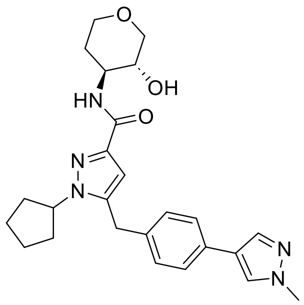

Step E and F. 1-Cyclopentyl-*N*-((3*R*,4*S*)-3-hydroxytetrahydro-2*H*-pyran-4-yl)-5-(4-(1-methyl-1*H*-pyrazol-4-yl)benzyl)-1*H*-pyrazole-3-carboxamide (17g)

To a solution of ethyl 1-cyclopentyl-5-[[4-(1-methylpyrazol-4-yl)phenyl]methyl]pyrazole-3-carboxylate (150.7 mg, 0.4 mmol) in THF (1.5 mL) and MeOH (0.5 mL) was added a solution of LiOH (1 M, 0.48 mL, 0.48 mmol). The resulting mixture was stirred at RT until the starting material was completely consumed. Upon completion, the reaction was concentrated to remove MeOH, acidified with 2N HCl until the pH reached 3-4, and further concentrated to dryness to afford 1-cyclopentyl-5-(4-(1-methyl-1*H*-pyrazol-4-yl)benzyl)-1*H*-pyrazole-3-carboxylic acid (156 mg, 0.40 mmol) as a crude.

To a solution of 1-cyclopentyl-5-(4-(1-methyl-1*H*-pyrazol-4-yl)benzyl)-1*H*-pyrazole-3-carboxylic acid (14.3 mg, 0.04 mmol) in DMF (0.5 mL) was added HATU (29.2 mg, 0.08 mmol) followed by *N*,*N*-diisopropylethylamine (0.02 mL, 0.11 mmol). After stirring for 20 mins, *(3R,4S)*-4-aminotetrahydro-2*H*-pyran-3-ol (8.9 mg, 0.08 mmol) was added and further stirred for 2 h. Upon completion of amide formation, the crude mixture was filtered and purified *via* reversed-phase HPLC (30 - 90% MeCN/Water contains 0.05% NH_4OH) to produce 1-cyclopentyl-*N*-

((3*R*,4*S*)-3-hydroxytetrahydro-2*H*-pyran-4-yl)-5-(4-(1-methyl-1*H*-pyrazol-4-yl)benzyl)-1*H*-pyrazole-3-carboxamide (1.9 mg, 12% yield) as a clear film. HRMS m/z calcd for $C_{25}H_{31}N_5O_3$ $[M+H]^+$ 450.2500; found 450.2504. ^{1}H NMR (400 MHz, $CDCl_3$) δ 7.74 (d, *J* = 0.8 Hz, 1H), 7.59 (d, *J* = 0.8 Hz, 1H), 7.40 (d, *J* = 8.2 Hz, 2H), 7.11 (d, *J* = 8.2 Hz, 2H), 6.91 (d, *J* = 6.3 Hz, 1H), 6.56 (s, 1H), 4.50 (p, *J* = 7.2 Hz, 1H), 4.07 (ddd, *J* = 11.4, 5.1, 1.1 Hz, 1H), 4.03 – 3.85 (m, 7H), 3.59 (td, *J* = 9.6, 5.0 Hz, 1H), 3.44 (td, *J* = 11.9, 2.2 Hz, 1H), 3.19 (dd, *J* = 11.4, 10.0 Hz, 1H), 2.05 – 1.82 (m, 7H), 1.82 – 1.69 (m, 1H), 1.68 – 1.53 (m, 2H). *OH proton is not observable. ^{13}C NMR (101 MHz, $CDCl_3$) δ 164.8, 144.1, 143.1, 136.8, 135.2, 131.5, 129.0, 127.0, 126.0, 122.9, 107.2, 72.5, 71.5, 66.5, 59.9, 54.0, 39.2, 33.0, 32.9, 31.7, 31.2, 24.7.

Advanced Intermediates

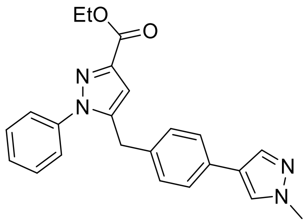

Intermediate A. Ethyl 5-(4-(1-methyl-1*H*-pyrazol-4-yl)benzyl)-1-phenyl-1*H*-pyrazole-3-carboxylate.

Intermediate A was synthesized according to exemplary synthetic procedure. (53.6 mg, 28% yield over two steps). LCMS ES-MS *m/z* 387.2 $[M+H]^+$. ^{1}H NMR (400 MHz, $CDCl_3$) δ 7.73 (s, 1H), 7.58 (s, 1H), 7.47 – 7.41 (m, 3H), 7.41 – 7.35 (m, 4H), 7.05 (d, *J* = 8.1 Hz, 2H), 6.70 (s, 1H), 4.41 (q, *J* = 7.1 Hz, 2H), 3.96 (s, 2H), 3.94 (s, 3H), 1.38 (t, *J* = 7.1 Hz, 3H).

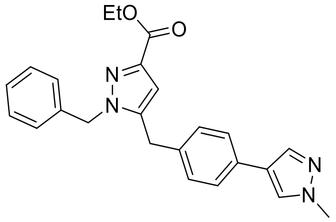

Intermediate B. Ethyl 1-benzyl-5-(4-(1-methyl-1*H*-pyrazol-4-yl)benzyl)-1*H*-pyrazole-3-carboxylate

Intermediate B was synthesized according to exemplary synthetic procedure. (40.6 mg, 21% yield over two steps). LCMS ES-MS *m/z* 401.2 $[M+H]^+$. ^{1}H NMR (400 MHz, $CDCl_3$) δ 7.73 (s, 1H),

7.58 (s, 1H), 7.37 (d, *J* = 8.0 Hz, 2H), 7.34 – 7.25 (m, 3H), 7.09 – 7.04 (m, 2H), 7.02 (d, *J* = 8.0 Hz, 2H), 6.60 (s, 1H), 5.31 (s, 2H), 4.40 (q, *J* = 7.1 Hz, 2H), 3.94 (s, 3H), 3.80 (s, 2H), 1.39 (t, *J* = 7.1 Hz, 3H).

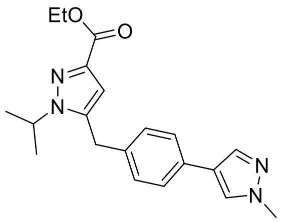

Intermediate C. Ethyl 1-isopropyl-5-(4-(1-methyl-1*H*-pyrazol-4-yl)benzyl)-1*H*-pyrazole-3-carboxylate

Intermediate C was synthesized according to exemplary synthetic procedure. (44.3 mg, 26% yield over two steps). LCMS ES-MS *m/z* 353.2 $[M+H]^+$. ^{1}H NMR (400 MHz, $CDCl_3$) δ 7.74 (d, *J* = 0.8 Hz, 1H), 7.60 (d, *J* = 0.8 Hz, 1H), 7.41 (d, *J* = 8.2 Hz, 2H), 7.12 (d, *J* = 8.5 Hz, 2H), 6.54 (s, 1H), 4.48 – 4.33 (m, 3H), 4.00 (s, 2H), 3.94 (s, 3H), 1.42 (d, *J* = 6.7 Hz, 6H), 1.37 (t, *J* = 7.1 Hz, 3H).

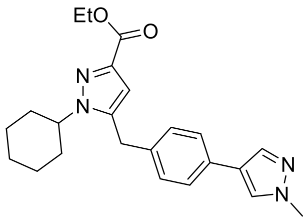

Intermediate D. Ethyl 1-cyclohexyl-5-(4-(1-methyl-1*H*-pyrazol-4-yl)benzyl)-1*H*-pyrazole-3-carboxylate

Intermediate D was synthesized according to exemplary synthetic procedure (62 mg, 55% yield over two steps) LCMS ES-MS *m/z* 393.5 $[M+H]^+$. ^{1}H NMR (400 MHz, $CDCl_3$) δ 7.74 (d, *J* = 0.8 Hz, 1H), 7.60 (d, *J* = 0.8 Hz, 1H), 7.45 – 7.37 (m, 2H), 7.18 – 7.10 (m, 2H), 6.52 (s, 1H), 4.37 (q, *J* = 7.1 Hz, 2H), 4.03 – 3.96 (m, 3H), 3.94 (s, 3H), 2.08 – 1.93 (m, 2H), 1.87 – 1.79 (m, 2H), 1.78 – 1.70 (m, 2H), 1.68 – 1.62 (m, 1H), 1.36 (t, *J* = 7.1 Hz, 3H), 1.30 – 1.19 (m, 3H).

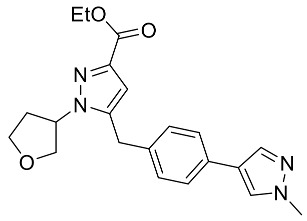

Intermediate E. Ethyl 5-(4-(1-methyl-1*H*-pyrazol-4-yl)benzyl)-1-(tetrahydrofuran-3-yl)-4,5-dihydro-1*H*-pyrazole-3-carboxylate

Intermediate E was synthesized according to exemplary synthetic procedure (73.8 mg, 59% yield over two steps) LCMS ES-MS *m/z* 381.2 $[M+H]^+$. ^{1}H NMR (400 MHz, $CDCl_3$) δ 7.74 (d, *J* = 0.8 Hz, 1H), 7.60 (d, *J* = 0.8 Hz, 1H), 7.45 – 7.37 (m, 2H), 7.14 – 7.07 (m, 2H), 6.61 (s, 1H), 4.84 – 4.73 (m, 1H), 4.37 (q, *J* = 7.1 Hz, 2H), 4.16 (td, *J* = 8.1, 6.8 Hz, 1H), 4.03 (d, *J* = 2.2 Hz, 2H), 3.94 (s, 3H), 3.93 – 3.86 (m, 3H), 2.47 (ddt, *J* = 13.2, 7.9, 5.5 Hz, 1H), 2.25 – 2.11 (m, 1H), 1.38 (t, *J* = 7.1 Hz, 3H).

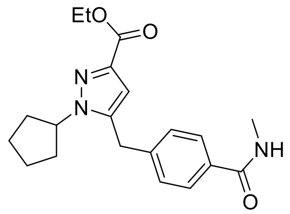

Intermediate F. Ethyl 1-cyclopentyl-5-(4-(methylcarbamoylbenzyl)-1*H*-pyrazole-3-carboxylate

Intermediate F was synthesized according to exemplary synthetic procedure (81.3 mg, 12% yield over two steps) LCMS ES-MS *m/z* 356.2 $[M+H]^+$. ^{1}H NMR (400 MHz, $CDCl_3$) δ 7.72 (d, *J* = 8.3 Hz, 2H), 7.18 (d, *J* = 8.2 Hz, 2H), 6.51 (s, 1H), 6.34 (d, *J* = 4.9 Hz, 1H), 4.42 (p, *J* = 7.7 Hz, 1H), 4.35 (q, *J* = 7.1 Hz, 2H), 4.05 (s, 2H), 3.00 (d, *J* = 4.8 Hz, 3H), 2.12 – 1.97 (m, 2H), 1.95 – 1.80 (m, 4H), 1.64 – 1.47 (m, 2H), 1.35 (t, *J* = 7.1 Hz, 3H).

Final Compounds

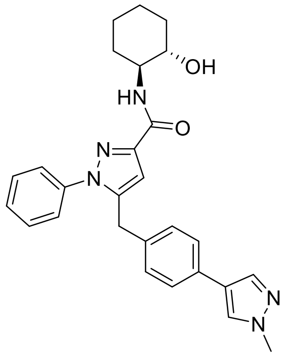

17a

Compound **17a** was synthesized according to the exemplary synthetic procedure. (15.4 mg, 53% yield). LCMS ES-MS m/z 456.2 $[M+H]^+$. ^{1}H NMR (400 MHz, $CDCl_3$) δ 7.73 (d, *J* = 0.8 Hz, 1H), 7.59 (d, *J* = 0.8 Hz, 1H), 7.51 – 7.43 (m, 3H), 7.39 – 7.33 (m, 4H), 7.08 – 7.02 (m, 2H), 6.92 (d, *J* = 7.7 Hz, 1H), 6.73 (s, 1H), 3.98 – 3.94 (m, 5H), 3.86 – 3.76 (m, 1H), 3.42 (m, 1H), 2.13 – 2.05 (m, 1H), 2.04 – 1.97 (m, 1H), 1.79 – 1.69 (m, 2H), 1.43 – 1.18 (m, 4H). *OH proton is not observable.

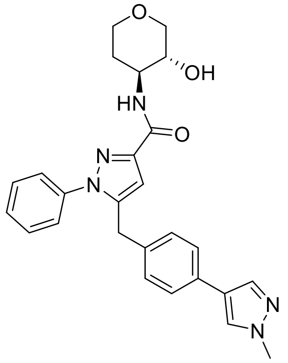

17b

Compound **17b** was synthesized according to exemplary synthetic procedure. (10.0 mg, 34% yield). LCMS ES-MS m/z 458.2 $[M+H]^+$. ^{1}H NMR (400 MHz, $CDCl_3$) δ 7.75 (s, 1H), 7.60 (s, 1H), 7.52 – 7.45 (m, 3H), 7.40 – 7.34 (m, 4H), 7.08 – 7.03 (m, 2H), 7.03 – 6.98 (m, 1H), 6.73 (s, 1H), 4.07 (dd, *J* = 11.3, 4.1 Hz, 1H), 4.01 – 3.93 (m, 7H), 3.58 (td, *J* = 9.6, 5.1 Hz, 1H), 3.43 (td, *J* = 11.9, 2.2 Hz, 1H), 3.19 (dd, *J* = 11.4, 10.0 Hz, 1H), 2.01 – 1.93 (m, 1H), 1.70 (qd, *J* = 12.3, 4.8 Hz, 1H). *OH proton is not observable.

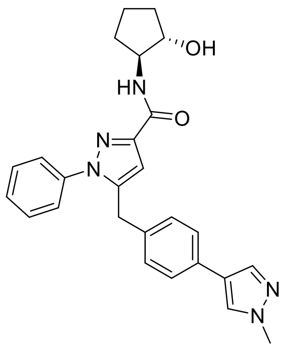

17c

Compound **17c** was synthesized according to exemplary synthetic procedure. (9.9 mg, 35% yield). LCMS ES-MS m/z 442.2 $[M+H]^+$. ^{1}H NMR (400 MHz, $CDCl_3$) δ 7.75 (d, *J* = 0.8 Hz, 1H), 7.60 (d, *J* = 0.8 Hz, 1H), 7.50 – 7.44 (m, 3H), 7.39 – 7.34 (m, 4H), 7.08 – 7.03 (m, 3H), 6.71 (d, *J* = 0.7 Hz, 1H), 4.12 – 4.05 (m, 1H), 3.99 – 3.94 (m, 6H), 2.24 – 2.13 (m, 1H), 2.12 – 2.02 (m, 1H), 1.87 – 1.78 (m, 1H), 1.78 – 1.66 (m, 2H), 1.61 – 1.50 (m, 1H). *OH proton is not observable.

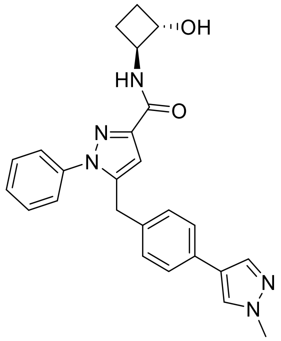

17d

Compound **17d** was synthesized according to the exemplary synthetic procedure. (12.4 mg, 45% yield). LCMS ES-MS m/z 428.2 $[M+H]^+$. ^{1}H NMR (400 MHz, $CDCl_3$) δ 7.74 (d, *J* = 0.8 Hz, 1H), 7.59 (d, *J* = 0.8 Hz, 1H), 7.50 – 7.44 (m, 3H), 7.38 – 7.33 (m, 4H), 7.17 (d, *J* = 4.1 Hz, 1H), 7.05 (d, *J* = 8.2 Hz, 2H), 6.70 (s, 1H), 4.12 – 4.04 (m, 1H), 4.00 – 3.93 (m, 6H), 2.23 – 2.05 (m, 2H), 1.81 – 1.67 (m, 1H), 1.53 – 1.38 (m, 1H). *OH proton is not observable.

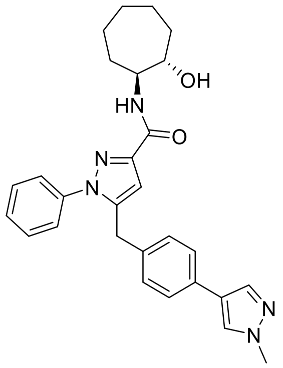

17e

Compound **17e** was synthesized according to exemplary synthetic procedure. (18.3 mg, 60% yield). LCMS ES-MS m/z 470.2 $[M+H]^+$. ^{1}H NMR (400 MHz, $CDCl_3$) δ 7.74 (s, 1H), 7.59 (s, 1H), 7.50 – 7.44 (m, 3H), 7.40 – 7.33 (m, 4H), 7.08 – 7.01 (m, 3H), 6.72 (s, 1H), 4.01 – 3.93 (m, 6H), 3.77 – 3.68 (m, 1H), 1.91 – 1.42 (m, 10H). *OH proton is not observable.

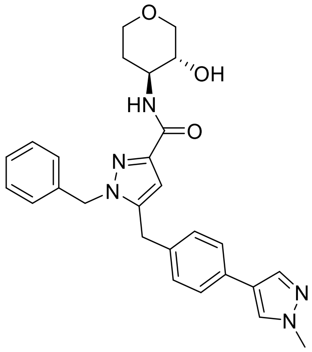

17f

Compound **17f** was synthesized according to the exemplary synthetic procedure. (11.3 mg, 59% yield). LCMS ES-MS m/z 472.2 $[M+H]^+$. ^{1}H NMR (400 MHz, $CDCl_3$) δ 7.75 (s, 1H), 7.60 (s, 1H), 7.39 – 7.29 (m, 5H), 7.07 – 6.99 (m, 4H), 6.95 (d, *J* = 6.5 Hz, 1H), 6.63 (s, 1H), 5.23 (s, 2H), 4.11 – 4.05 (m, 1H), 4.02 – 3.96 (m, 4H), 3.95 – 3.89 (m, 1H), 3.85 (s, 2H), 3.60 (td, *J* = 9.6, 5.0 Hz, 1H), 3.44 (td, *J* = 11.9, 2.2 Hz, 1H), 3.20 (dd, *J* = 11.4, 10.0 Hz, 1H), 2.05 – 1.97 (m, 1H), 1.74 (qd, *J* = 12.2, 4.8 Hz, 1H). *OH proton is not observable.

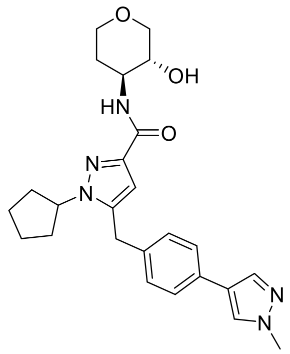

17g

Compound **17g** was synthesized according to exemplary synthetic procedure. (1.9 mg, 12% yield). LCMS ES-MS m/z 450.4 $[M+H]^+$. ^{1}H NMR (400 MHz, $CDCl_3$) δ 7.74 (d, *J* = 0.8 Hz, 1H), 7.59 (d, *J* = 0.8 Hz, 1H), 7.40 (d, *J* = 8.2 Hz, 2H), 7.11 (d, *J* = 8.2 Hz, 2H), 6.91 (d, *J* = 6.3 Hz, 1H), 6.56 (s, 1H), 4.50 (p, *J* = 7.2 Hz, 1H), 4.07 (ddd, *J* = 11.4, 5.1, 1.1 Hz, 1H), 4.03 – 3.85 (m, 7H), 3.59 (td, *J* = 9.6, 5.0 Hz, 1H), 3.44 (td, *J* = 11.9, 2.2 Hz, 1H), 3.19 (dd, *J* = 11.4, 10.0 Hz, 1H), 2.05 – 1.82 (m, 7H), 1.82 – 1.69 (m, 1H) , 1.68 – 1.53 (m, 2H). *OH proton is not observable. ^{13}C NMR (101 MHz, $CDCl_3$) δ 164.8, 144.1, 143.1, 136.8, 135.2, 131.5, 129.0, 127.0, 126.0, 122.9, 107.2, 72.5, 71.5, 66.5, 59.9, 54.0, 39.2, 33.0, 32.9, 31.7, 31.2, 24.7.

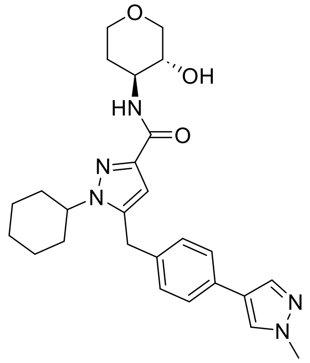

17h

Compound **17h** was synthesized according to exemplary synthetic procedure. (24.8 mg, 42% yield). LCMS ES-MS m/z 464.3 $[M+H]^+$. ^{1}H NMR (400 MHz, $CDCl_3$) δ 7.74 (d, *J* = 0.8 Hz, 1H), 7.59 (d, *J* = 0.8 Hz, 1H), 7.44 – 7.36 (m, 2H), 7.17 – 7.09 (m, 2H), 6.93 (d, *J* = 6.2 Hz, 1H), 6.55 (s, 1H), 4.62 (s, 1H), 4.08 (ddd, *J* = 11.3, 5.1, 1.1 Hz, 1H), 4.02 – 3.96 (m, 3H), 3.96 – 3.87 (m, 5H), 3.60 (dt, *J* = 9.5, 4.8 Hz, 1H), 3.44 (td, *J* = 11.9, 2.2 Hz, 1H), 3.19 (dd, *J* = 11.4, 10.0 Hz, 1H), 2.06 – 1.96 (m, 1H), 1.92 – 1.80 (m, 4H), 1.79 – 1.68 (m, 4H), 1.35 – 1.19 (m, 3H).

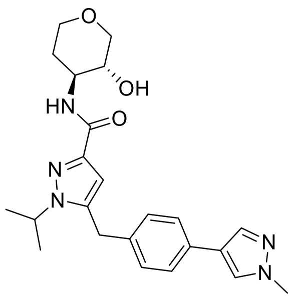

17i

Compound **17i** was synthesized according to exemplary synthetic procedure. (6.3 mg, 23% yield). LCMS ES-MS m/z 424.4 $[M+H]^+$. ^1H NMR (400 MHz, MeOD) δ 7.69 (d, *J* = 8.2 Hz, 2H), 7.58 (d, *J* = 2.3 Hz, 1H), 7.28 (d, *J* = 8.3 Hz, 2H), 6.58 (d, *J* = 2.3 Hz, 1H), 6.47 (s, 1H), 5.41 (hept, *J* = 6.7 Hz, 1H), 3.98 (s, 2H), 3.95 – 3.83 (m, 6H), 3.51 (td, *J* = 9.8, 5.0 Hz, 1H), 3.42 (td, *J* = 12.0, 2.2 Hz, 1H), 3.13 (dd, *J* = 11.1, 10.1 Hz, 1H), 1.96 – 1.86 (m, 1H), 1.68 – 1.52 (m, 1H), 1.47 (dd, *J* = 6.7, 4.0 Hz, 6H). *OH and NH protons are not observable.

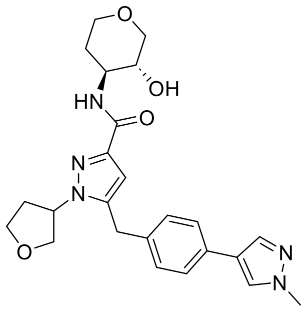

17j

Compound **17j** was synthesized according to the exemplary synthetic procedure. (42.2 mg, 39% yield). LCMS ES-MS m/z 452.3 $[M+H]^+$. ^1H NMR (400 MHz, $CDCl_3$) δ 7.73 (s, 1H), 7.59 (d, *J* = 0.9 Hz, 1H), 7.44 – 7.37 (m, 2H), 7.13 – 7.07 (m, 2H), 7.00 (dd, *J* = 28.5, 6.5 Hz, 1H), 6.63 (d, *J* = 7.4 Hz, 1H), 4.83 – 4.72 (m, 1H), 4.17 – 4.05 (m, 2H), 4.02 (s, 2H), 4.01 – 3.84 (m, 8H), 3.59 (tdd, *J* = 9.7, 5.0, 1.8 Hz, 1H), 3.50 – 3.39 (m, 1H), 3.25 – 3.14 (m, 1H), 2.33 – 2.23 (m, 1H), 2.23 – 2.13 (m, 1H), 2.06 – 1.95 (m, 1H), 1.75 (qd, *J* = 12.3, 4.8 Hz, 1H). *OH proton is not observable.

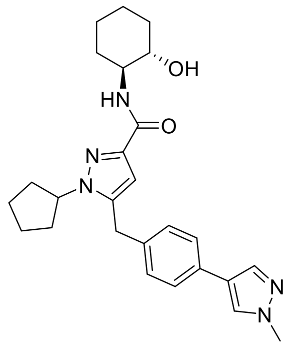

17k

Compound **17k** was synthesized according to exemplary synthetic procedure. (3.3 mg, 20% yield). LCMS ES-MS m/z 448.4 $[M+H]^+$. ^{1}H NMR (400 MHz, $CDCl_3$) δ 7.74 (d, *J* = 0.8 Hz, 1H), 7.59 (s, 1H), 7.39 (d, *J* = 8.1 Hz, 2H), 7.11 (d, *J* = 8.2 Hz, 2H), 6.84 (d, *J* = 7.3 Hz, 1H), 6.56 (s, 1H), 4.49 (p, *J* = 7.2 Hz, 1H), 4.00 (s, 2H), 3.94 (s, 3H), 3.86 – 3.71 (m, 1H), 3.50 – 3.41 (m, 1H), 2.15 – 1.82 (m, 8H), 1.81 – 1.70 (m, 2H), 1.66 – 1.53 (m, 2H), 1.46 – 1.19 (m, 4H). *OH proton is not observable.

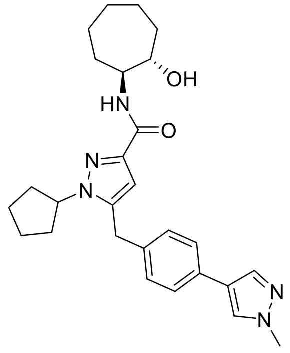

17l

Compound **17l** was synthesized according to the exemplary synthetic procedure. (3.1 mg, 18% yield). LCMS ES-MS m/z 462.4 $[M+H]^+$. ^{1}H NMR (400 MHz, $CDCl_3$) δ 7.74 (d, *J* = 0.8 Hz, 1H), 7.59 (s, 1H), 7.39 (d, *J* = 8.2 Hz, 2H), 7.11 (d, *J* = 8.2 Hz, 2H), 6.96 (d, *J* = 6.9 Hz, 1H), 6.55 (s, 1H), 4.49 (p, *J* = 7.1 Hz, 1H), 4.00 (s, 2H), 3.97 – 3.89 (m, 4H), 3.78 – 3.71 (m, 1H), 2.04 – 1.47 (m, 18H).

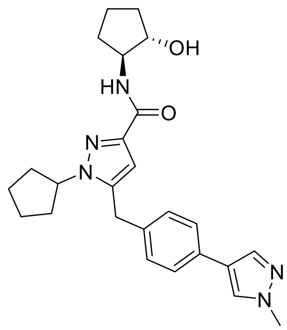

17m

Compound **17m** was synthesized according to exemplary synthetic procedure. (2.1 mg, 13% yield). LCMS ES-MS m/z 434.4 $[M+H]^+$. ^{1}H NMR (400 MHz, $CDCl_3$) δ 7.74 (d, *J* = 0.8 Hz, 1H), 7.59 (d, *J* = 0.8 Hz, 1H), 7.39 (d, *J* = 8.2 Hz, 2H), 7.11 (d, *J* = 8.2 Hz, 2H), 6.97 (d, *J* = 4.4 Hz, 1H), 6.54 (s, 1H), 4.49 (p, *J* = 7.2 Hz, 1H), 4.09 (q, *J* = 6.8 Hz, 1H), 4.00 (s, 2H), 3.98 – 3.91 (m, 4H), 2.29 – 2.16 (m, 1H), 2.14 – 2.04 (m, 1H), 2.03 – 1.44 (m, 12H). *OH proton is not observable.

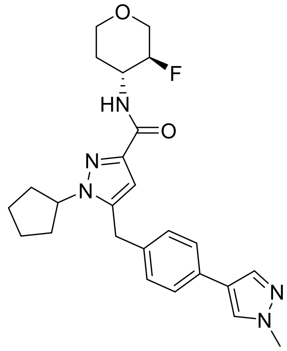

17n

Compound **17n** was synthesized according to the exemplary synthetic procedure. (3.3 mg, 20% yield). LCMS ES-MS m/z 452.4 $[M+H]^+$. ^{1}H NMR (400 MHz, $CDCl_3$) δ 7.74 (d, *J* = 0.9 Hz, 1H), 7.59 (d, *J* = 0.8 Hz, 1H), 7.39 (d, *J* = 8.2 Hz, 2H), 7.11 (d, *J* = 8.3 Hz, 2H), 6.91 (d, *J* = 8.0 Hz, 1H), 6.57 (s, 1H), 4.61 – 4.39 (m, 2H), 4.39 – 4.25 (m, 1H), 4.10 (dd, *J* = 7.8, 3.6 Hz, 1H), 4.00 (s, 2H), 3.94 (s, 3H), 3.92 – 3.85 (m, 1H), 3.60 – 3.52 (m, 1H), 3.51 – 3.44 (m, 1H), 2.31 – 2.19 (m, 1H), 2.05 – 1.94 (m, 2H), 1.94 – 1.82 (m, 4H), 1.76 – 1.54 (m, 3H).

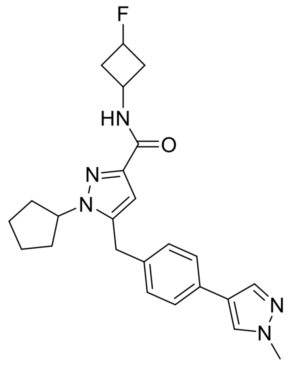

17o

Compound **17o** was synthesized according to the exemplary synthetic procedure. (2.9 mg, 15% yield). LCMS ES-MS m/z 422.4 $[M+H]^+$. ^{1}H NMR (400 MHz, $CDCl_3$) (a mixture of cis/trans isomers) δ 7.74 (d, *J* = 0.8 Hz, 1H), 7.59 (d, *J* = 0.8 Hz, 1H), 7.39 (d, *J* = 8.1 Hz, 2H), 7.10 (d, *J* = 8.4 Hz, 2H), 6.95 (t, *J* = 6.9 Hz, 1H), 6.58 – 6.52 (m, 1H), 5.37 – 5.31 (m, 0.2H), 5.23 – 5.16 (m, 0.2H), 4.95 – 4.85 (m, 0.3H), 4.81 – 4.73 (m, 0.3H), 4.72 – 4.64 (m, 0.5H), 4.56 – 4.42 (m, 1H), 4.22 – 4.07 (m, 0.5H), 4.00 (s, 2H), 3.94 (s, 3H), 2.98 – 2.86 (m, 1H), 2.79 – 2.61 (m, 1H), 2.48 (d, *J* = 6.3 Hz, 1H), 2.34 – 2.15 (m, 1H), 2.05 – 1.82 (m, 6H), 1.72 – 1.48 (m, 2H).

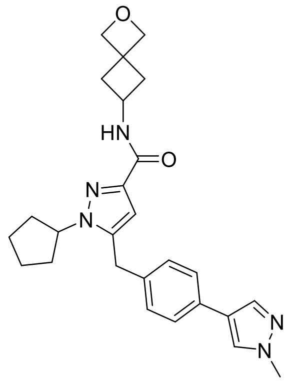

17p

Compound **17p** was synthesized according to the exemplary synthetic procedure. (2.9 mg, 18% yield). LCMS ES-MS m/z 446.4 $[M+H]^+$. ^{1}H NMR (400 MHz, $CDCl_3$) δ 7.73 (d, *J* = 0.8 Hz, 1H), 7.59 (s, 1H), 7.39 (d, *J* = 8.2 Hz, 2H), 7.10 (d, *J* = 8.2 Hz, 2H), 6.89 (d, *J* = 7.9 Hz, 1H), 6.53 (s, 1H), 4.77 (s, 2H), 4.64 (s, 2H), 4.54 – 4.42 (m, 1H), 4.41 – 4.30 (m, 1H), 3.99 (s, 2H), 3.94 (s, 3H), 2.81 – 2.70 (m, 2H), 2.26 – 2.15 (m, 2H), 2.01 – 1.83 (m, 6H), 1.68 – 1.51 (m, 2H).

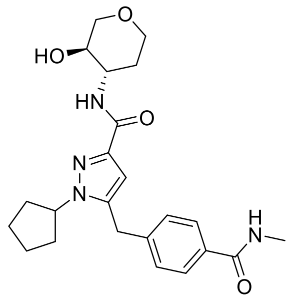

21

Compound **21** was synthesized according to the exemplary synthetic procedure. (5.5 mg, 6 % yield) LCMS ES-MS m/z 427.3 $[M+H]^+$. ^{1}H NMR (400 MHz, $CDCl_3$) δ 7.68 (d, *J* = 8.1 Hz, 2H), 7.13 (d, *J* = 8.0 Hz, 2H), 6.96 (d, *J* = 6.4 Hz, 1H), 6.65 (q, *J* = 4.8 Hz, 1H), 6.47 (s, 1H), 4.43 (p, *J* = 7.2 Hz, 1H), 4.07 – 4.00 (m, 3H), 3.99 – 3.82 (m, 2H), 3.56 (td, *J* = 9.6, 5.0 Hz, 1H), 3.41 (td, *J* = 11.9, 2.2 Hz, 1H), 3.16 (dd, *J* = 11.3, 9.9 Hz, 1H), 2.95 (d, *J* = 4.8 Hz, 3H), 2.06 – 1.78 (m, 7H), 1.71 (qd, *J* = 12.1, 4.8 Hz, 1H), 1.63 – 1.52 (m, 2H). *OH proton is not observable.

Final Compound Characterizations – ^{1}H-NMR, ^{13}C-NMR, and/or LCMS

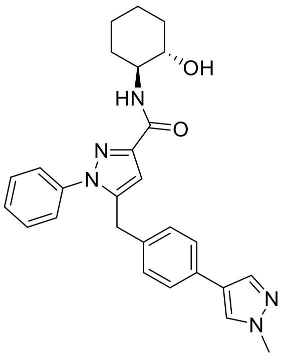

17a

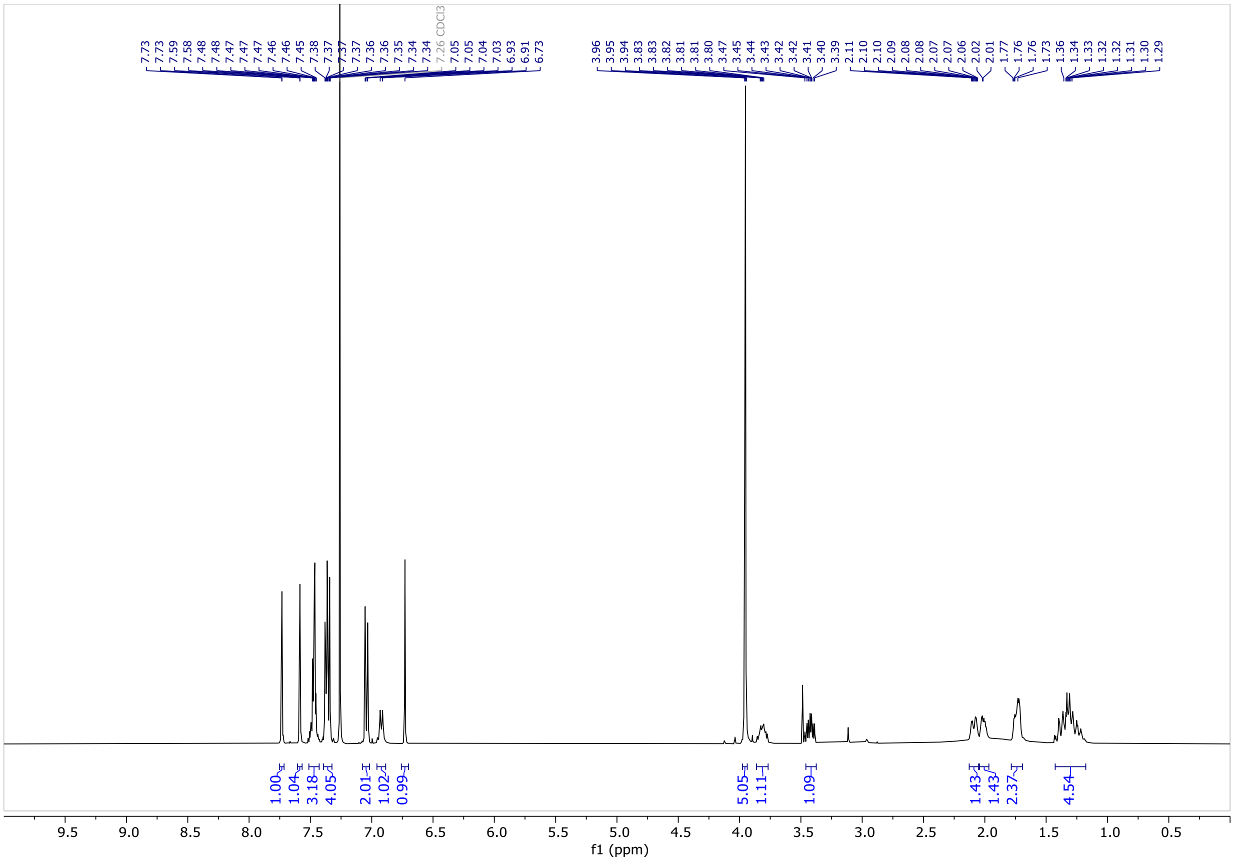

^{1}H NMR (400 MHz, $CDCl_3$) δ 7.73 (d, *J* = 0.8 Hz, 1H), 7.59 (d, *J* = 0.8 Hz, 1H), 7.51 – 7.43 (m, 3H), 7.39 – 7.33 (m, 4H), 7.08 – 7.02 (m, 2H), 6.92 (d, *J* = 7.7 Hz, 1H), 6.73 (s, 1H), 3.98 – 3.94 (m, 5H), 3.86 – 3.76 (m, 1H), 3.42 (m, 1H), 2.13 – 2.05 (m, 1H), 2.04 – 1.97 (m, 1H), 1.79 – 1.69 (m, 2H), 1.43 – 1.18 (m, 4H). *OH proton is not observable.

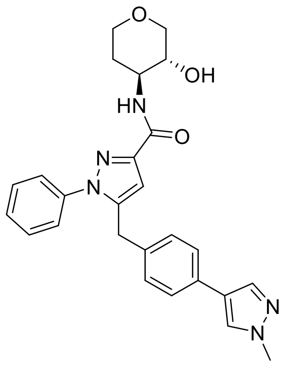

17b

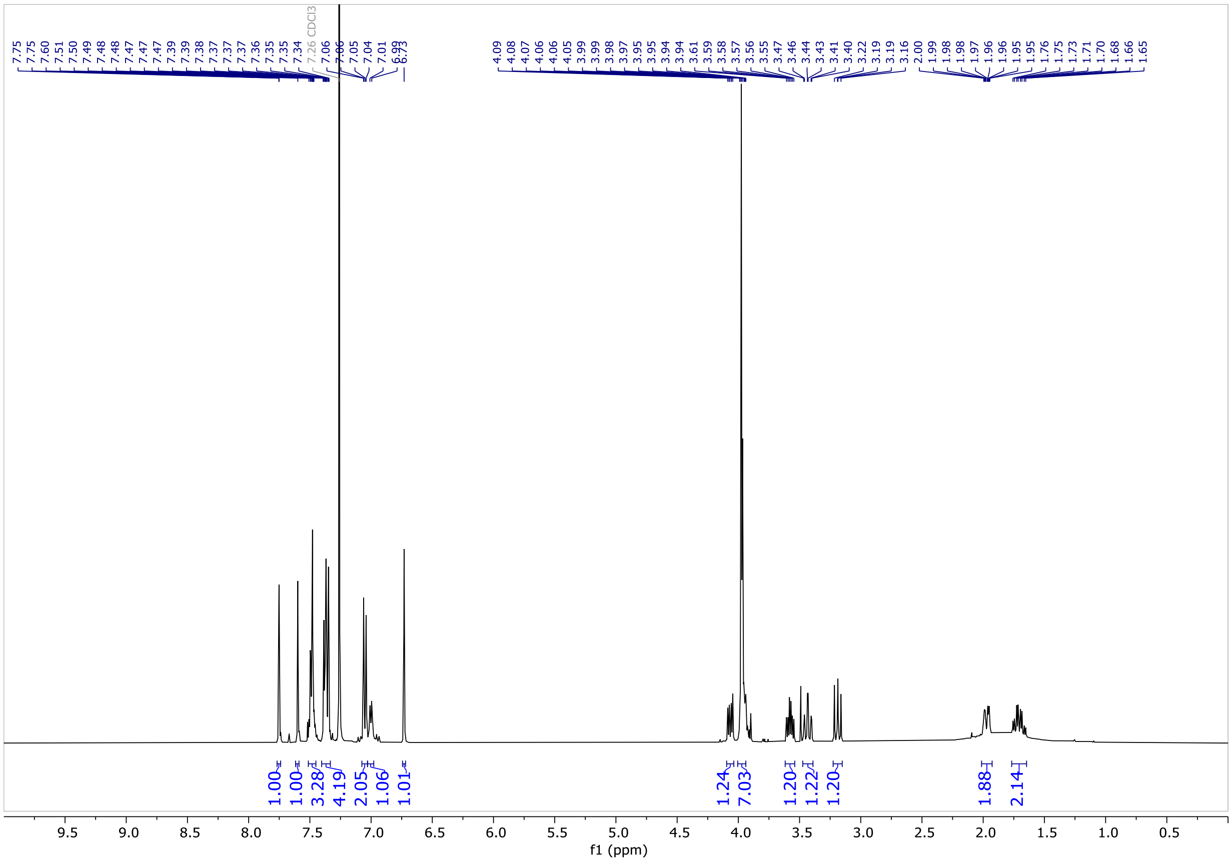

^{1}H NMR (400 MHz, $CDCl_3$) δ 7.75 (s, 1H), 7.60 (s, 1H), 7.52 – 7.45 (m, 3H), 7.40 – 7.34 (m, 4H), 7.08 – 7.03 (m, 2H), 7.03 – 6.98 (m, 1H), 6.73 (s, 1H), 4.07 (dd, *J* = 11.3, 4.1 Hz, 1H), 4.01 – 3.93 (m, 7H), 3.58 (td, *J* = 9.6, 5.1 Hz, 1H), 3.43 (td, *J* = 11.9, 2.2 Hz, 1H), 3.19 (dd, *J* = 11.4, 10.0 Hz, 1H), 2.01 – 1.93 (m, 1H)[a], 1.70 (qd, *J* = 12.3, 4.8 Hz, 1H).[a] *OH proton is not observable.

[a] Integral value shown in the spectrum is higher than expected. This is due to the peak enhancement caused by overlapping with the residual water peak.

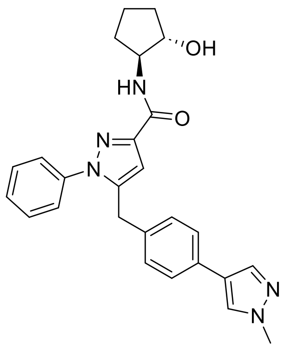

17c

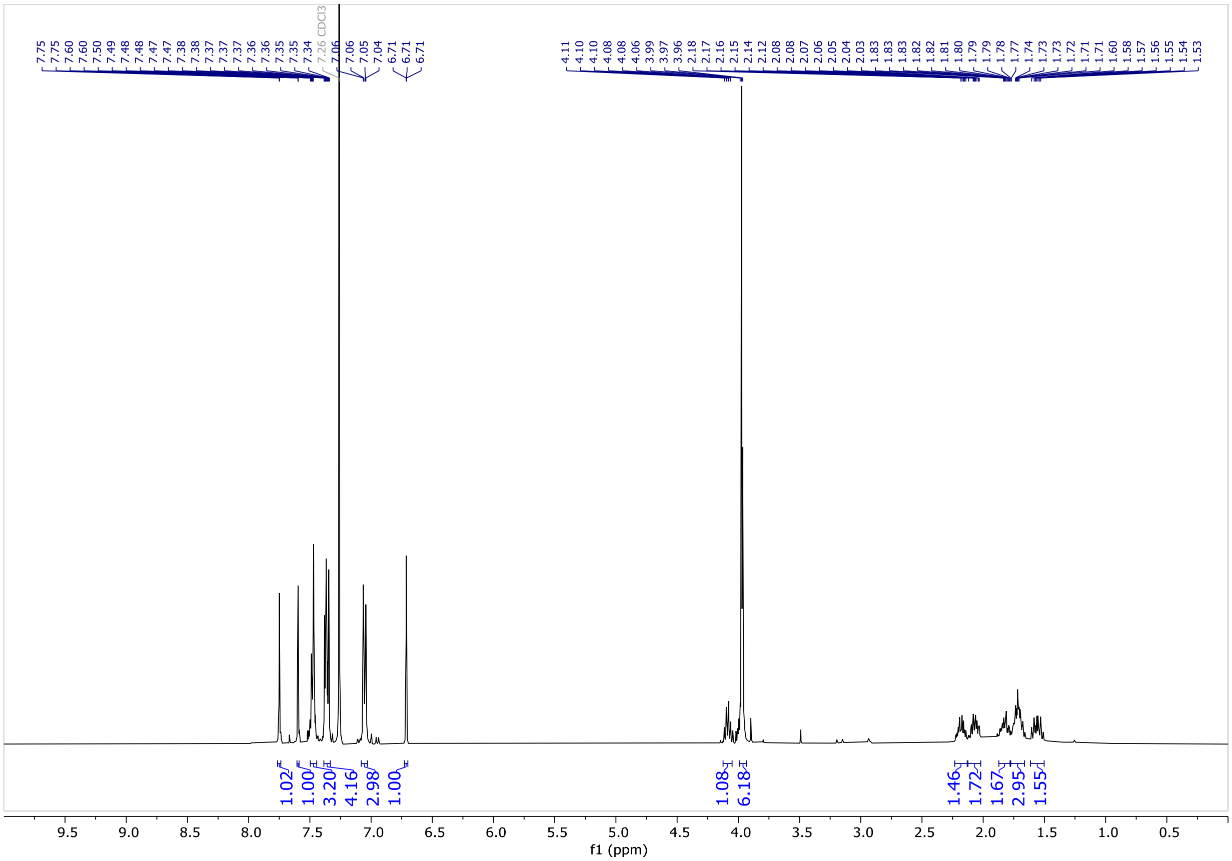

^{1}H NMR (400 MHz, $CDCl_3$) δ 7.75 (d, *J* = 0.8 Hz, 1H), 7.60 (d, *J* = 0.8 Hz, 1H), 7.50 – 7.44 (m, 3H), 7.39 – 7.34 (m, 4H), 7.08 – 7.03 (m, 3H), 6.71 (d, *J* = 0.7 Hz, 1H), 4.12 – 4.05 (m, 1H), 3.99 – 3.94 (m, 6H), 2.24 – 2.13 (m, 1H)[a], 2.12 – 2.02 (m, 1H)[a], 1.87 – 1.78 (m, 1H)[a] 1.78 – 1.66 (m, 2H)[a], 1.61 – 1.50 (m, 1H).[a] *OH proton is not observable.

[a] Integral value shown in the spectrum is higher than expected. This is due to the peak enhancement caused by overlapping with the residual water peak.

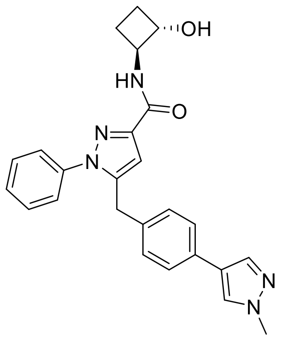

17d

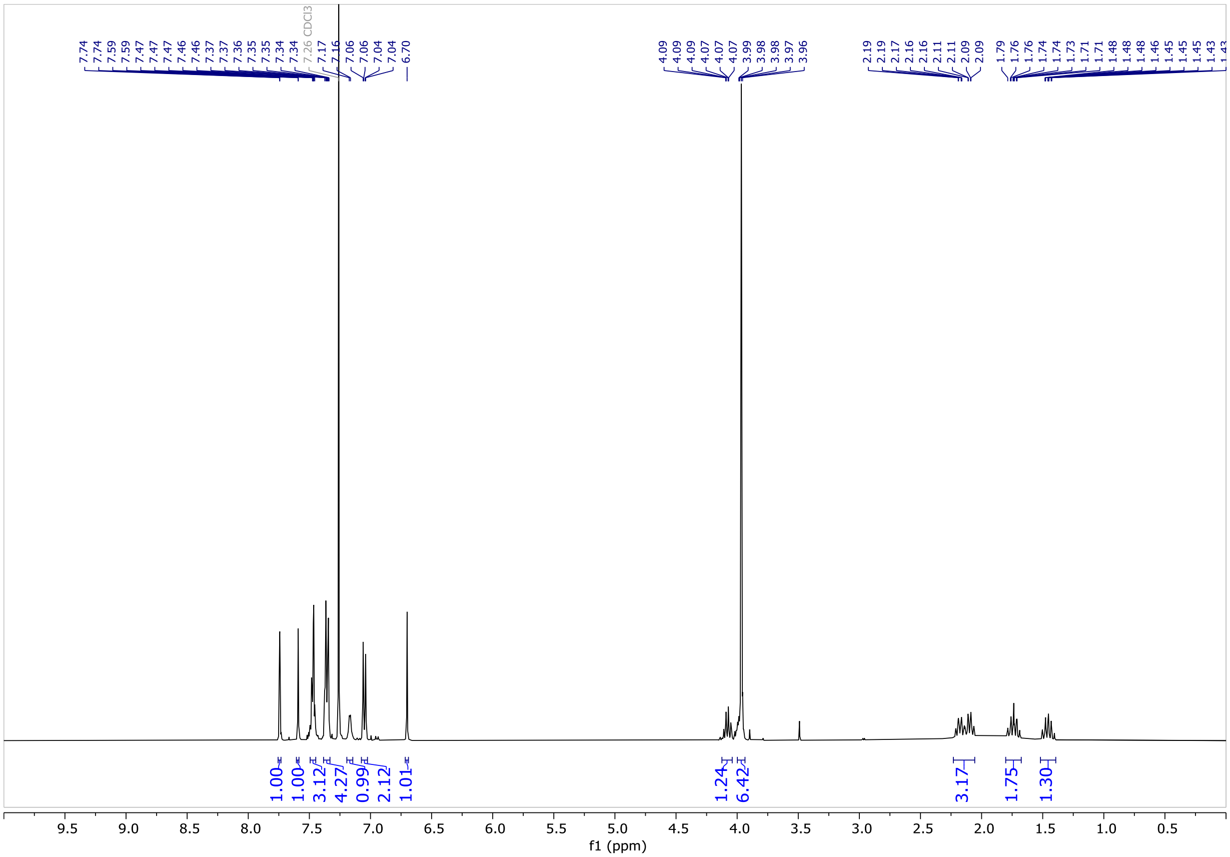

^{1}H NMR (400 MHz, $CDCl_3$) δ 7.74 (d, J = 0.8 Hz, 1H), 7.59 (d, J = 0.8 Hz, 1H), 7.50 – 7.44 (m, 3H), 7.38 – 7.33 (m, 4H), 7.17 (d, J = 4.1 Hz, 1H), 7.05 (d, J = 8.2 Hz, 2H), 6.70 (s, 1H), 4.12 – 4.04 (m, 1H), 4.00 – 3.93 (m, 6H), 2.23 – 2.05 (m, 2H)[a], 1.81 – 1.67 (m, 1H)[a], 1.53 – 1.38 (m, 1H).[a] *OH proton is not observable.

[a] Integral value shown in the spectrum is higher than expected. This is due to the peak enhancement caused by overlapping with the residual water peak.

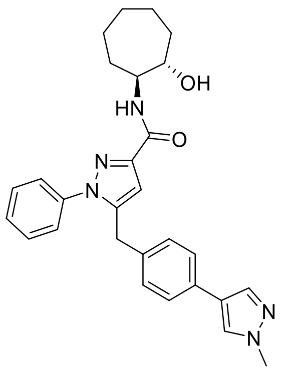

17e

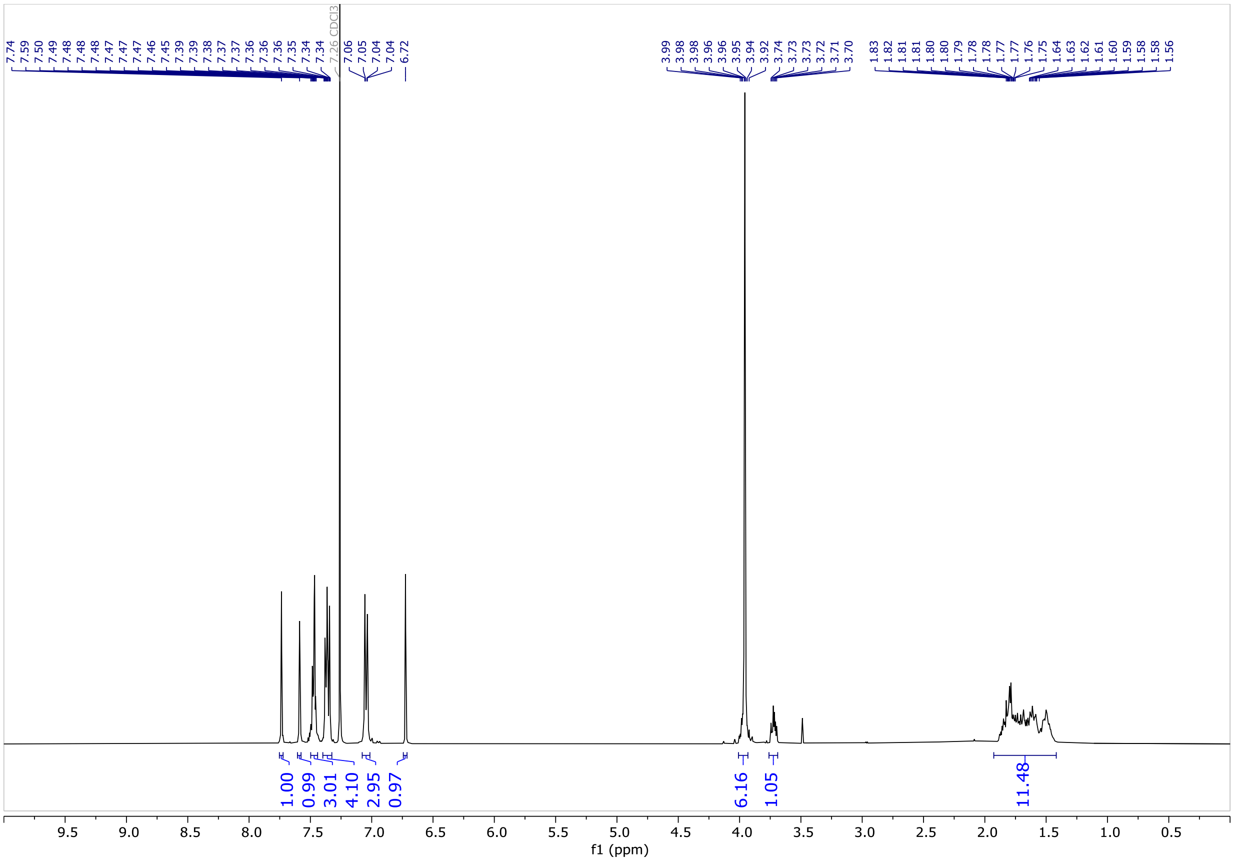

^{1}H NMR (400 MHz, $CDCl_3$) δ 7.74 (s, 1H), 7.59 (s, 1H), 7.50 – 7.44 (m, 3H), 7.40 – 7.33 (m, 4H), 7.08 – 7.01 (m, 3H), 6.72 (s, 1H), 4.01 – 3.93 (m, 6H), 3.77 – 3.68 (m, 1H), 1.91 – 1.42 (m, 10H).[a] *OH proton is not observable.

[a] Integral value shown in the spectrum is higher than expected. This is due to the peak enhancement caused by overlapping with the residual water peak.

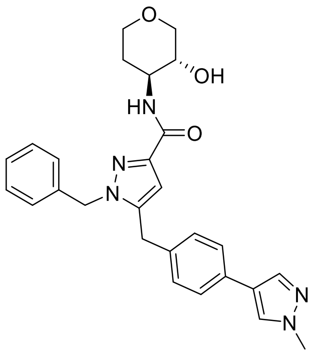

17f

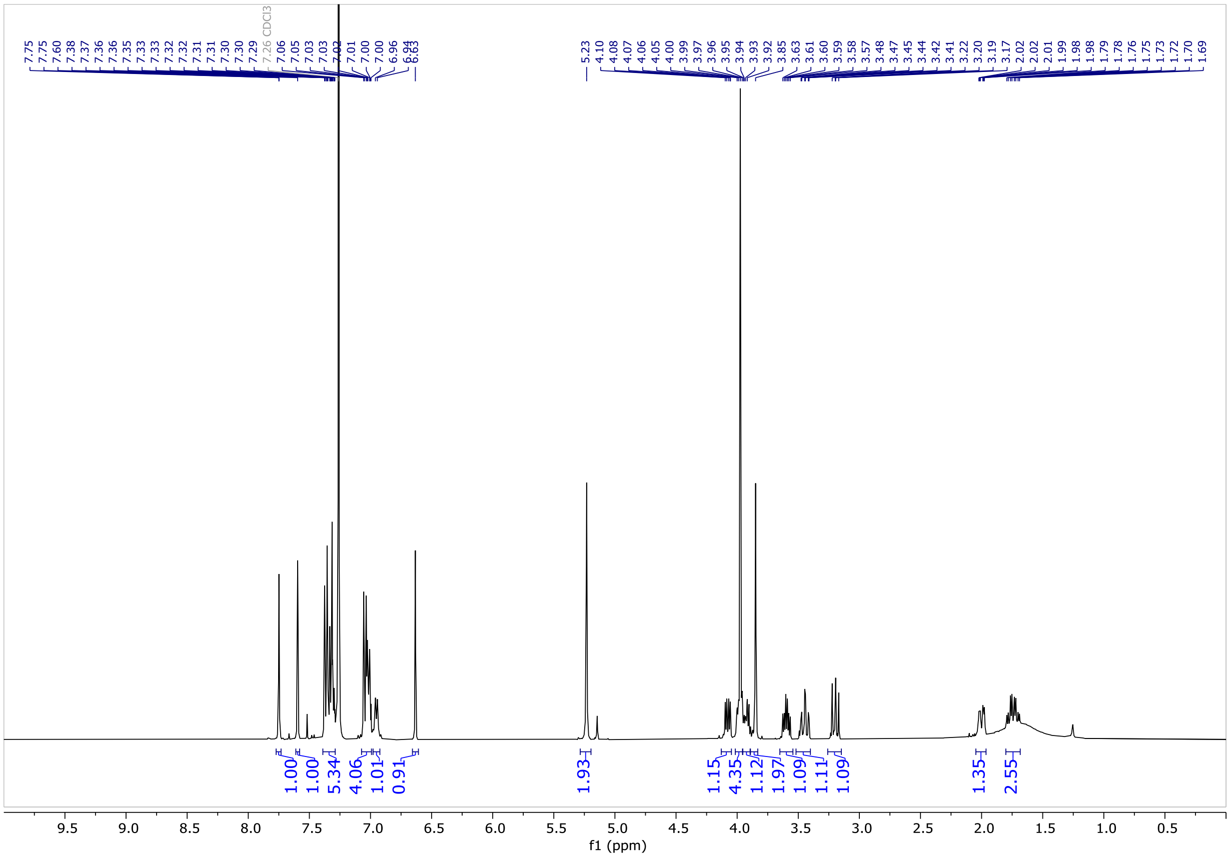

^{1}H NMR (400 MHz, $CDCl_3$) δ 7.75 (s, 1H), 7.60 (s, 1H), 7.39 – 7.29 (m, 5H), 7.07 – 6.99 (m, 4H), 6.95 (d, *J* = 6.5 Hz, 1H), 6.63 (s, 1H), 5.23 (s, 2H), 4.11 – 4.05 (m, 1H), 4.02 – 3.96 (m, 4H), 3.95 – 3.89 (m, 1H), 3.85 (s, 2H), 3.60 (td, *J* = 9.6, 5.0 Hz, 1H), 3.44 (td, *J* = 11.9, 2.2 Hz, 1H), 3.20 (dd, *J* = 11.4, 10.0 Hz, 1H), 2.05 – 1.97 (m, 1H)[a], 1.74 (qd, *J* = 12.2, 4.8 Hz, 1H).[a]
*OH proton is not observable.

[a] Integral value shown in the spectrum is higher than expected. This is due to the peak enhancement caused by overlapping with the residual water peak.

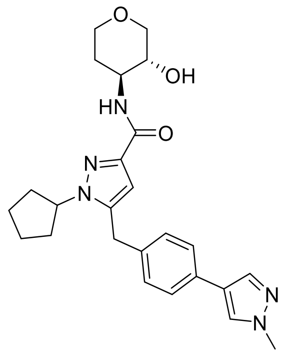

17g

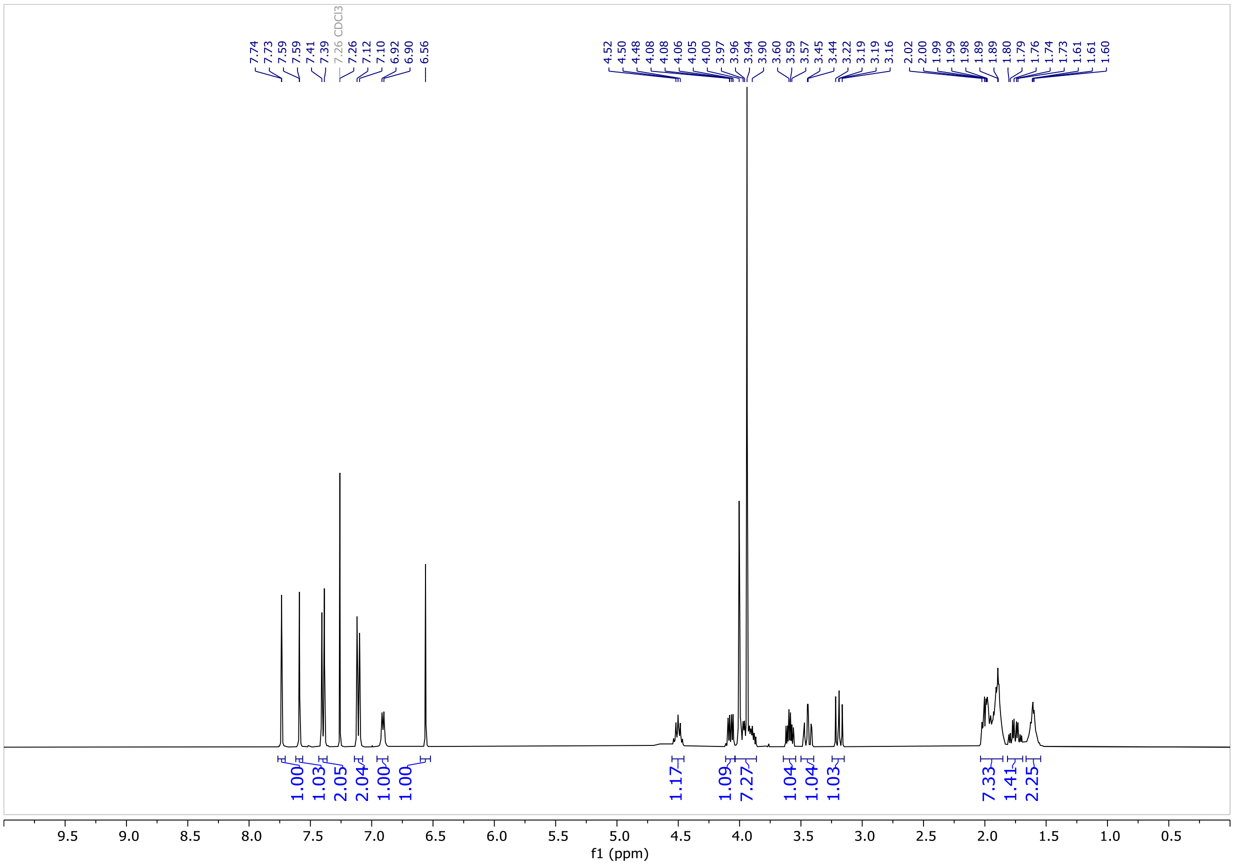

^{1}H NMR (400 MHz, $CDCl_3$) δ 7.74 (d, *J* = 0.8 Hz, 1H), 7.59 (d, *J* = 0.8 Hz, 1H), 7.40 (d, *J* = 8.2 Hz, 2H), 7.11 (d, *J* = 8.2 Hz, 2H), 6.91 (d, *J* = 6.3 Hz, 1H), 6.56 (s, 1H), 4.50 (p, *J* = 7.2 Hz, 1H), 4.07 (ddd, *J* = 11.4, 5.1, 1.1 Hz, 1H), 4.03 – 3.85 (m, 7H), 3.59 (td, *J* = 9.6, 5.0 Hz, 1H), 3.44 (td, *J* = 11.9, 2.2 Hz, 1H), 3.19 (dd, *J* = 11.4, 10.0 Hz, 1H), 2.05 – 1.82 (m, 7H)[a], 1.82 – 1.69 (m, 1H)[a], 1.68 – 1.53 (m, 2H). *OH proton is not observable.

---

[a] Integral value shown in the spectrum is higher than expected. This is due to the peak enhancement caused by overlapping with the residual water peak.

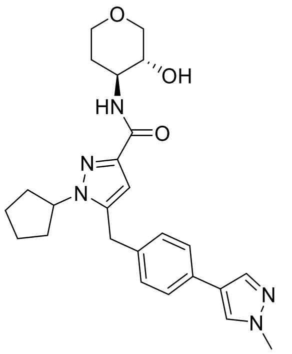

17g

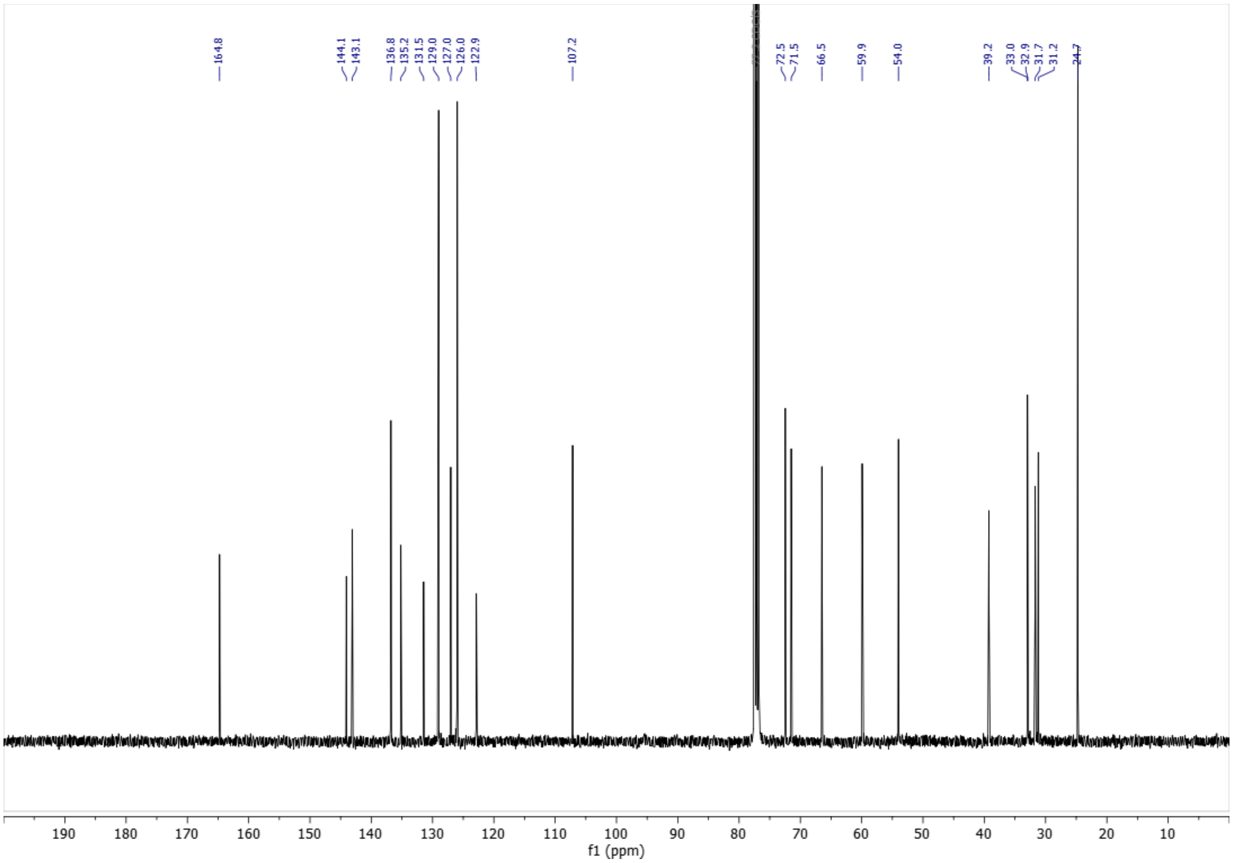

^{13}C NMR (101 MHz, $CDCl_3$) δ 164.8, 144.1, 143.1, 136.8, 135.2, 131.5, 129.0, 127.0, 126.0, 122.9, 107.2, 72.5, 71.5, 66.5, 59.9, 54.0, 39.2, 33.0, 32.9, 31.7, 31.2, 24.7.

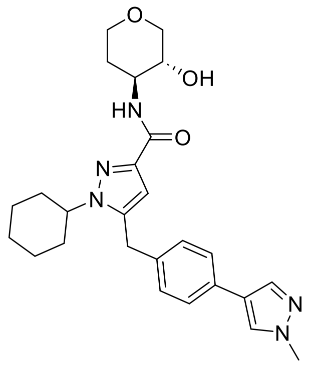

17h

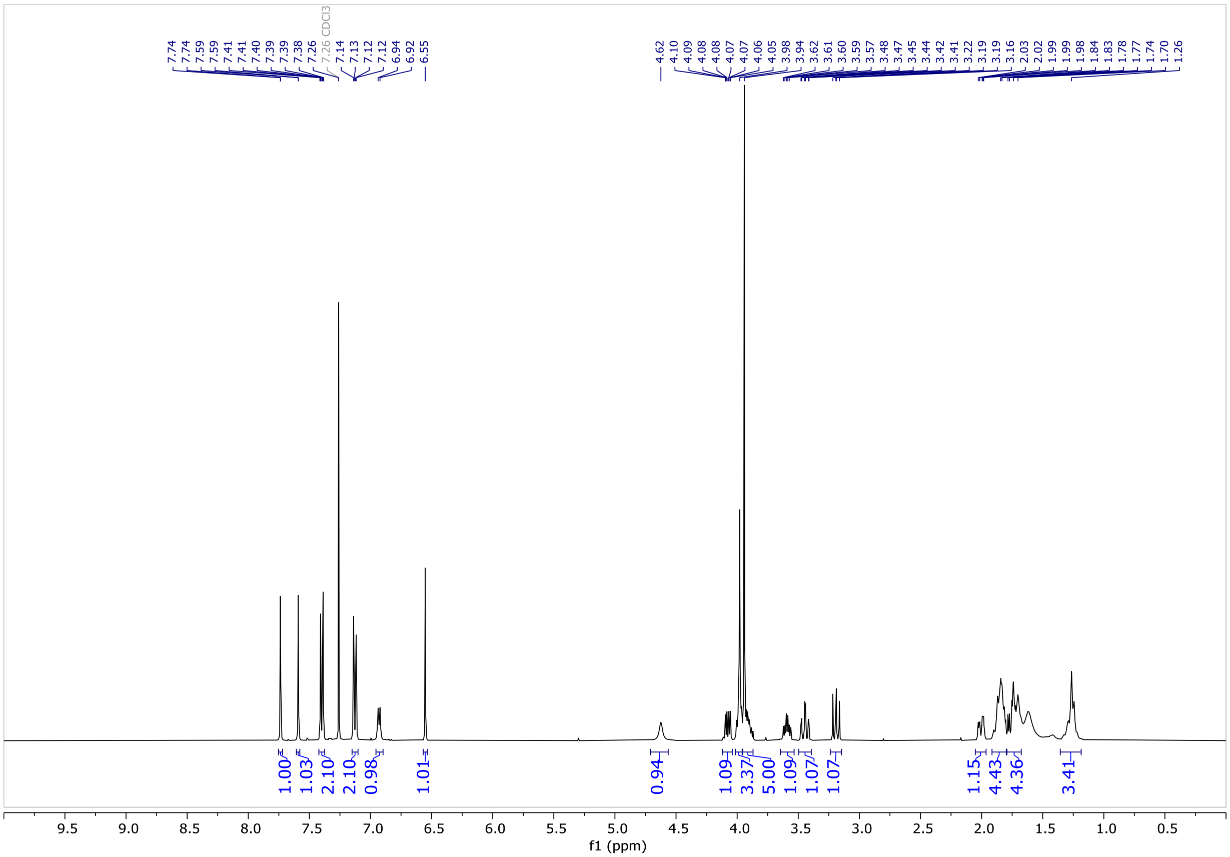

^{1}H NMR (400 MHz, $CDCl_3$) δ 7.74 (d, *J* = 0.8 Hz, 1H), 7.59 (d, *J* = 0.8 Hz, 1H), 7.44 – 7.36 (m, 2H), 7.17 – 7.09 (m, 2H), 6.93 (d, *J* = 6.2 Hz, 1H), 6.55 (s, 1H), 4.62 (s, 1H), 4.08 (ddd, J = 11.3, 5.1, 1.1 Hz, 1H), 4.02 – 3.96 (m, 3H), 3.96 – 3.87 (m, 5H), 3.60 (dt, *J* = 9.5, 4.8 Hz, 1H), 3.44 (td, *J* = 11.9, 2.2 Hz, 1H), 3.19 (dd, *J* = 11.4, 10.0 Hz, 1H), 2.06 – 1.96 (m, 1H), 1.92 – 1.80 (m, 4H), 1.79 – 1.68 (m, 4H)[a], 1.35 – 1.19 (m, 3H).

[a] Integral value shown in the spectrum is higher than expected. This is due to the peak enhancement caused by overlapping with the residual water peak.

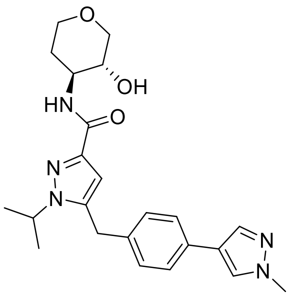

17i

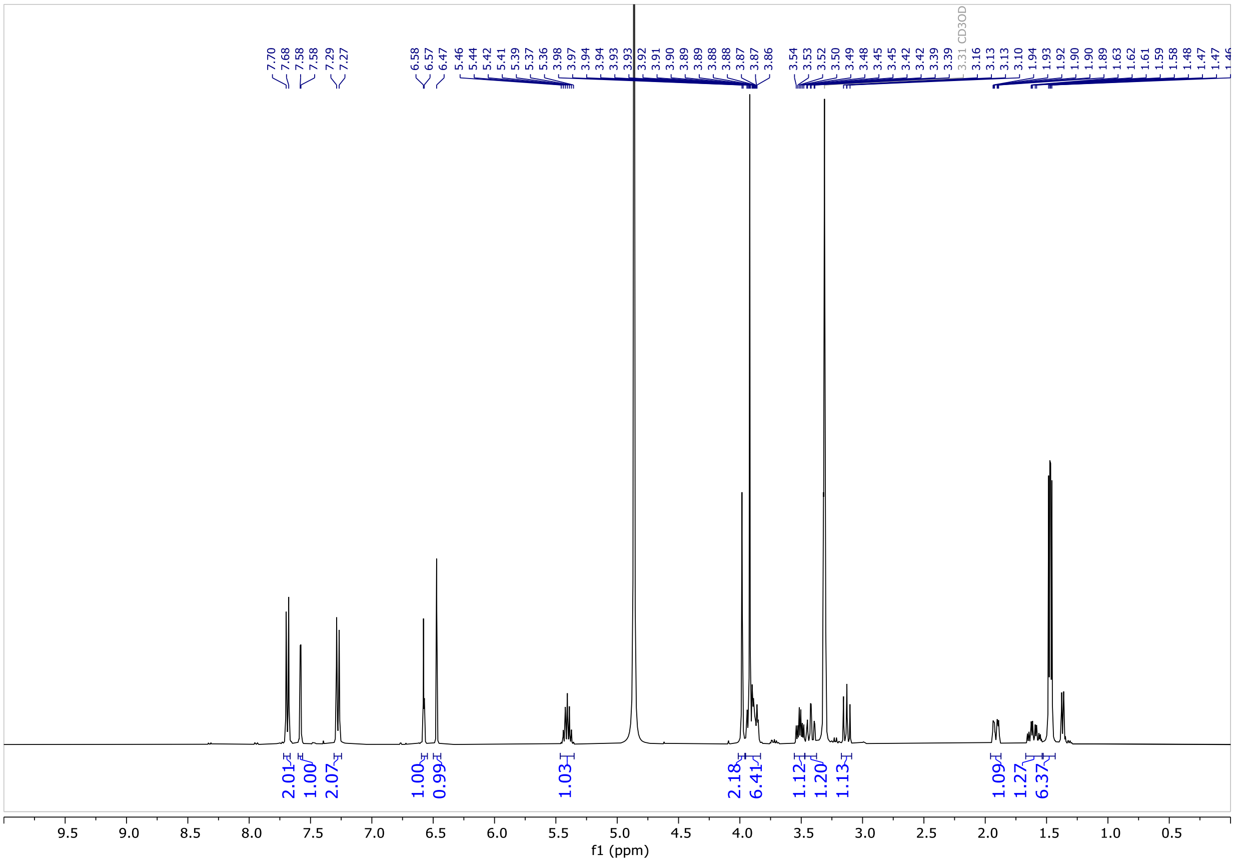

^{1}H NMR (400 MHz, MeOD) δ 7.69 (d, *J* = 8.2 Hz, 2H), 7.58 (d, *J* = 2.3 Hz, 1H), 7.28 (d, *J* = 8.3 Hz, 2H), 6.58 (d, *J* = 2.3 Hz, 1H), 6.47 (s, 1H), 5.41 (hept, *J* = 6.7 Hz, 1H), 3.98 (s, 2H), 3.95 – 3.83 (m, 6H), 3.51 (td, *J* = 9.8, 5.0 Hz, 1H), 3.42 (td, *J* = 12.0, 2.2 Hz, 1H), 3.13 (dd, *J* = 11.1, 10.1 Hz, 1H), 1.96 – 1.86 (m, 1H), 1.68 – 1.52 (m, 1H), 1.47 (dd, *J* = 6.7, 4.0 Hz, 6H). *OH and NH protons are not observable.

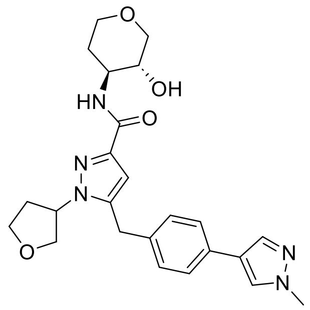

17j

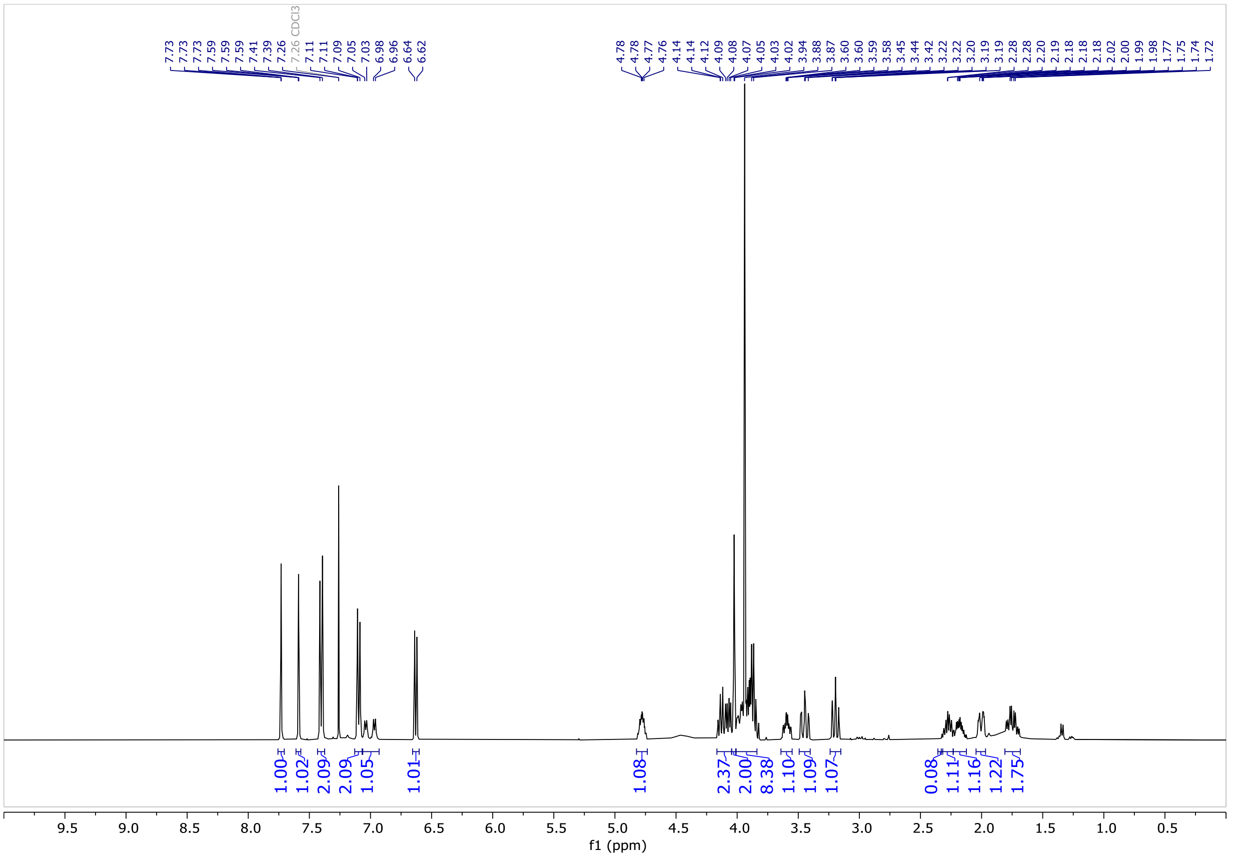

^{1}H NMR (400 MHz, $CDCl_3$) δ 7.73 (s, 1H), 7.59 (d, *J* = 0.9 Hz, 1H), 7.44 – 7.37 (m, 2H), 7.13 – 7.07 (m, 2H), 7.00 (dd, *J* = 28.5, 6.5 Hz, 1H), 6.63 (d, *J* = 7.4 Hz, 1H), 4.83 – 4.72 (m, 1H), 4.17 – 4.05 (m, 2H), 4.02 (s, 2H), 4.01 – 3.84 (m, 8H), 3.59 (tdd, *J* = 9.7, 5.0, 1.8 Hz, 1H), 3.50 – 3.39 (m, 1H), 3.25 – 3.14 (m, 1H), 2.33 – 2.23 (m, 1H), 2.23 – 2.13 (m, 1H), 2.06 – 1.95 (m, 1H), 1.75 (qd, *J* = 12.3, 4.8 Hz, 1H).[a] *OH proton is not observable.

[a] Integral value shown in the spectrum is higher than expected. This is due to the peak enhancement caused by overlapping with the residual water peak.

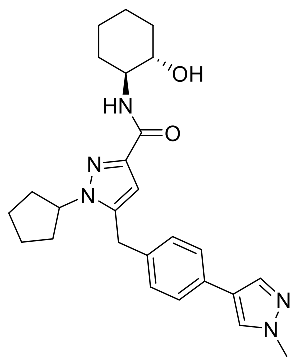

17k

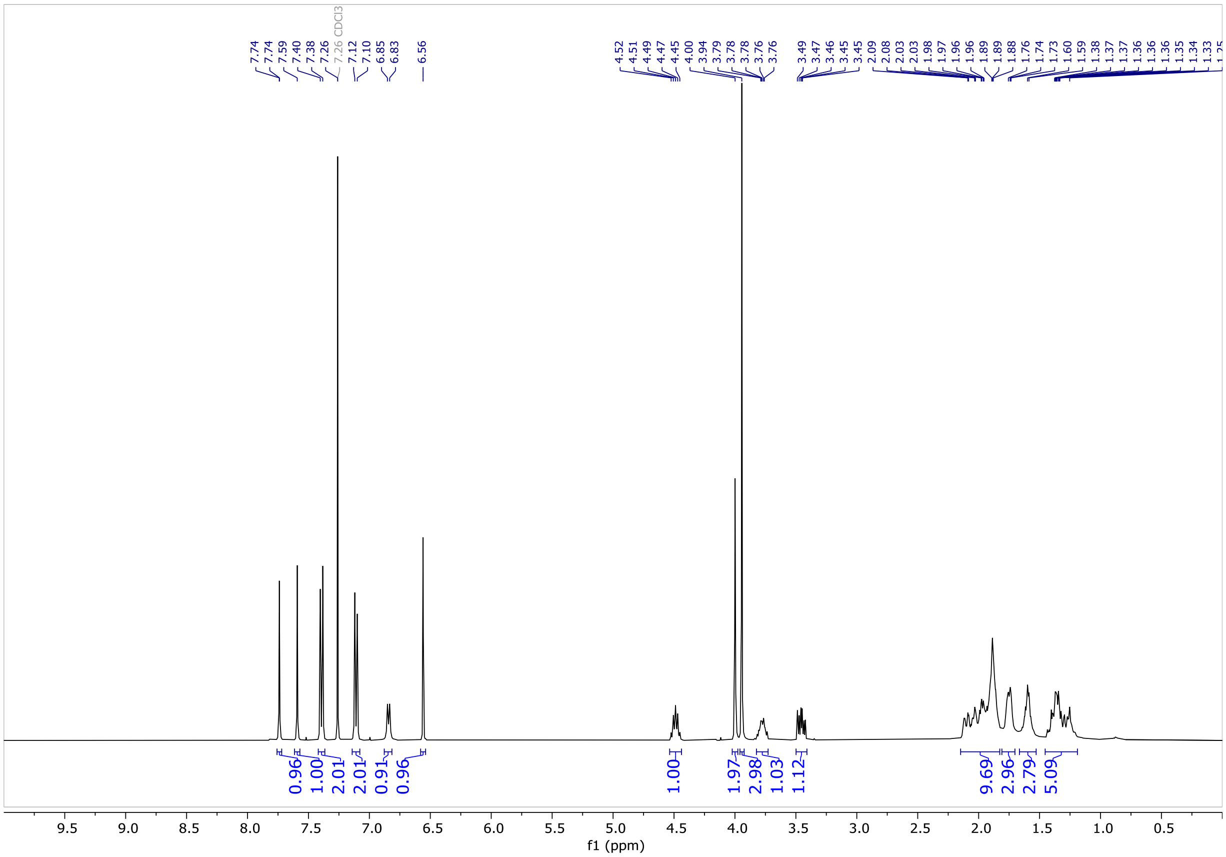

^{1}H NMR (400 MHz, $CDCl_3$) δ 7.74 (d, *J* = 0.8 Hz, 1H), 7.59 (s, 1H), 7.39 (d, *J* = 8.1 Hz, 2H), 7.11 (d, *J* = 8.2 Hz, 2H), 6.84 (d, *J* = 7.3 Hz, 1H), 6.56 (s, 1H), 4.49 (p, *J* = 7.2 Hz, 1H), 4.00 (s, 2H), 3.94 (s, 3H), 3.86 – 3.71 (m, 1H), 3.50 – 3.41 (m, 1H), 2.15 – 1.82 (m, 8H)[a], 1.81 – 1.70 (m, 2H)[a], 1.66 – 1.53 (m, 2H)[a], 1.46 – 1.19 (m, 4H).[a] *OH proton is not observable.

---

[a] Integral value shown in the spectrum is higher than expected. This is due to the peak enhancement caused by overlapping with the residual water peak.

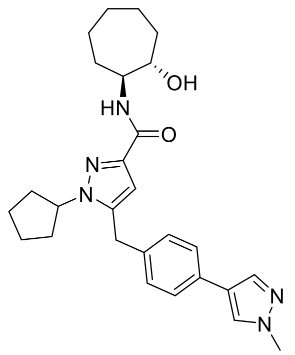

17l

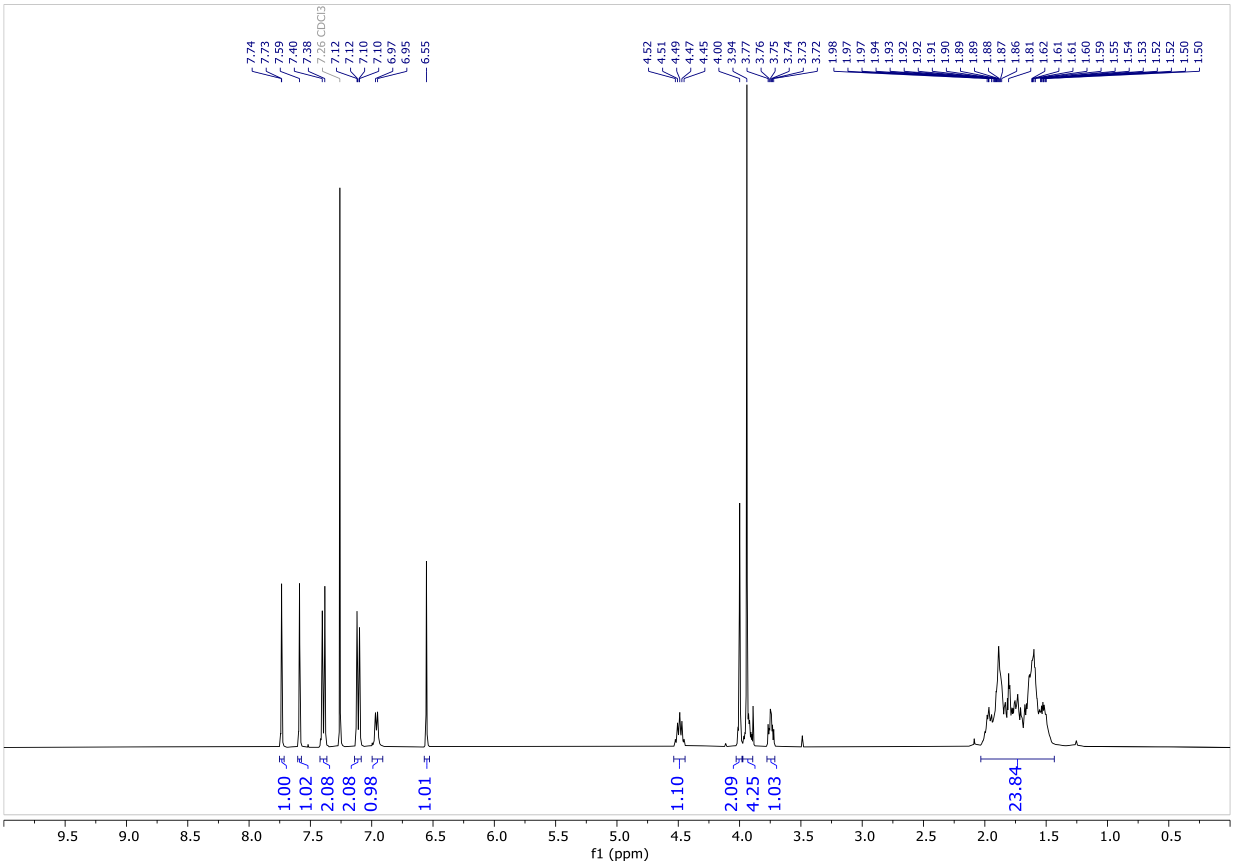

^{1}H NMR (400 MHz, $CDCl_3$) δ 7.74 (d, *J* = 0.8 Hz, 1H), 7.59 (s, 1H), 7.39 (d, *J* = 8.2 Hz, 2H), 7.11 (d, *J* = 8.2 Hz, 2H), 6.96 (d, *J* = 6.9 Hz, 1H), 6.55 (s, 1H), 4.49 (p, *J* = 7.1 Hz, 1H), 4.00 (s, 2H), 3.97 – 3.89 (m, 4H), 3.78 – 3.71 (m, 1H), 2.04 – 1.47 (m, 18H).[a]

[a] Integral value shown in the spectrum is higher than expected. This is due to the peak enhancement caused by overlapping with the residual water peak.

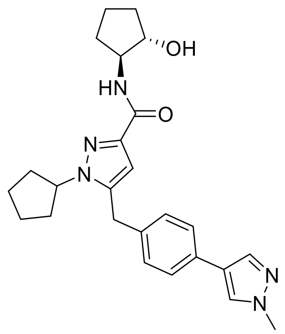

17m

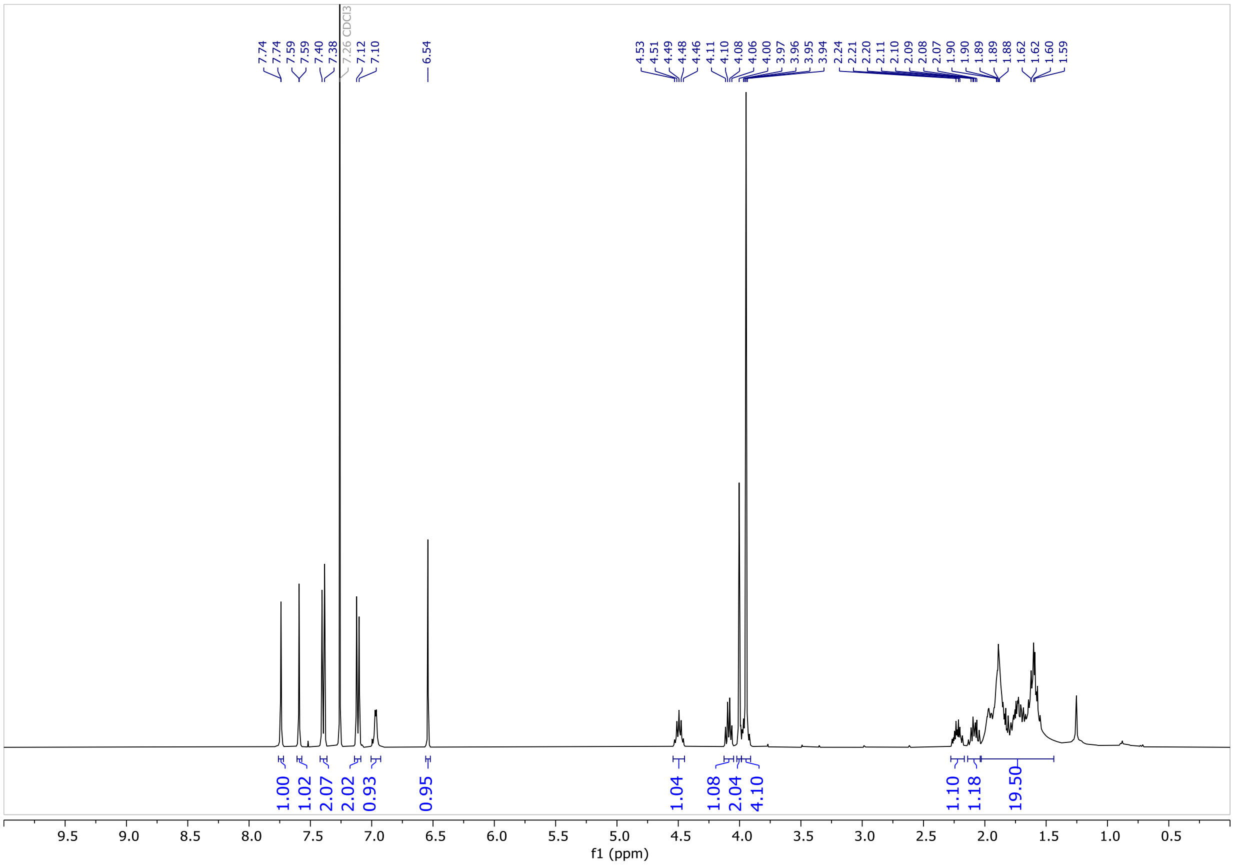

^{1}H NMR (400 MHz, $CDCl_3$) δ 7.74 (d, *J* = 0.8 Hz, 1H), 7.59 (d, *J* = 0.8 Hz, 1H), 7.39 (d, *J* = 8.2 Hz, 2H), 7.11 (d, *J* = 8.2 Hz, 2H), 6.97 (d, *J* = 4.4 Hz, 1H), 6.54 (s, 1H), 4.49 (p, *J* = 7.2 Hz, 1H), 4.09 (q, *J* = 6.8 Hz, 1H), 4.00 (s, 2H), 3.98 – 3.91 (m, 4H), 2.29 – 2.16 (m, 1H), 2.14 – 2.04 (m, 1H), 2.03 – 1.44 (m, 12H).[a] *OH proton is not observable.

[a] Integral value shown in the spectrum is higher than expected. This is due to the peak enhancement caused by overlapping with the residual water peak.

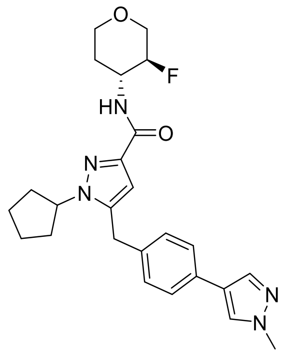

17n

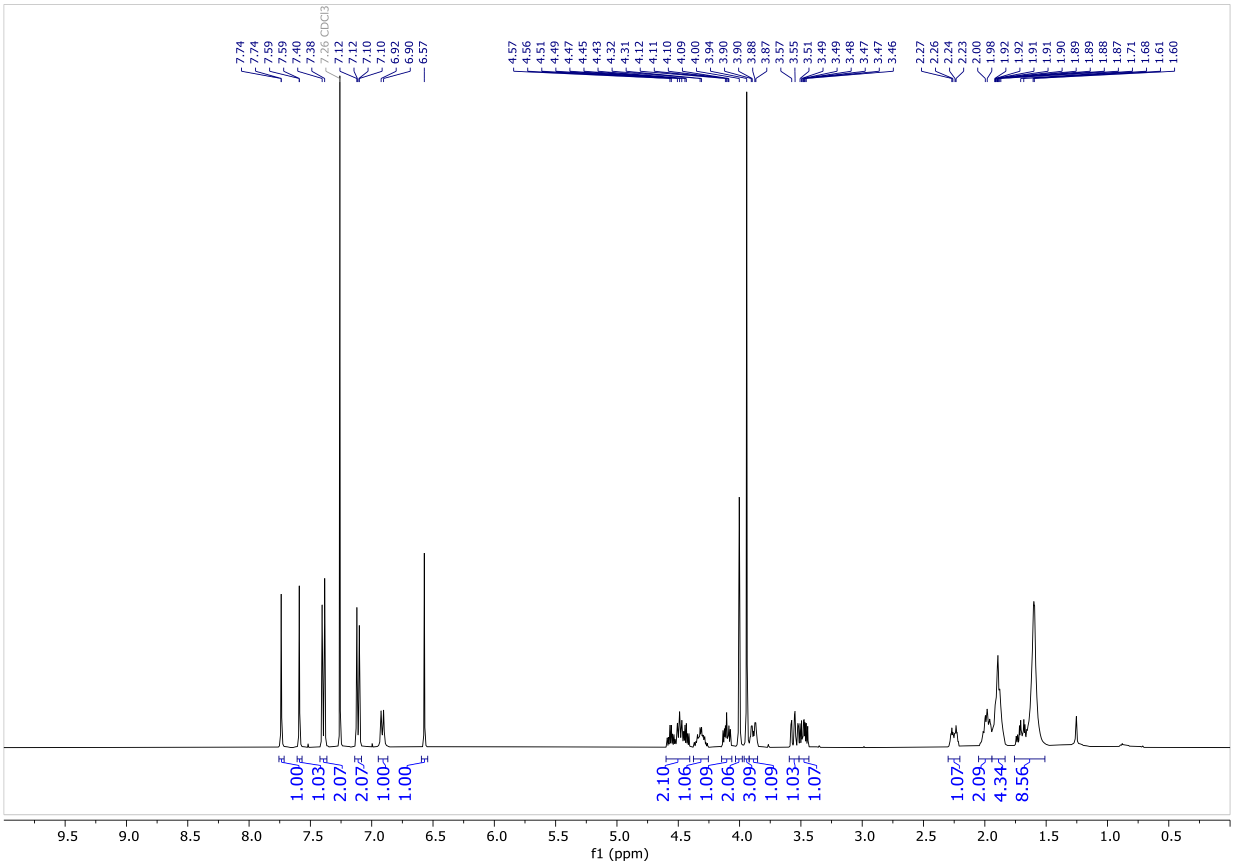

^{1}H NMR (400 MHz, $CDCl_3$) δ 7.74 (d, *J* = 0.9 Hz, 1H), 7.59 (d, *J* = 0.8 Hz, 1H), 7.39 (d, *J* = 8.2 Hz, 2H), 7.11 (d, *J* = 8.3 Hz, 2H), 6.91 (d, *J* = 8.0 Hz, 1H), 6.57 (s, 1H), 4.61 – 4.39 (m, 2H), 4.39 – 4.25 (m, 1H), 4.10 (dd, *J* = 7.8, 3.6 Hz, 1H), 4.00 (s, 2H), 3.94 (s, 3H), 3.92 – 3.85 (m, 1H), 3.60 – 3.52 (m, 1H), 3.51 – 3.44 (m, 1H), 2.31 – 2.19 (m, 1H), 2.05 – 1.94 (m, 2H), 1.94 – 1.82 (m, 4H), 1.76 – 1.54 (m, 3H).[a]

[a] Integral value shown in the spectrum is higher than expected. This is due to the peak enhancement caused by overlapping with the residual water peak.

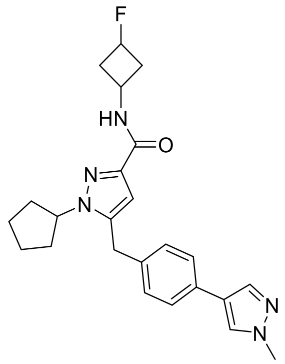

17o

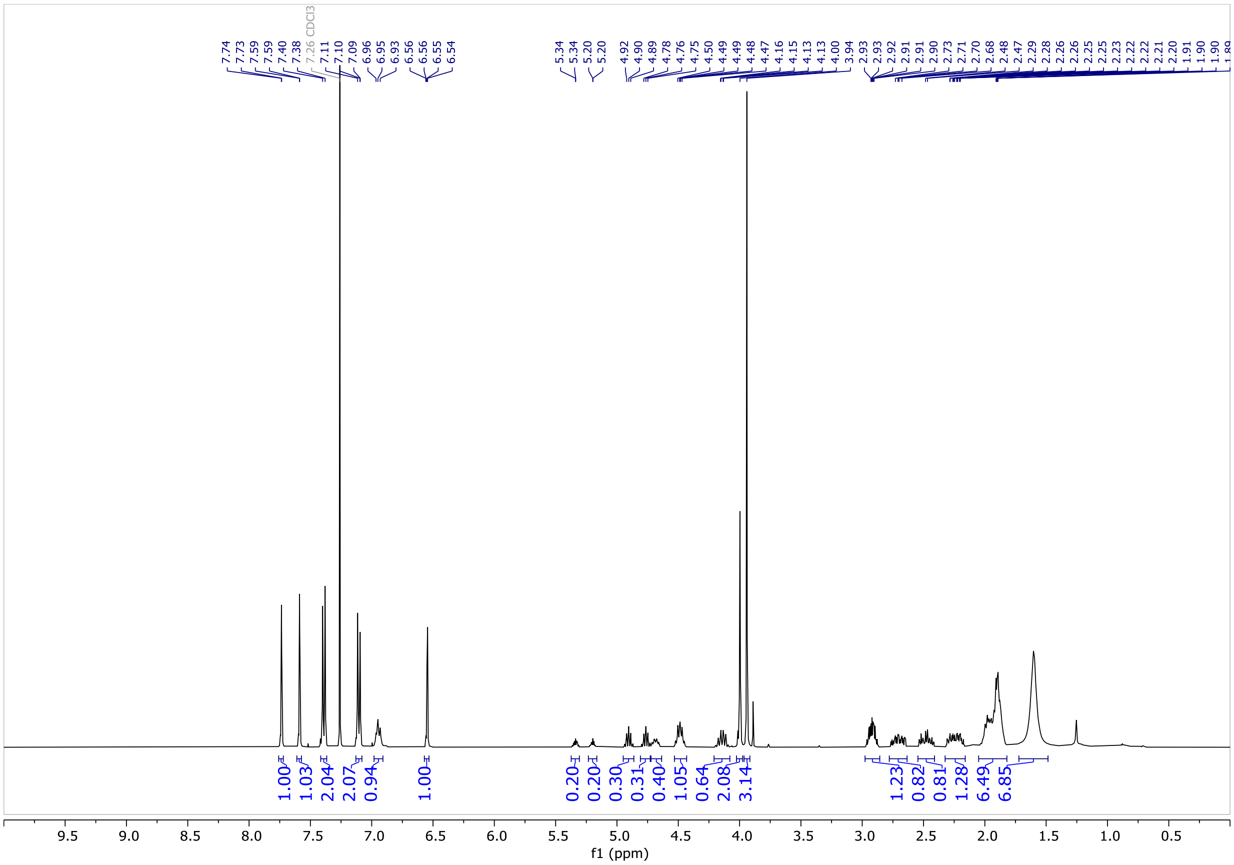

^{1}H NMR (400 MHz, $CDCl_3$) (a mixture of *cis/trans*) δ 7.74 (d, *J* = 0.8 Hz, 1H), 7.59 (d, *J* = 0.8 Hz, 1H), 7.39 (d, *J* = 8.1 Hz, 2H), 7.10 (d, *J* = 8.4 Hz, 2H), 6.95 (t, *J* = 6.9 Hz, 1H), 6.58 – 6.52 (m, 1H), 5.37 – 5.31 (m, 0.2H), 5.23 – 5.16 (m, 0.2H), 4.95 – 4.85 (m, 0.3H), 4.81 – 4.73 (m, 0.3H), 4.72 – 4.64 (m, 0.5H), 4.56 – 4.42 (m, 1H), 4.22 – 4.07 (m, 0.5H), 4.00 (s, 2H), 3.94 (s, 3H), 2.98 – 2.86 (m, 1H), 2.79 – 2.61 (m, 1H), 2.48 (d, *J* = 6.3 Hz, 1H), 2.34 – 2.15 (m, 1H), 2.05 – 1.82 (m, 6H), 1.72 – 1.48 (m, 2H).[a]

---

[a] Integral value shown in the spectrum is higher than expected. This is due to the peak enhancement caused by overlapping with the residual water peak.

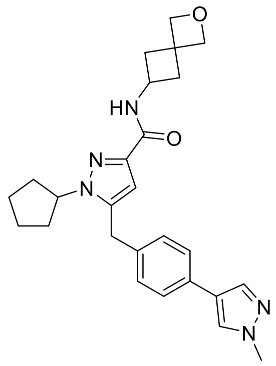

17p

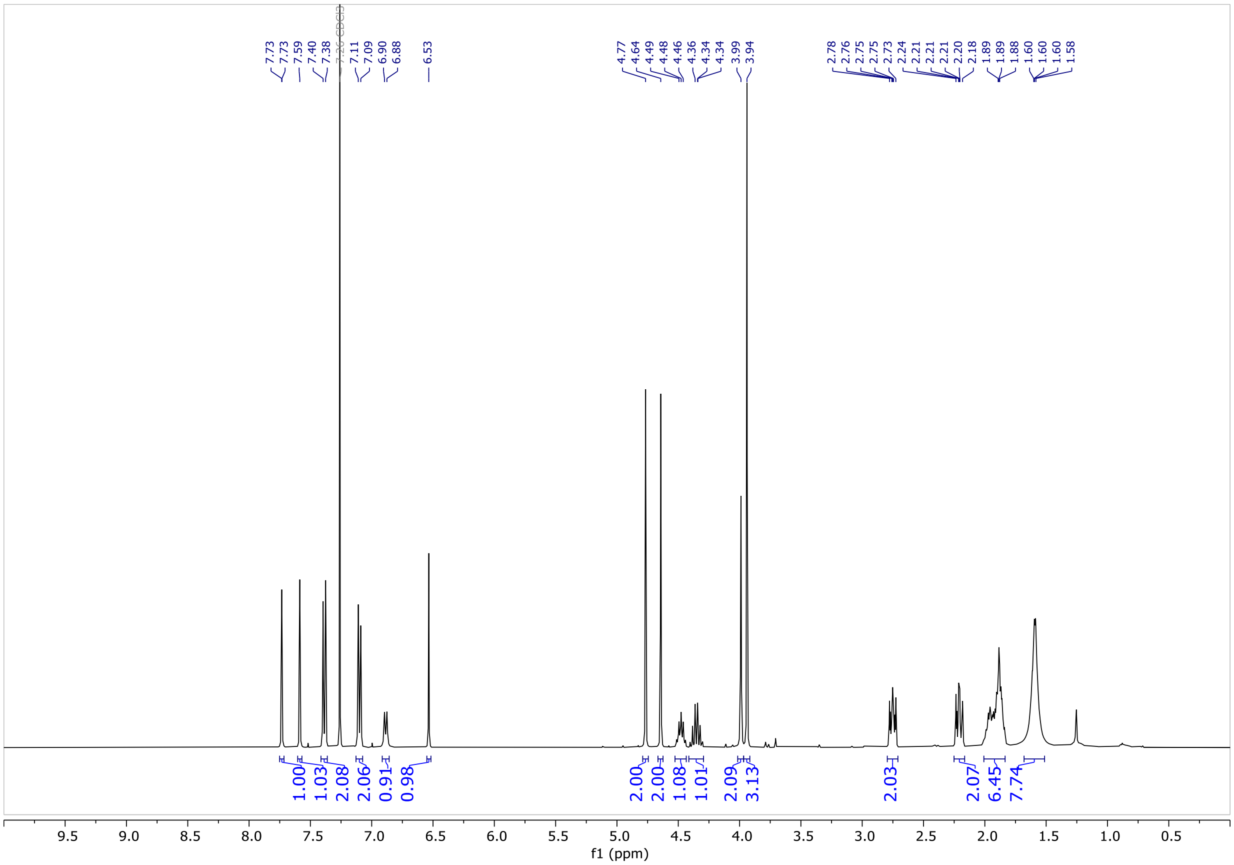

^{1}H NMR (400 MHz, $CDCl_3$) δ 7.73 (d, *J* = 0.8 Hz, 1H), 7.59 (s, 1H), 7.39 (d, *J* = 8.2 Hz, 2H), 7.10 (d, *J* = 8.2 Hz, 2H), 6.89 (d, *J* = 7.9 Hz, 1H), 6.53 (s, 1H), 4.77 (s, 2H), 4.64 (s, 2H), 4.54 – 4.42 (m, 1H), 4.41 – 4.30 (m, 1H), 3.99 (s, 2H), 3.94 (s, 3H), 2.81 – 2.70 (m, 2H), 2.26 – 2.15 (m, 2H), 2.01 – 1.83 (m, 6H), 1.68 – 1.51 (m, 2H).[a]

[a] Integral value shown in the spectrum is higher than expected. This is due to the peak enhancement caused by overlapping with the residual water peak.

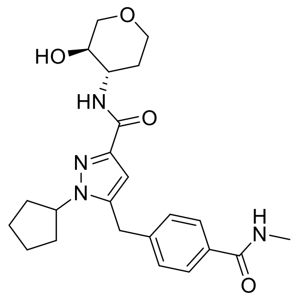

21

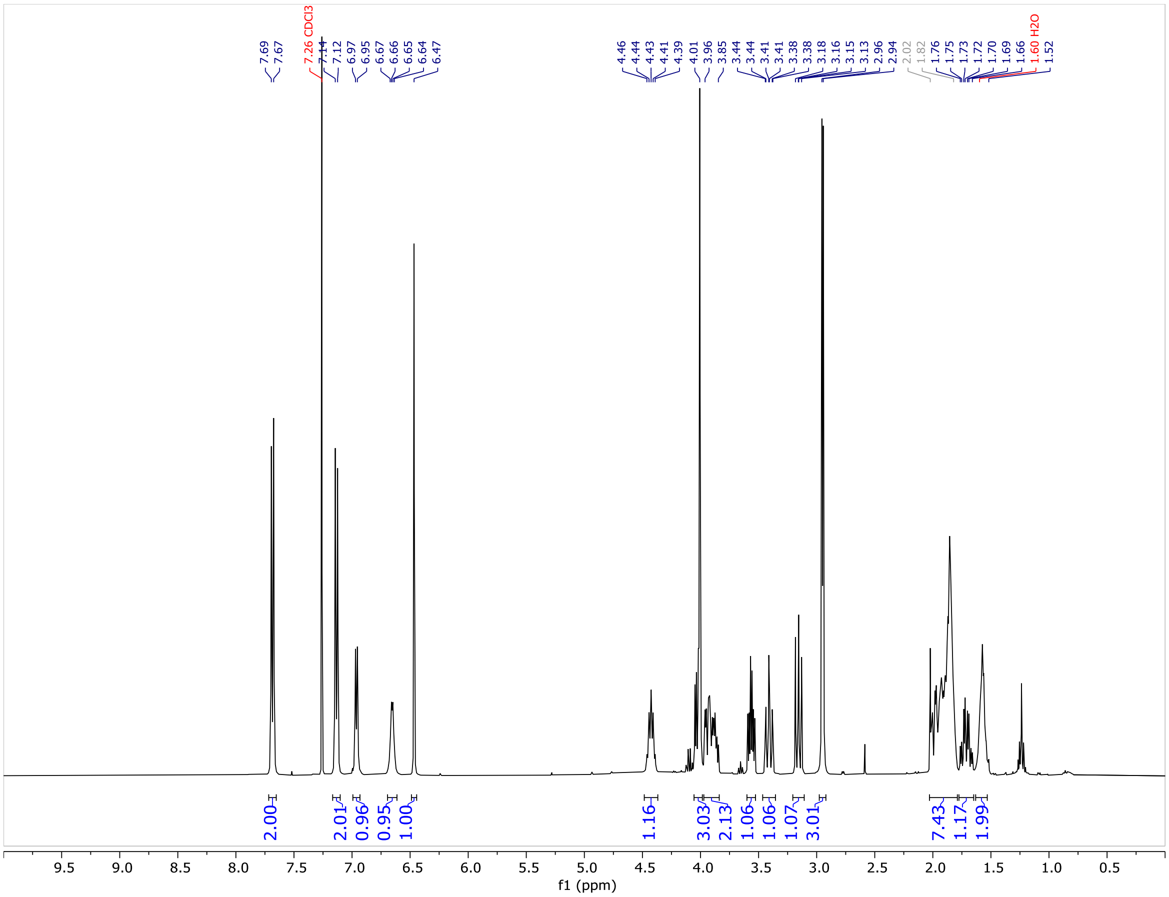

^{1}H NMR (400 MHz, $CDCl_3$) δ 7.68 (d, *J* = 8.1 Hz, 2H), 7.13 (d, *J* = 8.0 Hz, 2H), 6.96 (d, *J* = 6.4 Hz, 1H), 6.65 (q, *J* = 4.8 Hz, 1H), 6.47 (s, 1H), 4.43 (p, *J* = 7.2 Hz, 1H), 4.07 – 4.00 (m, 3H), 3.99 – 3.82 (m, 2H), 3.56 (td, *J* = 9.6, 5.0 Hz, 1H), 3.41 (td, *J* = 11.9, 2.2 Hz, 1H), 3.16 (dd, *J* = 11.3, 9.9 Hz, 1H), 2.95 (d, *J* = 4.8 Hz, 3H), 2.06 – 1.78 (m, 7H), 1.71 (qd, *J* = 12.1, 4.8 Hz, 1H), 1.63 – 1.52 (m, 2H). *OH proton is not observable.

HRMS data

Structure	Cmpd	Chemical Formula	Ion	(TOF, ES+) Calc. mass	Found mass
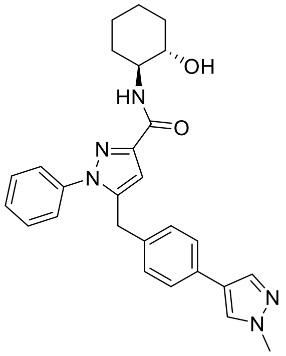	**17a**	$C_{27}H_{29}N_5O_2$	$(M+H)^+$	456.2394	456.2398
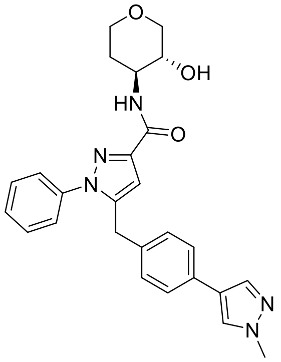	**17b**	$C_{26}H_{27}N_5O_3$	$(M+H)^+$	458.2187	458.2193
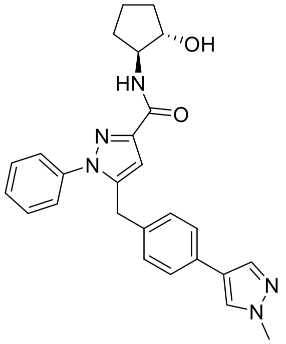	**17c**	$C_{26}H_{27}N_5O_2$	$(M+H)^+$	442.2238	442.2255

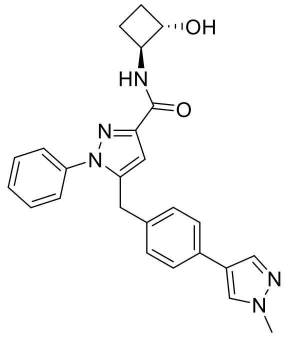	**17d**	$C_{25}H_{25}N_5O_2$	$(M+H)^+$	428.2081	428.2084
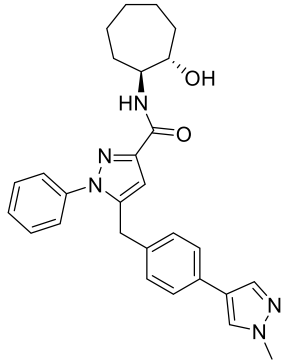	**17e**	$C_{28}H_{31}N_5O_2$	$(M+H)^+$	470.2551	470.2556
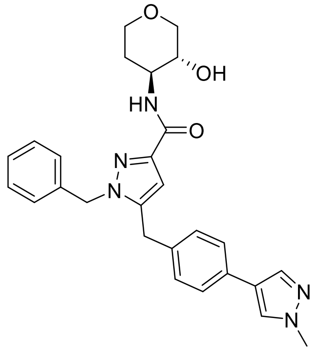	**17f**	$C_{27}H_{29}N_5O_3$	$(M+H)^+$	472.2343	472.2347
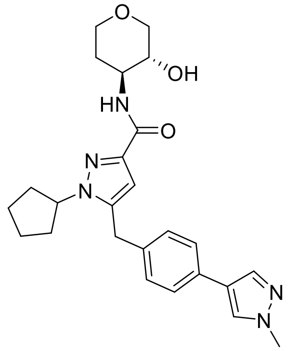	**17g**	$C_{25}H_{31}N_5O_3$	$(M+H)^+$	450.2500	450.2504

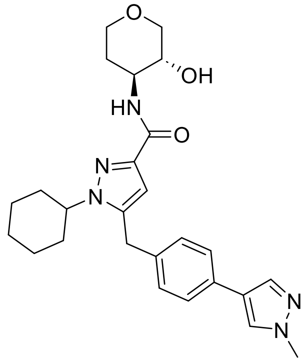	**17h**	$C_{26}H_{33}N_5O_3$	$(M+H)^+$	464.2656	464.2659
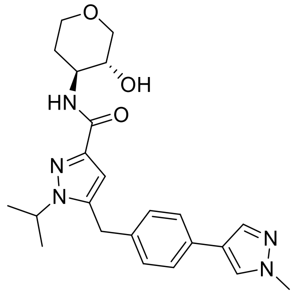	**17i**	$C_{23}H_{29}N_5O_3$	$(M+H)^+$	424.2343	424.2347
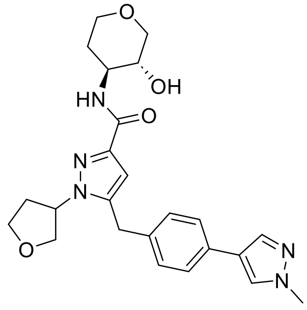	**17j**	$C_{24}H_{29}N_5O_4$	$(M+H)^+$	452.2292	452.2297
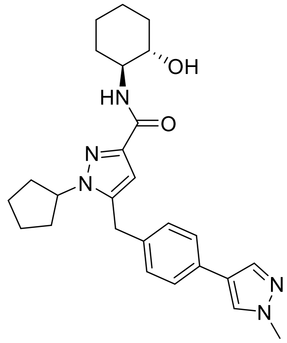	**17k**	$C_{26}H_{33}N_5O_2$	$(M+H)^+$	448.2707	448.2716

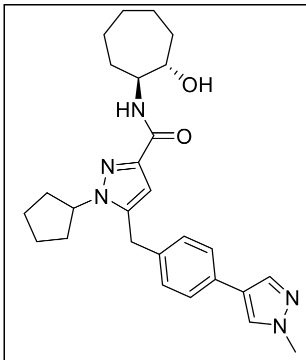	**17l**	$C_{27}H_{35}N_5O_2$	$(M+H)^+$	462.2864	462.2864
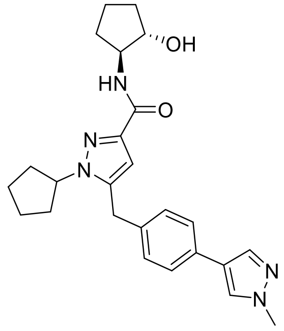	**17m**	$C_{25}H_{31}N_5O_2$	$(M+Na)^+$	456.2370	456.2367
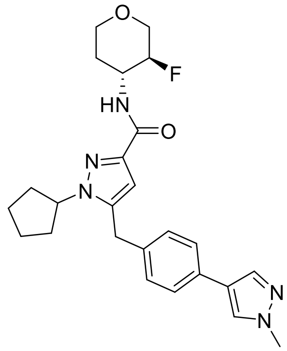	**17n**	$C_{25}H_{30}FN_5O_2$	$(M+H)^+$	452.2456	452.2463
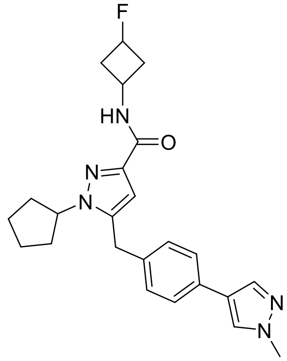	**17o**	$C_{24}H_{28}FN_5O$	$(M+H)^+$	422.2351	422.2353

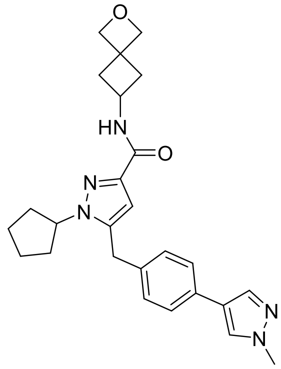	**17p**	$C_{26}H_{31}N_5O_2$	$(M+H)^+$	446.2551	446.2559
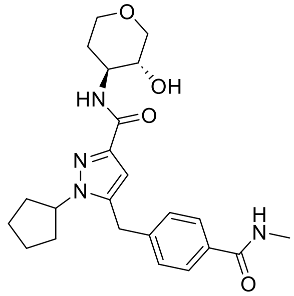	**21**	$C_{23}H_{30}N_4O_4$	$(M+H)^+$	427.2340	427.2342

Purity analysis by LC-MS

Structure	Cmpd	LC traces (215 nM and 254 nM)
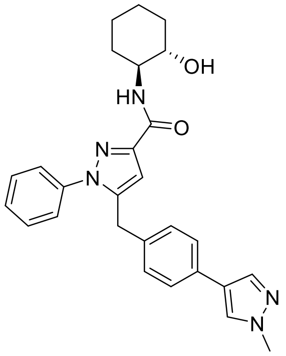	**17a**	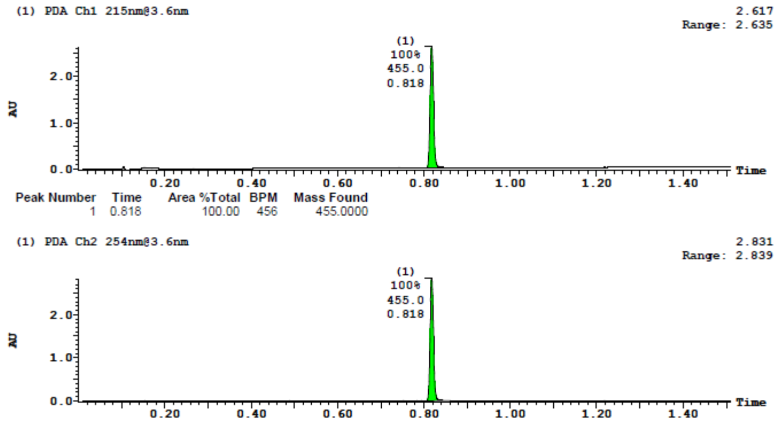
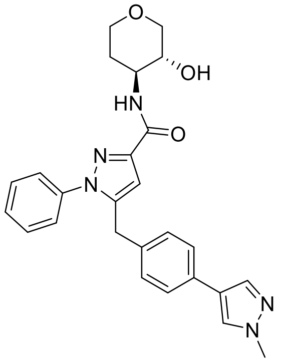	**17b**	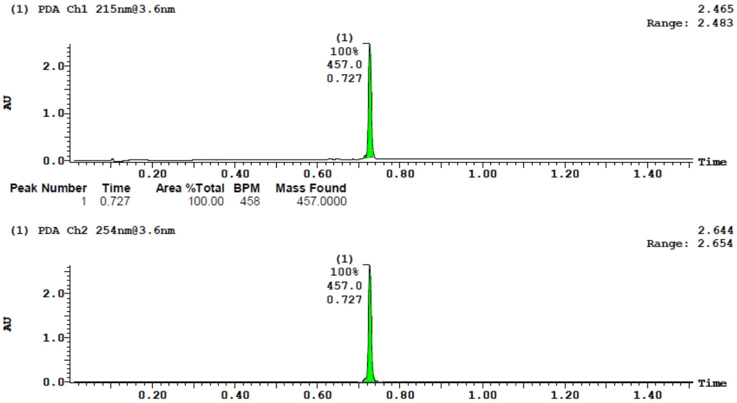
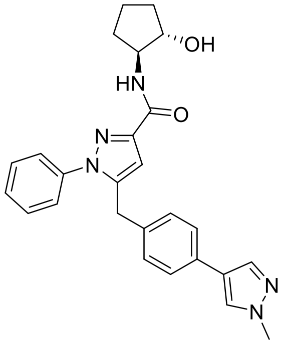	**17c**	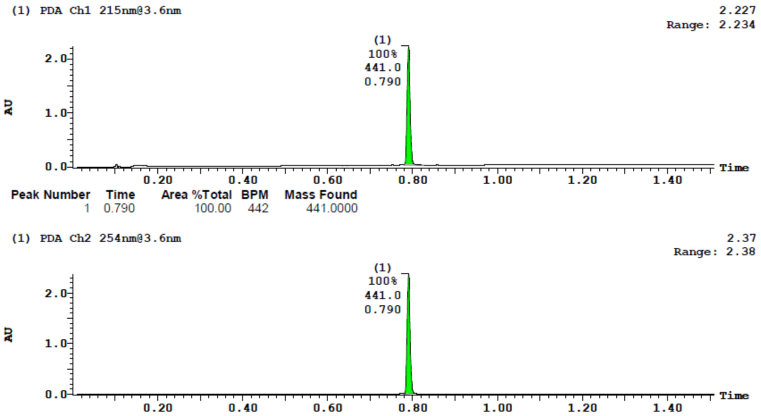

<table>
<tr><td>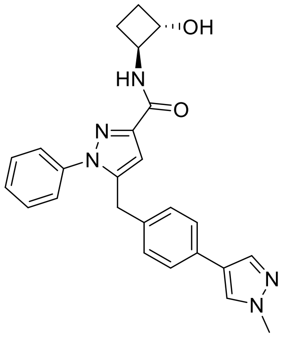</td><td>17d</td><td>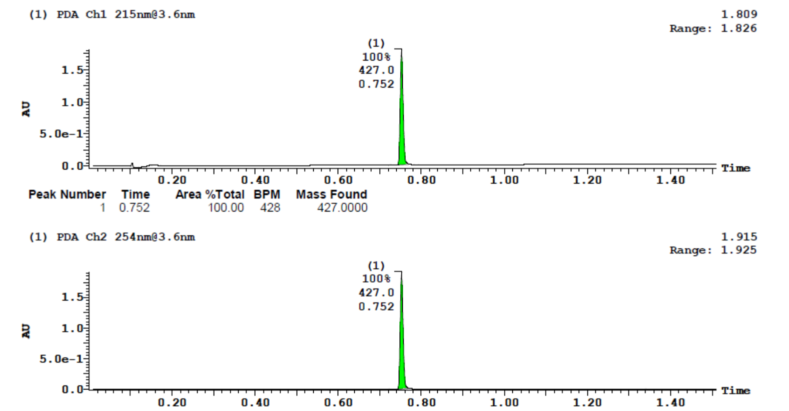
</td></tr>
<tr><td>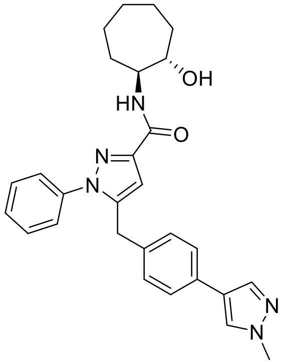</td><td>17e</td><td>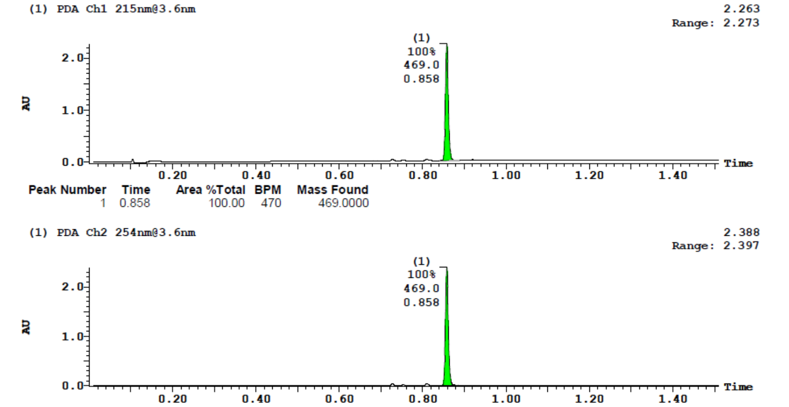
</td></tr>
<tr><td>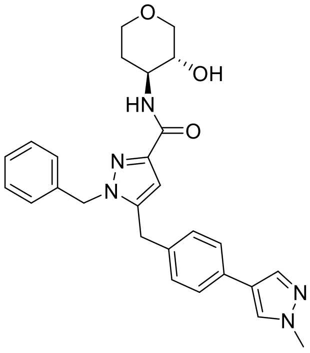</td><td>17f</td><td>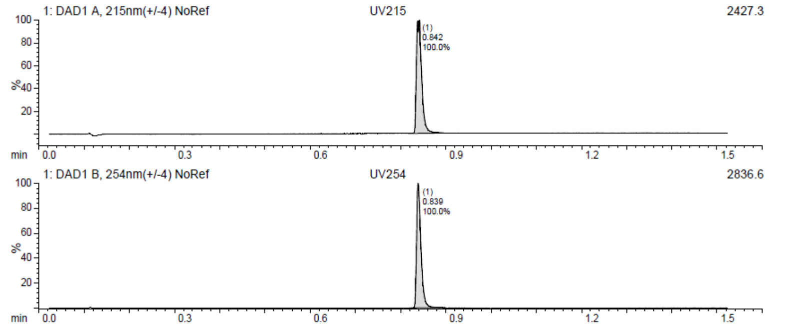
</td></tr>
<tr><td>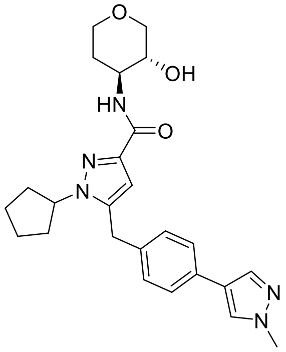</td><td>17g</td><td>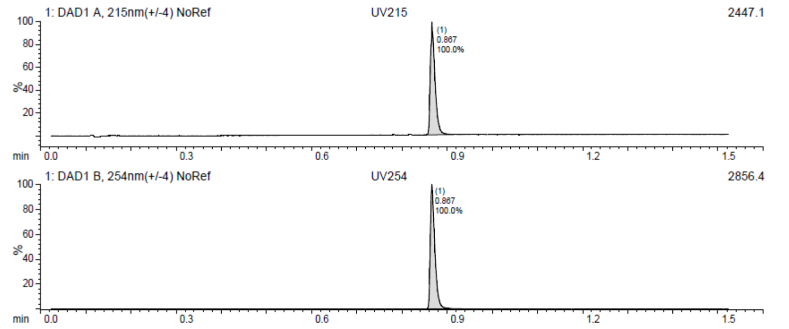
</td></tr>
</table>

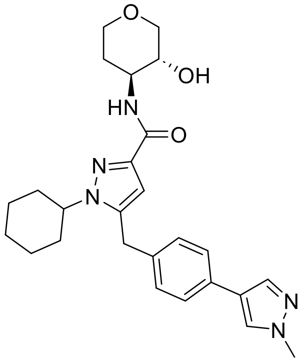	**17h**	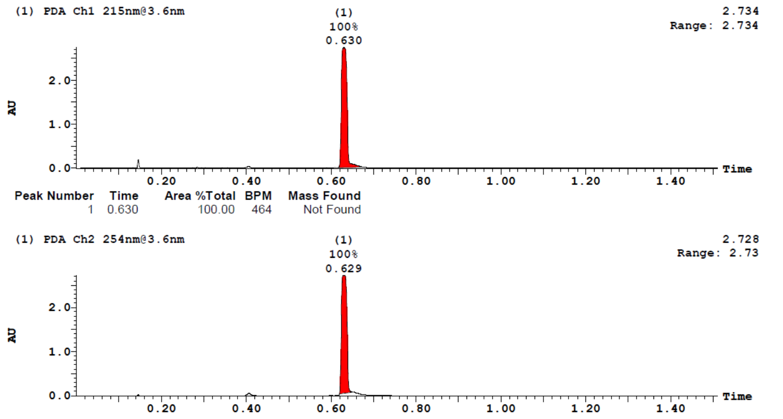
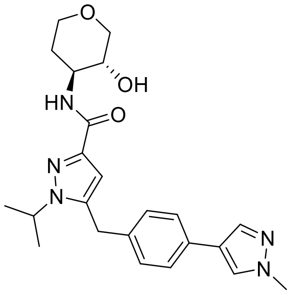	**17i**	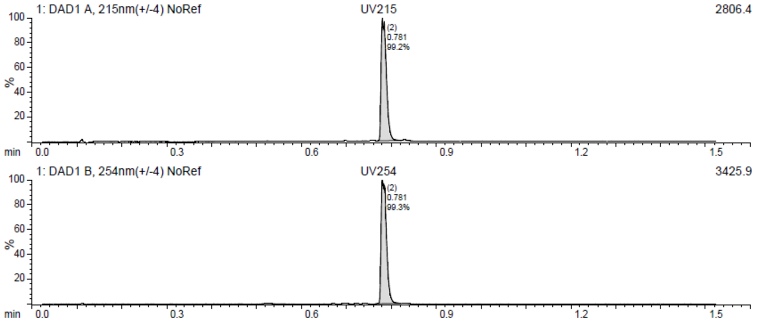
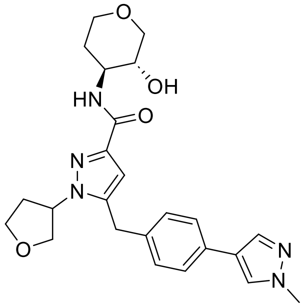	**17j**	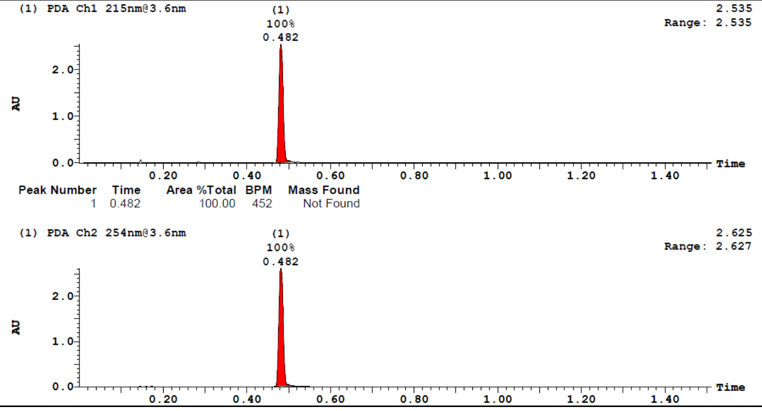
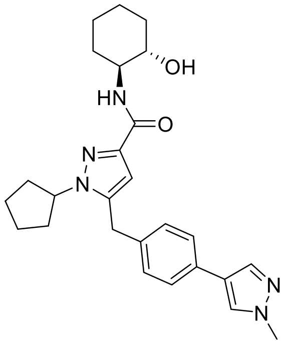	**17k**	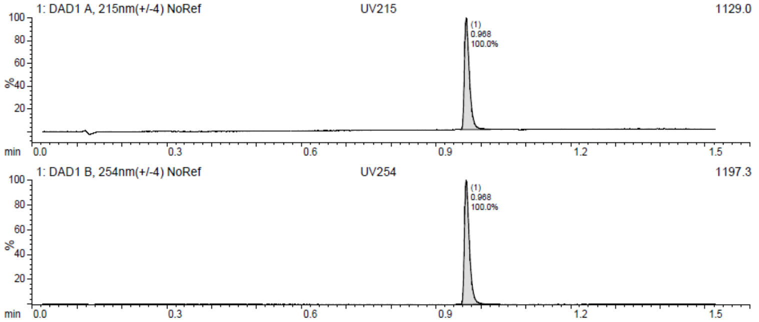

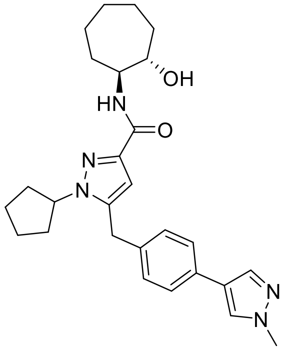	**17l**	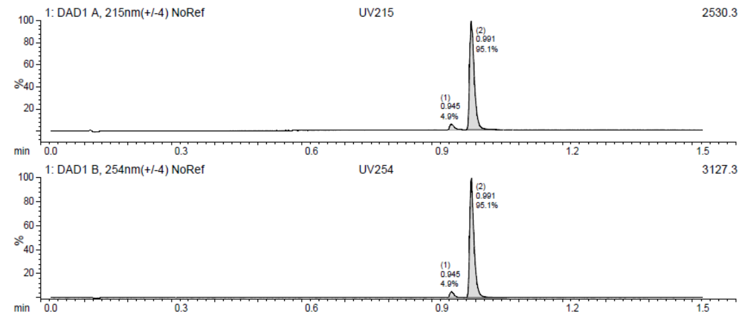
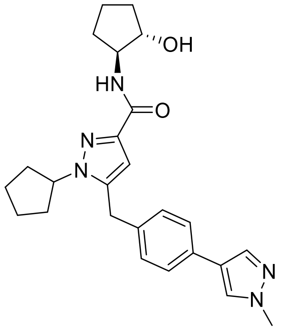	**17m**	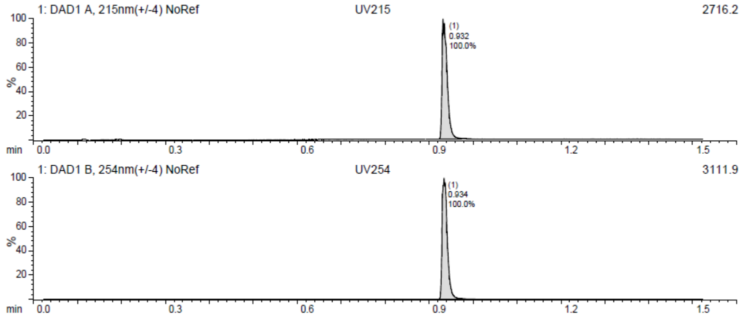
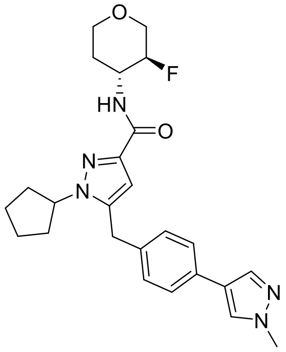	**17n**	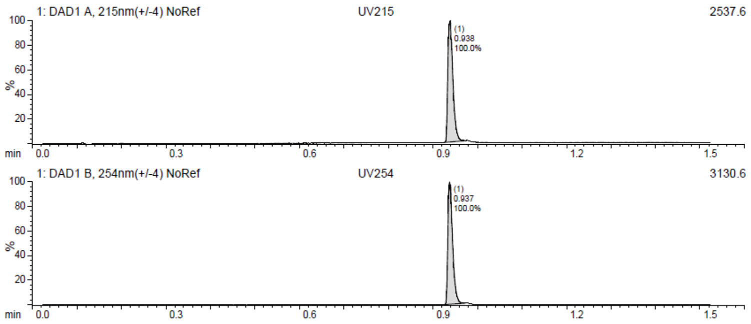
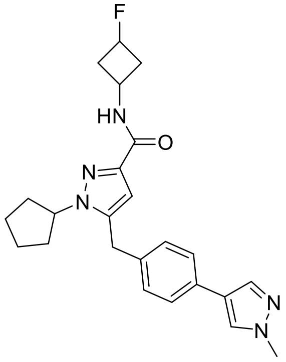	**17o**	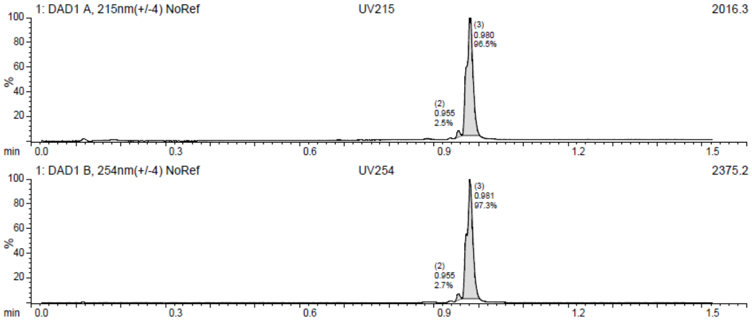

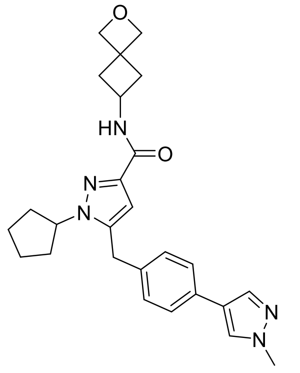	**17p**	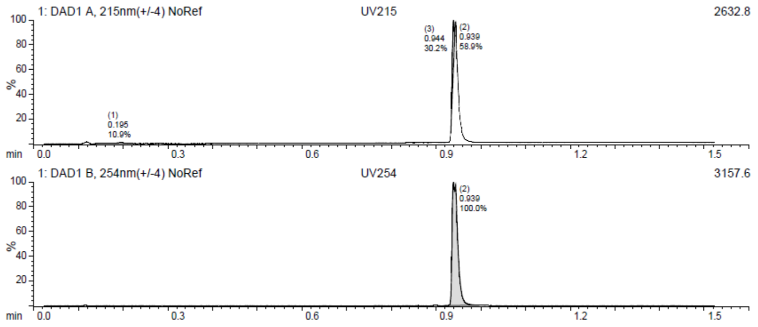1: DAD1 A, 215nm(+/-4) NoRef<br>UV215<br>2632.8<br>(1) 0.195 10.9%<br>(3) 0.944 30.2%<br>(2) 0.939 58.9%<br>1: DAD1 B, 254nm(+/-4) NoRef<br>UV254<br>3157.6<br>(2) 0.939 100.0%
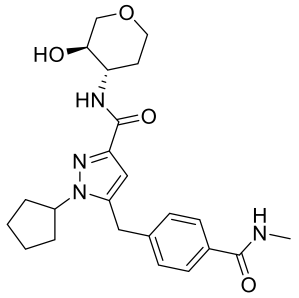	**21**	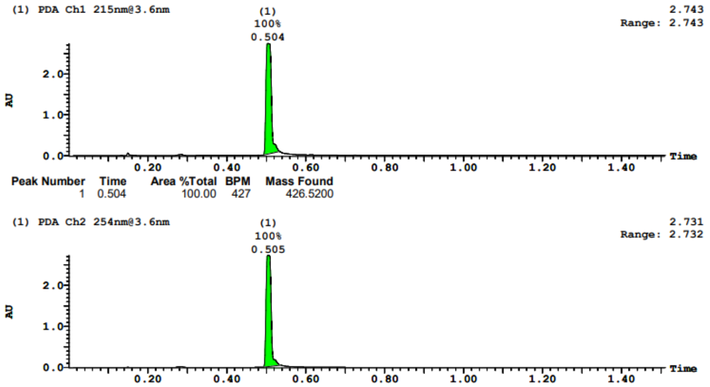(1) PDA Ch1 215nm@3.6nm<br>(1) 100% 0.504<br>2.743<br>Range: 2.743<br>Peak Number Time Area %Total BPM Mass Found<br>1 0.504 100.00 427 426.5200<br>(1) PDA Ch2 254nm@3.6nm<br>(1) 100% 0.505<br>2.731<br>Range: 2.732

References

1. A. Daina, O. Michielin, V. Zoete. SwissADME: a free web tool to evaluate pharmacokinetics, drug-likeness and medicinal chemistry friendliness of small molecules. Sci. Rep. **2017**; 7:42717. doi: 10.1038/srep42717.
